# Magnetic Nanoparticles: Synthesis and Applications in Life Sciences

**DOI:** 10.1002/open.202500214

**Published:** 2025-07-29

**Authors:** Kishore Chand, Erick S. Vasquez‐Guardado

**Affiliations:** ^1^ Department of Chemical and Materials Engineering University of Dayton 300 College Park Ave. Dayton OH 45469‐0256 USA

**Keywords:** agriculture, biomedical applications, hyperthermia, life sciences, magnetic nanoparticles

## Abstract

Magnetic nanoparticles (MNPs) are multifunctional materials with superparamagnetic properties and tunable surface chemistries, making them valuable across diverse fields, such as biomedicine, environmental remediation, and agriculture. This review examines recent advancements in MNP synthesis, encompassing chemical, physical, and environmentally friendly methods while highlighting improvements in size, morphology, and composition control that enhance application‐specific performance and environmental sustainability. The review discusses various architectures, including single‐core, core–shell, hybrid composites, and stimuli–responsive systems, with an emphasis on their stability, scalability, and functionalization potential. In biomedical applications, MNPs show promise in targeted drug delivery, magnetic hyperthermia, and magnetic resonance imaging contrast enhancement, where biocompatibility, often achieved through green synthesis, is critical. In agriculture, iron oxide MNPs (Fe_3_O_4_) have been utilized as nanofertilizers and growth promoters, demonstrating the ability to improve seed germination, chlorophyll content, and root development in crops, such as maize and tomatoes, without exhibiting phytotoxicity. Despite these promising results, challenges remain in large‐scale production, reproducibility, and regulatory acceptance. This review highlights the pivotal role of MNPs in advancing nanotechnology‐driven solutions across the life sciences. Their evolving synthesis techniques, multifunctional properties, and cross‐sector applications position MNPs as key enablers of next‐generation technologies in diagnostics, therapeutics, environmental monitoring, and sustainable agriculture.

## Graphical abstract

This review critically evaluates recent advancements in the synthesis, structural design, and application‐driven engineering of magnetic nanoparticles (MNPs) across biomedicine, environmental remediation, and agriculture. Emphasis is placed on sustainable synthesis approaches, morphological control, life science applications, and prevailing challenges in scalability and regulatory compliance, highlighting MNPs’ transformative potential in nanotechnology.

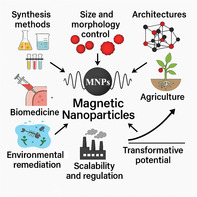

## 1 Introduction

Nanotechnology primarily involves the study and application of materials with dimensions between 1 and 100 nm, impacting fields, such as medicinal chemistry, materials science, and environmental engineering, among others.^[^ ^1^ ^]^ The development of this field relies on the fabrication, processing, and standardization of nanoscale materials, leading to significant scientific and technological advancements.^[^ ^2^ ^]^ A primary subject of nanomaterials is metal nanoparticles (NPs), which possess different shapes, high surface‐to‐volume ratios, and astonishing catalytic and antibacterial properties, making them valuable in medicine, water purification, renewable energy, and catalysis.^[^ ^2^ ^]^

Among metal NPs, magnetic NPs (MNPs) are a rapidly growing class of materials in which the unique combination of beneficial properties of NPs, such as high surface‐to‐volume ratio, superparamagnetism, size‐dependent properties, and quantum confinement effects, with traditional characteristics of bulk materials, has provoked a revolutionary development in material science. Incorporating MNPs within matrices, such as polymers, ceramics, or other metals, yields advanced nanocomposite materials with enhanced magneto‐responsive properties.^[^ ^3^ ^]^ MNP‐based nanocomposites, with their tunable mechanical, magnetic, and surface properties, are highly promising for biomedical, environmental, and electronic applications. They enable targeted drug delivery (TDD), imaging, and diagnostics in medicine, facilitate pollutant removal in environmental remediation, and enhance conductivity and sensing in advanced electronics.^[^ ^4^ ^]^

MNPs can be controlled and manipulated by outside magnetic fields. Some have unusual superparamagnetic traits.^[^ ^5^ ^]^ Superparamagnetism is a form of magnetism that occurs in small ferromagnetic or ferrimagnetic NPs, where the material does not have a net magnetization in the absence of an external magnetic field but becomes magnetized when such a field is applied due to thermal energy, which allows the rapid flipping of their magnetic moments.^[^ ^6^ ^]^ For example, superparamagnetic iron oxide NPs (SPIONs) are composed of Fe_3_O_4_ (magnetite) and have attracted considerable attention due to their excellent biocompatibility, rapid response time, and magnetic properties, which enable manipulation under various types of magnetic fields. Ferumoxytol is an FDA‐approved iron oxide nanoparticle formulation, specifically an intravenous product used for treating anemia in patients with chronic kidney disease. It is characterized as an ultrasmall iron oxide NP with a superparamagnetic core and a hydrophilic carboxymethyl–dextran coating. Due to its unique physicochemical properties and good safety profile, Ferumoxytol has demonstrated potential for various biomedical applications beyond its primary use, including magnetic resonance imaging (MRI),^[^ ^7^ ^]^ drug delivery,^[^ ^8^ ^]^ and immunotherapy.^[^ ^8^ ^]^

In addition to single‐core MNPs, the unique synergy of nanocomposites between the host matrix and the embedded MNPs imparts magnetic nanocomposites with exceptional functionalities, including superparamagnetic behavior, high magnetic susceptibility, and tunable surface chemistry. These properties enable applications in pharmaceutical delivery,^[^ ^9^ ^]^ cancer therapy,^[^ ^10^ ^]^ pollutant removal,^[^ ^11^ ^]^ and catalysis.^[^ ^12^ ^]^Advances in synthesis and characterization techniques have enhanced size and shape control, expanding their use in energy storage, biosensing, and oncology.

**Figure 1:**
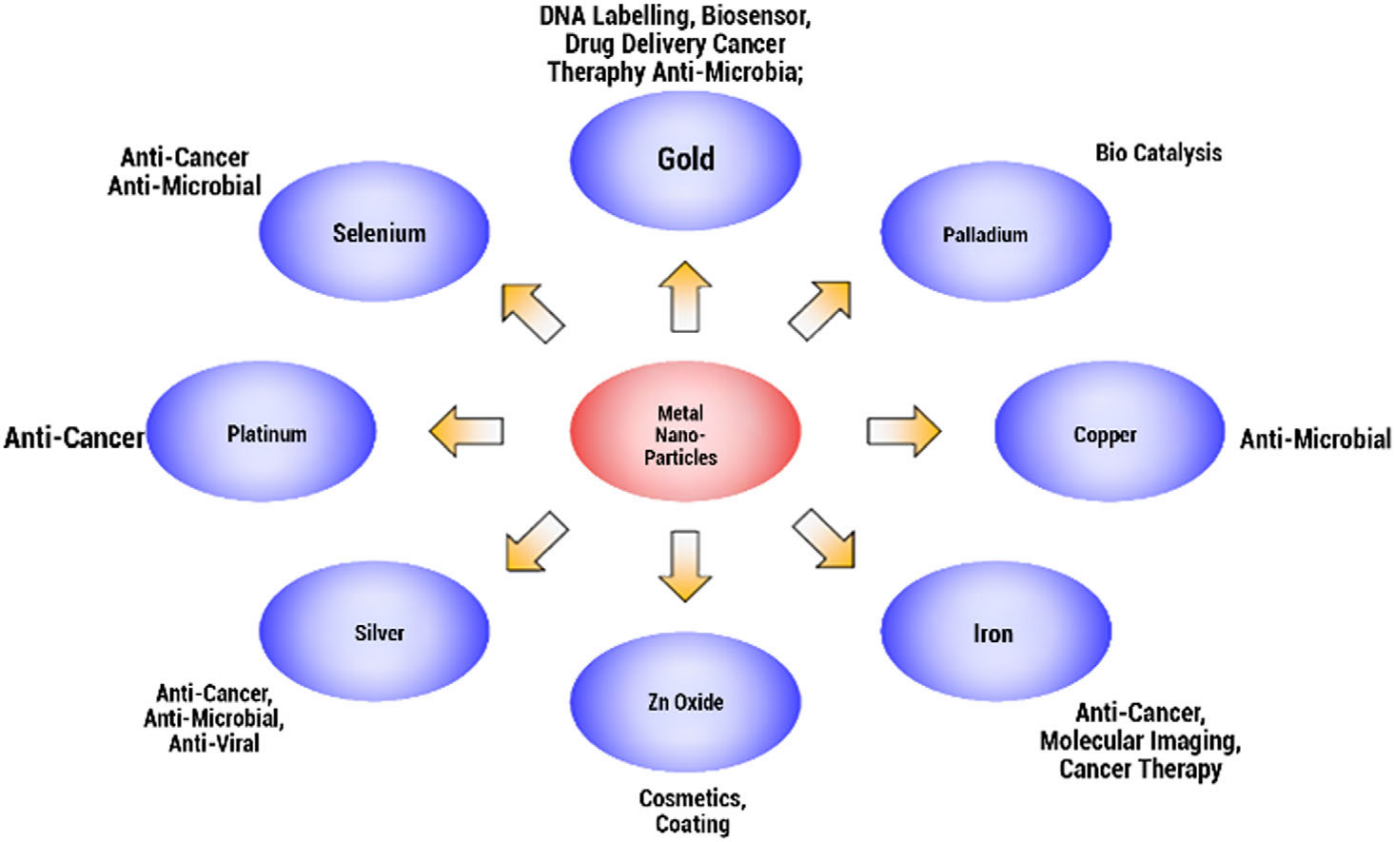
Metal nanoparticles and their applications. Reproduced (Adapted) with permission.^[^ ^245^ ^]^ Copyright 2013, Elsevier B.V.

As **Figure** **1** shows, there is a wide range of different types of metal NPs for numerous applications, emphasizing their versatility and potential impacts. For example, core–shell structures and the introduction of metal–organic frameworks (MOF) further improved such nanocomposites, opening up new avenues for research and applications.^[^ ^13^ ^]^

This review highlights recent advances in MNP synthesis and applications, focusing on their biomedical potential and applications in the life sciences. Given their polyvalent nature, this work also explores the evolving role of MNPs from simple colloids to key solutions for contemporary healthcare, agriculture, food protection, and technological challenges.

## 2 MNPs

MNPs exhibit unique properties, such as superparamagnetism, which have made them irreplaceable in several applications related to biomedicine, environmental remediation, and electronics. Usually, their sizes range between 1 and 100 nm, with high surface‐to‐volume ratios and quantum effects enabling novel applications that cannot be realized with bulk materials. The synthesis methods for MNPs include physical, chemical, and biological approaches; of these, ecofriendly synthesis methods are attracting considerable interest. The main types are SPIONs, ferromagnetic and ferrimagnetic NPs, core–shell structures, and magnetic alloys. While all these NPs have their advantages, the problems of aggregation and stability persist, prompting ongoing research efforts in the functionalization and refinement of synthesis methods.^[^ ^14^ ^]^

Also, iron oxide NPs (IONPs) have been extensively studied for their significant potential in biomedical and life science applications. More recently, research has explored the use of water‐soluble IONPs to trace iron uptake in plants. For instance, studies have demonstrated different uptake behaviors between 10 and 20 nm IONP–EDTA particles in plants, with larger particles (20 nm) leading to increased biomass and chlorophyl production. Iron NPs, particularly Fe_3_O_4_ NPs, have significant applications in environmental remediation due to their unique properties. They are recognized for their strong magnetic properties, which make them ideal candidates for biotechnological and biomedical applications, as well as for removing pollutants from the environment. Fe_3_O_4_ NPs have been found to exhibit good biocompatibility, which is comparable to or sometimes better than that of other metal oxide NPs. However, they may be more cytotoxic than gold‐coated IONPs, allowing them to participate in various biological activities.^[^ ^15^, ^16^ ^]^ They can also act as electron conduits in microbial processes, enhancing the degradation of organic pollutants and the removal of heavy metal ions from contaminated environments. Research indicates that the application of Fe_3_O_4_ NPs can effectively separate and remove heavy metals from suspension when external magnetic fields are applied, which also reduces toxicity and accumulation of these metals in plants. Furthermore, iron NPs can be synthesized through various methods, including the reduction of hematite or goethite, resulting in particles with high FeO content and specific morphologies.^[^ ^17^, ^18^, ^19^ ^]^

### 2.1 Approaches to Fabrication/Synthesis of Magnetic NPs and Nanocomposites

**Figure 2:**
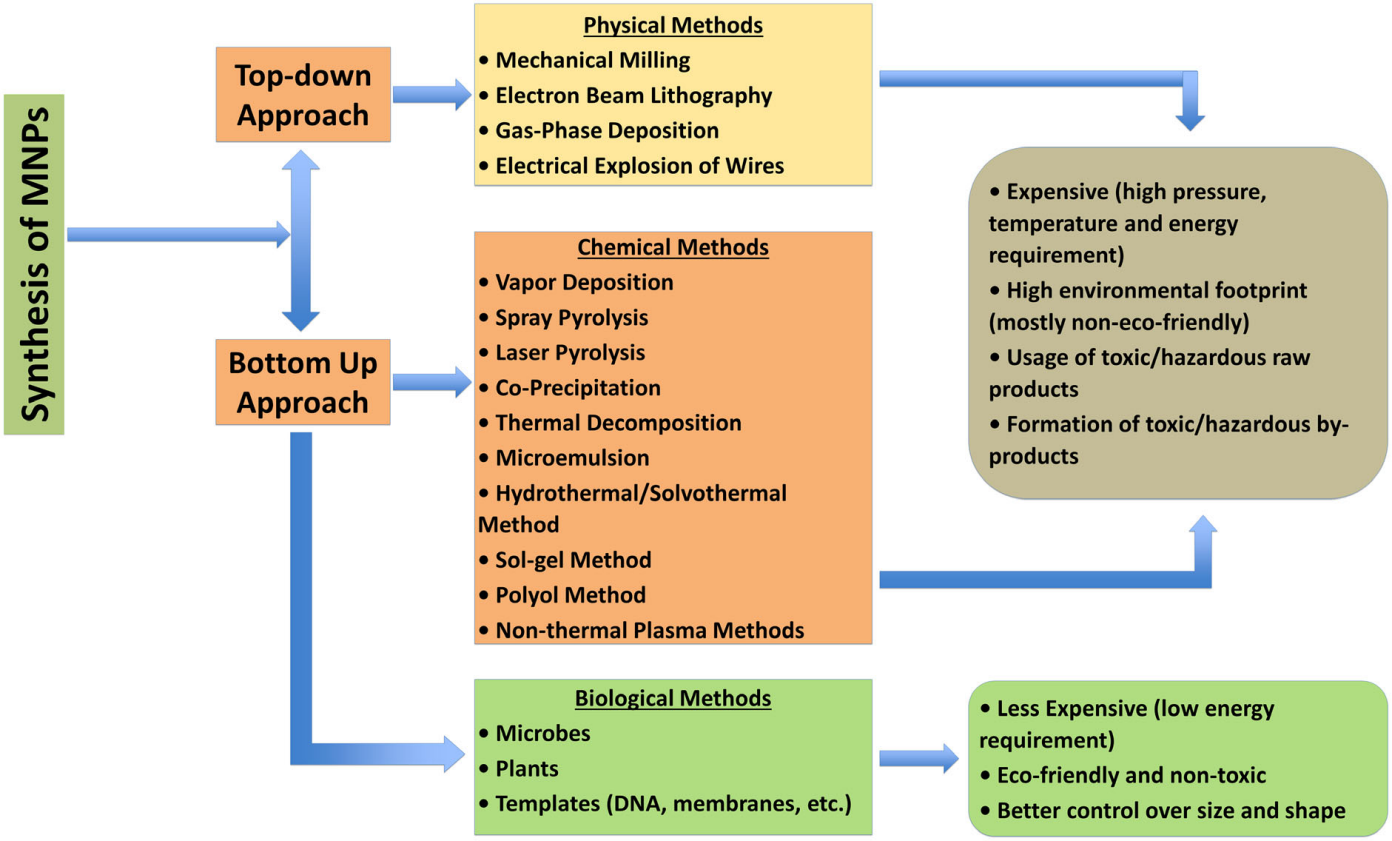
Synthesis method of nanomaterials. Reproduced (Adapted) with permission.^[^ ^246^ ^]^ Copyright 2021, Elsevier B.V.

MNPs are primarily synthesized through top‐down and bottom‐up approaches, further categorized into physical, chemical, and ecofriendly (biological) methods, as shown in **Figure** **2**. Techniques, such as microwave heating,^[^ ^20^ ^]^ hydrothermal synthesis,^[^ ^21^ ^]^ sol‐gel processing,^[^ ^22^ ^]^ and oxidation–reduction reactions, are commonly employed, with plant extract‐based synthesis gaining prominence due to its ecocompatibility. A detailed analysis of these methods provides critical insights into their strengths and limitations, addressing the growing demand for greener synthesis alternatives.^[^ ^23^, ^24^ ^]^

### 2.2 Types of Magnetic NPs

Based on their magnetic behavior, MNPs are classified into SPIONs, ferromagnetic NPs, antiferromagnetic NPs, ferrimagnetic NPs, core–shell structures, magnetic alloys, and multifunctional composites.^[^ ^25^, ^26^ ^]^ SPIONs, known for their excellent biocompatibility and superparamagnetic properties, are considered one of the most important and intensively explored MNPs. Indeed, SPIONs exhibit superparamagnetic behavior, meaning they display a significantly enhanced magnetic response under an external magnetic field but lose all residual magnetization once the field is removed. Such a property prevents particle aggregation and enhances their stability, making SPIONs highly effective for biomedical applications, like MRI contrast enhancement, TDD, and hyperthermia‐based cancer therapy.^[^ ^25^ ^]^ Ferromagnetic NPs—commonly composed of metals such as iron, cobalt, or nickel—differ from SPIONs in that they retain remanent magnetization upon removal of an external magnetic field. This property enables their application in advanced magnetic separation techniques for life science diagnostics.^[^ ^26^, ^27^ ^]^ For example, iron‐based functionalized MNPs with antibodies enable rapid detection of pathogens in blood samples. These NPs bind to target pathogens (e.g., *Escherichia coli* or *Salmonella*), allowing isolation via permanent magnets without continuous external fields, streamlining automated diagnostic workflows.^[^ ^28^, ^29^ ^]^

Antiferromagnetic NP moments are aligned in opposite directions, yielding zero net magnetization. These are more rarely used in biomedical applications but can be useful under certain circumstances.^[^ ^30^ ^]^ Ferrimagnetic NPs possess magnetic properties intermediate between ferromagnetic and antiferromagnetic materials, with magnetite (Fe_3_O_4_) and maghemite (γ‐Fe_2_O_3_) being the most important examples.^[^ ^26^ ^]^ These NPs exhibit a strong magnetic response while maintaining biocompatibility, making them widely applicable in biomedical fields.^[^ ^31^ ^]^

Core–shell magnetic NPs consist of a magnetic core surrounded by a nonmagnetic shell, which enhances their stability, biocompatibility, and functionality.^[^ ^6^ ^]^ The shell can be composed of various materials, such as silica, gold, or polymers, thus enabling further surface functionalization and property tuning.^[^ ^32^ ^]^

Magnetic alloys and composites enable the tuning of magnetic properties by combining different magnetic materials or incorporating magnetic NPs into a nonmagnetic matrix.^[^ ^33^ ^]^ This approach facilitates the development of materials with enhanced magnetic, mechanical, or chemical properties tailored for specific applications. Permalloy, a nickel–iron magnetic alloy typically composed of about 80% nickel and 20% iron, exhibits high magnetic permeability and low coercivity. This alloy is an excellent example of how combining different magnetic materials can result in enhanced magnetic properties tailored for specific applications.^[^ ^34^ ^]^ Additionally, there are also advanced classes of MNPs, including multifunctional magnetic NPs, that integrate magnetic properties with other functionalities, such as optical, catalytic, or therapeutic capabilities.^[^ ^35^ ^]^ These NPs hold significant promise for theranostic applications, where diagnostic and therapeutic functions are combined into a single platform.^[^ ^36^, ^37^ ^]^

The applications of MNPs extend across diverse fields, ranging from biomedical therapies to environmental remediation. In cancer treatment, MNPs hold great potential for TDD, hyperthermia therapy, and MRI contrasting agents. By guiding MNPs using external magnetic fields, therapeutic agents can be precisely localized, minimizing systemic side effects while improving treatment efficacy. From an environmental perspective, MNPs have been utilized for removing pollutants from water and soil by leveraging their high surface area and magnetic separability.^[^ ^31^ ^]^ Despite their numerous advantages, challenges still remain in the development and application of magnetic NPs. These include concerns regarding long‐term stability, potential toxicity, and the need for more precise control over size distribution and magnetic properties. Current research focuses on improving synthesis methods, enhancing surface functionalization techniques, and understanding the interactions between MNPs and biological systems to address these challenges.^[^ ^26^, ^32^, ^35^ ^]^ Recent advances aim to enhance the properties of MNP systems, improve targeting capabilities, and ensure both biocompatibility and new functionalities. The integration of MNPs with other nanomaterials, stimuli–responsive polymers, and microfluidics devices is beginning to open new pathways into personalized medicine and next‐generation diagnostics. With ongoing research, magnetic NPs will continue to play a crucial role in addressing critical challenges in healthcare, environmental protection, and beyond.^[^ ^35^, ^38^ ^]^

### 2.3 Properties of Magnetic NPs

As previously discussed, magnetic NPs have attracted significant research interest due to their diverse applications, ranging from targeted cancer therapies to environmental remediation. In fact, their properties arise from their nanoscale dimensions and can be tailored for specific applications. Advances in synthesis and functionalization methods continue to expand their potential, enabling further innovations across various fields. In this section, we explore some of the most significant properties of MNPs.^[^ ^39^, ^40^ ^]^

#### 2.3.1 Superparamagnetism

A key property of MNPs includes superparamagnetism, which is typically observed in NPs smaller than 100 nm.^[^ ^26^ ^]^ Superparamagnetic MNPs exhibit no residual magnetism in the absence of an external field but become strongly magnetized when exposed to one, allowing for precise control in biological systems. This property is particularly valuable in applications, such as TDD in cancer therapy^[^ ^41^ ^]^ and magnetic separation techniques in environmental remediation.^[^ ^11^, ^42^ ^]^ Ha et al. reported that superparamagnetism occurs within a size range of a few to several tens of nanometers. In the case of ferromagnetic NPs, Dulińska‐Litewka et al. demonstrated that SPIONs smaller than 20 nm exhibit zero hysteresis and no residual magnetization.^[^ ^43^ ^]^ Kritika and Roy highlight that SPIONs are excellent MRI contrast agents due to their nontoxic nature and enhanced contrast capabilities. As mentioned earlier, one notable example of SPIONs is ferumoxytol, which is FDA‐approved for treating iron deficiency anemia but is also used off‐label as an MRI contrast agent. This off‐label use is particularly beneficial for patients who cannot tolerate gadolinium‐based contrast agents (GBCAs), such as those with renal insufficiency or a history of hypersensitivity reactions to GBCAs.^[^ ^44^ ^]^ Similarly, Pucci et al., highlighted their potential in magnetic hyperthermia (MHT) for cancer treatment, where exposure to an alternating magnetic field (AMF) induces heat generation.^[^ ^45^ ^]^ This further underscores the increasing importance of superparamagnetic NPs in biomedical applications, imaging, therapy, and environmental remediation.

#### 2.3.2 Size and Surface Area

The size of MNPs plays a crucial role in their effectiveness across various applications. Smaller NPs (10−100 nm) exhibit enhanced diffusion and distribution while minimizing macrophage capture, thereby improving their pharmacokinetics for cancer treatment and environmental remediation. Additionally, their high surface‐to‐volume ratio facilitates surface functionalization with biomolecules, increasing specificity and efficacy.^[^ ^46^ ^]^

Recent research further emphasizes the critical influence of size and surface area on the performance of MNPs. For instance, according to Meenakshi et al., large specific surface area, high reactivity, and reduced size are key factors in enhancing adsorption for contaminant removal.^[^ ^11^ ^]^ Similarly, Guerra demonstrated that optimizing nanomaterial performance in remediation can be achieved by modifying their size, morphology, and porosity.^[^ ^47^ ^]^ Recently, Zhang et al. further showed that the high surface area of MNPs enhances their adsorption capacity and detection of contaminants in soil and water.^[^ ^48^ ^]^ These findings reaffirm the crucial role of size and surface area in environmental applications while reflecting advancements in the field.

#### 2.3.3 Magnetic Susceptibility and Saturation Magnetization

The magnetic susceptibility and saturation magnetization of MNPs are key parameters that influence their performance across various applications. Iron oxide NPs, particularly magnetite (Fe_3_O_4_) and maghemite (γ‐Fe_2_O_3_), are widely used due to their high magnetic susceptibility and biocompatibility.^[^ ^49^ ^]^ Doping iron oxide NPs with metals such as zinc, cobalt, and silver increases magnetic and functional properties. For instance, NPs doped with zinc and cobalt exhibit improved antibacterial activity due to the increased generation of reactive oxygen species (ROS) and higher charge density.^[^ ^26^, ^50^ ^]^ In a study by Kasparis et al., Zn_0.4_Fe_2.6_O_4_ had a remarkably high saturation magnetization of 142 ± 9 emu g^−1^ (Normalized per gram of Fe^+^ and Zn^−1^ content) and enhanced photothermal efficiency through a 22% reduction in the bandgap.^[^ ^51^ ^]^ Similarly, yttrium and M^2+^ (Co, Ni, Mn)‐doped NPs have been shown to enhance MRI contrast, although toxicity challenges remain.^[^ ^52^ ^]^ Magnetic nanocomposites have also demonstrated promise in water remediation, offering efficient contaminant removal with easy magnetic recovery.

#### 2.3.4 Surface Chemistry and Functionalization

Surface chemistry is important for the detection and antibacterial activity of MNPs. Various coating strategies, such as silica coatings, have been developed to enhance stability, biocompatibility, and functionality, providing a platform for further functionalization.^[^ ^53^ ^]^ Advances in synthesis and functionalization techniques have significantly expanded MNP applications in biomedicine, environmental sciences, and more.^[^ ^54^ ^]^

Popescu et al. reviewed surface modification methods, emphasizing silica coatings for their ability to improve stability and biocompatibility, thereby enabling further functionalization for biomedical applications such as drug delivery and imaging.^[^ ^33^ ^]^ Zeleňáková et al., demonstrated that silica‐coated MNPs facilitate the attachment of functional groups, further enhancing stability and performance in diverse applications.^[^ ^55^ ^]^ These studies underscore the vital role of surface chemistry in broadening MNP functionality across various fields.

#### 2.3.5 MHT

The MHT properties of MNPs have garnered significant attention due to their effectiveness in cancer research. Recent advancements in cancer research have significantly focused on the application of MHT as a promising therapeutic strategy. MHT utilizes MNPs to generate localized heat when exposed to an AMF, effectively targeting and destroying cancer cells while sparing surrounding healthy tissues. This approach not only allows for the treatment of deep‐seated tumors that are otherwise difficult to access but also opens avenues for combinatorial therapies that enhance treatment efficacy. Furthermore, the integration of MHT with imaging techniques, such as MRI and photoacoustic imaging, has led to the development of theranostic tools, enabling simultaneous treatment and monitoring of tumor response. Despite its potential, the clinical application of MHT is still in its infancy, necessitating standardized protocols and further research to optimize its effectiveness across various cancer types.^[^ ^56^, ^57^, ^58^ ^]^ This method exploits the heating effect of MNPs when exposed to an AMF, relying on two primary mechanisms: Néel relaxation and Brownian relaxation.^[^ ^59^, ^60^ ^]^ In Néel relaxation, the magnetic moment of the NP rotates internally without the physical movement of the particle itself. In contrast, Brownian relaxation involves the physical rotation of the entire NP.^[^ ^61^ ^]^ The heat generated by both mechanisms can disrupt cell walls, ultimately leading to their destruction.

### 2.4 Composition of Magnetic Nanostructures

Magnetic nanostructures can be broadly classified based on their composition and structure into a few types.

#### 2.4.1 Monocomponent Magnetic Nanostructures Fe‐, Ni‐, and Co‐Based Magnetic Nanostructures

Over the past decade, nanostructures based on iron, nickel, and cobalt have attracted considerable attention due to their unique magnetic properties and diverse applications. These NPs exhibit superparamagnetic behavior and biocompatibility, making them particularly valuable in nanomedicine and other fields.^[^ ^62^, ^63^ ^]^

Iron NPs are a class of particles defined by their sizes being smaller than 40 nm, which exhibit unique optical, magnetic, and chemical properties due to their small dimensions. They have garnered significant interest in various fields, particularly in biomedical applications and magnetic data storage, where their customizable properties, such as coercivity, can be tailored for specific uses. Despite their potential, the extreme reactivity of iron NPs, including their pyrophoric nature, poses challenges for handling and application. Recent research efforts are focused on mitigating these issues, particularly through the development of effective protective coatings to enhance their stability and usability in practical applications.^[^ ^64^, ^65^, ^66^ ^]^

The magnetic properties of nickel NPs have also being explored. A recent study reported the synthesis of monodisperse 3.7 nm Ni NPs via the reduction of Ni(acac)_3_ in the presence of hexadecyl amine. These NPs hold significant potential for applications in catalysis, magnetic storage devices, and other advanced technologies.^[^ ^67^ ^]^ Similarly, cobalt NPs have been successfully synthesized in the size range of 2−11 nm using various trialkyl phosphine‐reducing agents.^[^ ^68^ ^]^ Recent studies have demonstrated that the size of Co NPs can be precisely controlled through the selection of surfactant, enabling tailored magnetic properties. These NPs show promise for applications in MHT and TDD.^[^ ^69^ ^]^

#### 2.4.2 Metal Alloys Magnetic Nanostructures

Metal alloy NPs, such as iron–platinum (FePt) and iron–palladium alloy (FePd), have garnered significant attention due to their excellent chemical stability and high magnetic crystallinity.^[^ ^70^, ^71^ ^]^ Monodisperse FePt NPs have been synthesized through the reduction of Pt(acac)_2_ combined with the decomposition of Fe(CO)_5_. Additionally, synthesis methods incorporating MgO coating have been developed to prevent agglomeration during the phase transformation from face‐centered cubic to face‐centered tetragonal structures.^[^ ^72^, ^73^ ^]^ Similarly, FePd NPs have been extensively studied, with superparamagnetic FePd NPs ranging from 11–16 nm successfully synthesized using adamantine carboxylic acid and tributyl phosphine as stabilizers.^[^ ^71^ ^]^

Beyond their structural and magnetic properties, FePt NPs have gained widespread attention for biomedical applications due to their unique characteristics and diverse functionalities. One of the most significant applications of FePt NPs is their role in enhancing contrast in MRI. Their superparamagnetic nature, combined with high chemical stability and resistance to oxidation, makes them highly effective as contrast enhancement agents, improving imaging accuracy and diagnostics capabilities in MRI.^[^ ^74^, ^75^ ^]^

#### 2.4.3 Metal Oxide Magnetic Nanostructures

Metal oxide NPs have received considerable attention in recent years due to their unique combination of magnetic properties and chemical stability. Among metal oxides, iron oxides, such as Fe_3_O_4_, have been extensively studied, particularly when synthesized through the coprecipitation of Fe^2+^ and Fe^3+^ ions in an alkaline solution. Notably, the size and morphology of these NPs can be readily tuned by adjusting parameters, such as solvent composition, surfactant presence, and reaction conditions. Surfactants, in particular, play a crucial role in directing crystalline growth by modulating the surface energies of the forming particles. Consequently, specific modifications can influence the formation of distinct Fe_3_O_4_ structures, such as octahedral NPs or nanoprism structures.^[^ ^76^, ^77^ ^]^

Beyond iron oxides, cobalt oxide NPs have also been extensively investigated. For instance, CoO nanoplatelets have been synthesized through hydrothermal methods, utilizing cobalt nitrate serving as a precursor.^[^ ^78^ ^]^ These findings underscore the versatility of metal oxide NPs in a wide range of applications, driven by their tunable structure and physicochemical properties.^[^ ^78^ ^]^

#### 2.4.4 Metal Carbides Magnetic Nanostructures

Iron carbides (Fe_5_C_2_, Fe_3_C, Fe_2_C) exhibit promising magnetic properties; however, they have been less studied due to challenges associated with controlling their size and morphology during synthesis.^[^ ^79^ ^]^ Fe_5_C_2_ NPs have been synthesized via Fe(CO)_5_ decomposition in the presence of octadecyl amine. Additionally, chemical routes for producing iron carbide NPs with distinct crystalline structures have been developed, demonstrating that the choice of synthetic approach significantly influences their resulting magnetic properties.^[^ ^79^ ^]^

#### 2.4.5 Multicomponent MNPs

##### 2.4.5.1 Heterostructure MNPs

Heterostructures that integrate magnetic properties with other materials have emerged as a promising area of research. For instance, Fe_3_O_4_@Au@Ag NPs with tunable properties have been successfully synthesized.^[^ ^80^ ^]^ Among the most versatile multifunctional materials with a wide range of applications are Fe_3_O_4_@Au@Ag NPs. These heterostructures combine magnetic properties from Fe_3_O_4_ with the plasmonic properties of Au and Ag, yielding NPs with tunable optical and magnetic characteristics.^[^ ^81^ ^]^ Therefore, the core–shell structure endows them with enhanced stability and biocompatibility, which are widely applied in biomedicine, including TDD, enhancing MRI contrast, photothermal therapy (PTT), biosensing, catalysis, environmental remediation, and as a multifunctional probe for theranostic application in cancer treatment.^[^ ^82^, ^83^, ^84^ ^]^

Additionally, FePt–Au heterostructures have been widely studied due to the combination of FePt's magnetic properties and Au's biomedical applications. The formation of FePt–Au heterostructure nanowires has been reported through the growth of Au NPs on FePt nanorods.^[^ ^85^ ^]^ In addition to FePt–Au heterostructures, antibody‐coated Au magnetic nanoshells have emerged as promising multifunctional NPs for biomedical applications. Busch et al. developed polyclonal antibody‐functionalized gold‐coated magnetic nanoshells (pAb–Lis–AuMNs) for the detection and aggregation of *Listeria monocytogenes*. These nanoshells, ≈300 nm in size, demonstrated the ability to adhere to *L. monocytogenes* and serve as surface‐enhanced Raman spectroscopy probes for biosensing and bacteria separation. The pAb–Lis–AuMNs achieved a limit of detection of ≈10^3^ CFU ml^−1^ and enabled rapid detection of *L. monocytogenes* within 5 min. Furthermore, these functionalized magnetic nanoshells enabled the capture and separation of bacteria under a low magnetic field using a permanent magnet, demonstrating their potential for the selective separation and sorting of different bacterial strains.^[^ ^54^ ^]^

##### 2.4.5.2 Exchange‐Coupled MNPs

Hard–soft magnetic phase exchange coupling has been explored as a strategy for achieving high‐energy products. The deposition of soft magnetic shells onto hard magnetic cores can enhance magnetization through effective exchange coupling.^[^ ^70^ ^]^

Nanocomposites of Nd_2_Fe_14_B and SmCo_5_ have been synthesized to achieve higher values of (BH)max.^[^ ^86^ ^]^ These exchange‐coupled nanocomposites represent a promising avenue for the development of high‐performance permanent magnets.

Ultimately, the composition of magnetic nanostructures dictates their properties and potential applications. Recent advances in synthetic methods have enabled greater control over the size, shape, and composition of magnetic nanostructures, allowing for the precise tailoring of these materials for numerous applications. Further optimization of multicomponent structures and the exploration of novel compositions might likely be key areas of future research aimed at achieving enhanced magnetic properties and functionalities.^[^ ^87^, ^88^ ^]^

### 2.5 Structure of SPIONs (Fe_3_O_4_)

Fe_3_O_4_ MNPs, or SPIONs, exhibit a unique inverse spinel crystal structure that significantly influences their magnetic properties and applications. The structural characteristics of these NPs have been extensively documented in recent literature. The inverse spinel structure of Fe_3_O_4_ consists of a cubic close‐packed arrangement of oxygen anions, with iron cations occupying both tetrahedral and octahedral interstitial sites. This configuration is conventionally represented as {Fe^3+^ A [Fe^2+^, Fe^3+^]BO_4_}, where Fe^3+^ ions are distributed between tetrahedral (A) and octahedral (B) coordination sites, while Fe^2+^ ions are confined to octahedral geometry. The interexchange between A‐O and B‐O layers facilitates crystallization into repeating magnetite lattices.^[^ ^89^, ^90^, ^91^ ^]^

Various analytical techniques have confirmed the spinel structure of Fe_3_O_4_ NPs. X‐ray diffraction (XRD) analyses have revealed diffraction peaks corresponding to the inverse spinel phase, as indexed in JCPDS card No. 075‐0449.^[^ ^92^ ^]^ Additionally, transmission electron microscopy (TEM) studies have revealed well‐defined lattice fringes that match the expected d‐spacings for magnetite, further confirming their crystalline nature.^[^ ^93^ ^]^ Notably, the structural properties of Fe_3_O_4_ MNPs can be readily modified by particle size, synthesis methodologies, and surface modifications. These variables can influence the magnetic behavior of the NPs and their potential applications in various fields. For instance, recent studies have demonstrated that the crystallite size of Fe_3_O_4_ NP crystallites can range from 8 to 12 nm, depending on the synthesis conditions and doping/composite preparation.^[^ ^92^ ^]^ The overall particle morphology is typically spherical, as observed in field‐emission scanning electron microscopy studies, which have reported grain sizes ranging from 30 to 50 nm.

**Figure 3:**
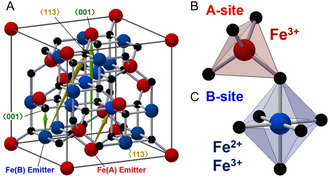
A) Magnetite has an inverse spinel structure. Arrows show the relationship between emitter atoms and scatter atoms in ⟨001⟩ and ⟨113⟩ directions. B) Fe atom occupying tetrahedral A‐site. C) Fe atom occupying octahedral B‐site. Reproduced (Adapted) with permission.^[^ ^90^ ^]^ Copyright 2019, Wiley‐VCH GmbH.

A comprehensive understanding of the structural nuances of Fe_3_O_4_ MNPs is essential for tailoring their properties for specific applications, such as MHT, drug delivery, and environmental remediation.^[^ ^94^ ^]^ In the inverse spinel structure of Fe_3_O_4_, iron ions occupy both tetrahedral (A) and octahedral (B) sites in the crystal lattice, as shown in **Figure** **3**.^[^ ^90^ ^]^

### 2.6 Advantages of SPIONs (Fe_3_O_4_)

SPIONs offer significant advantages, particularly in biomedical and environmental applications, with their superparamagnetic behavior enabling precise manipulation under external magnetic fields.^[^ ^95^ ^]^ This characteristic is particularly beneficial in applications, such as TDD, MHT, and MRI.^[^ ^40^ ^]^ Another key advantage is their high surface area‐to‐volume ratio, which enhances their catalytic activity and facilitates the efficient functionalization of various molecules on their surface.^[^ ^96^, ^97^ ^]^ Additionally, Fe_3_O_4_ MNPs exhibit biocompatibility and low toxicity, making them suitable for in vivo applications, while their chemical stability ensures long‐term effectiveness. Their natural abundance and relatively low production cost further contribute to their economic feasibility for large‐scale applications.^[^ ^95^ ^]^

Moreover, Fe_3_O_4_ MNPs possess high magnetic susceptibility and saturation magnetization, which are crucial for their performance in magnetic separation processes and as MRI contrast agents. Recently, their ecofriendliness has also been highlighted, reinforcing their potential for environmental remediation applications.^[^ ^95^ ^]^

The ability to functionalize Fe_3_O_4_ MNPs with various coatings and molecules allows their properties to be tailored for specific applications, including drug delivery, biosensing, and catalysis. They have also been used in cancer hyperthermia treatment, demonstrating efficient heating under an AMF.^[^ ^98^ ^]^ Their multifunctional nature, combining both diagnostic and therapeutic capabilities, positions Fe_3_O_4_ MNPs as valuable tools in theranostic applications.^[^ ^98^ ^]^ Furthermore, ongoing research focused on optimizing the synthesis and functionalization of Fe_3_O_4_ MNP continues to expand across diverse fields.^[^ ^99^ ^]^

### 2.7 Limitations of SPIONs (Fe_3_O_4_)

Magnetite NPs (Fe_3_O_4_ MNPs) face several limitations when used individually, restricting their broader application in various fields. One of the primary challenges is their tendency to aggregate due to strong magnetic dipole–dipole interactions, which reduces their surface area and diminishes catalytic and functional efficiency.^[^ ^100^ ^]^ Additionally, under specific conditions(oxidizing conditions and acidic environments), nonfunctionalized Fe_3_O_4_ NPs are prone to degradation and leaching, compromising their stability and raising concerns regarding biocompatibility and environmental safety.^[^ ^100^, ^101^ ^]^ Experimentally, synthesizing a monophasic and stoichiometric Fe_3_O_4_ phase has proven to be intrinsically challenging, often resulting in compositional heterogeneity or the formation of secondary phases, such as maghemite or wüstite.^[^ ^89^ ^]^ This heterogeneity can adversely affect their magnetic and catalytic properties as well as their reproducibility in applications. Furthermore, Fe_3_O_4_ NPs exhibit size and shape polydispersity, particularly during the synthesis of larger NPs, which complicates the correlation between their structural characteristics and functional performance. Another significant limitation is their susceptibility to oxidation under ambient conditions, which alters their magnetic properties and ultimately reduces their functionality over time. In biomedical applications, such as hyperthermia treatment and drug delivery, Fe_3_O_4_ NPs face additional challenges, including aggregation in biological media with high ionic strength or viscosity, which hinders their mobility and heating efficiency.^[^ ^98^ ^]^ These limitations lead to surface modifications or the development of nanocomposite materials to enhance stability and prevent aggregation, thereby improving overall performance.^[^ ^102^ ^]^ Recent research has sought to address these challenges by refining synthesis methodologies and incorporating protective coatings or functional groups, mitigating the shortcomings associated with using Fe_3_O_4_ MNPs in isolation.^[^ ^101^ ^]^

## 3 Application of MNPs in Life Sciences

MNPs have received significant interest across various fields due to their unique properties, including superparamagnetism, biocompatibility, and the ability to be controlled by an external magnetic field. **Table** **1** summarizes the significant fields in which MNPs are utilized, along with their specific applications and relevant references.

**Table 1:** Applications of MNPs in life sciences.

Fields	Applications of Magnetic NPs	References
Biomedical	‐ Drug delivery (including liposome‐coated MNPs) ‐ MHT for cancer therapy ‐ MRI and imaging contrast agents ‐ Magnetic separation and purification ‐ Controlled drug release ‐ Cellular therapy (cell labeling, tissue repair) ‐ Magnetofection ‐ Musculoskeletal disease treatment ‐ Management of inflammation and pain	[25, 35, 40, 249, 250, 251, 252, 253, 254, 255, 256, 257, 258, 259]
Healthcare	‐ Therapeutic targeting in chemotherapy (e.g., cancer) ‐ Nanoscale biosensors and imaging ‐ Nanocoatings for surfaces and implants ‐ Nanocarriers for vaccination ‐ Antimicrobial activity ‐ Nanophotothermolysis for viral infections ‐ Antigen delivery ‐ Skin ageing prevention strategies	[40, 44, 105, 253]
Agriculture and food	‐ Nanofertilizers and nanopesticides for crop yield enhancement ‐ Seed treatment ‐ Nanosensors for precision farming ‐ Encapsulation in food packaging ‐ Gene transfer for crop improvement ‐ Detection of plant diseases and pollutants ‐ Targeted delivery of agrochemicals	[228, 252, 260, 261, 262]
Environmental	‐ Removal of heavy metals and pollutants from water and soil ‐ Environmental monitoring and remediation using MNP‐based sensors	[228, 260]
Diagnostics and biosensing	‐ Immunoassays ‐ Lab‐on‐a‐chip devices ‐ Multiplexed detection of biomarkers and pathogens ‐ Protein purification ‐ Blood purification ‐ Early disease detection	[263, 264, 265, 266, 267]
Tissue engineering and regeneration	‐ Magnetic actuation for tissue formation ‐ Stem cell guidance and repair ‐ Magnetically controlled scaffolds	[268, 269, 270, 271]
Neurology	‐ Neuromodulation ‐ Deep brain stimulation ‐ Targeted delivery across the blood–brain barrier	[272, 273, 274, 275]
Micro/Nanorobotics	‐ Magnetically actuated soft robotics for biomedical interventions ‐ Minimally invasive procedures	[276,277]

### 3.1 Biomedical Application

The advent of MNPs in biomedical applications has transformed diagnostics and treatment methodologies. Due to their distinct magnetic properties, excellent biocompatibility, and ease of surface modification, MNPs have been widely utilized in areas such as MRI, drug targeting, and tumor treatment.^[^ ^44^, ^103^, ^104^ ^]^ The ability to control MNPs through external magnetic enables unprecedented precision in manipulating their behaviors with biological systems. This capability is particularly advantageous for site‐specific therapies, facilitating the targeted delivery of drugs or therapeutic agents to specific tissues while minimizing systemic side effects.^[^ ^44^ ^]^

Ongoing research continues to expand the scope of MNPs applications, with particular interest in designs that integrate both diagnosis and therapy—a field known as “theranostics.”^[^ ^44^ ^]^ These advancements have the potential to revolutionize personalized medicine by enabling treatments tailored to the specific needs of individual patients. Additionally, MNPs are being explored for their application in regenerative medicine and tissue engineering, offering new tissue repair and regeneration strategies.^[^ ^104^ ^]^

The key benefits of MNPs include their high surface‐area‐to‐volume, which enables extensive functionalization for customization in biomedical applications. This adaptability optimizes MNPs for various applications, such as cancer treatment via hyperthermia and molecular detection.^[^ ^44^ ^]^ However, several challenges remain, primarily concerning toxicity and biocompatibility. Factors such as size, shape, surface coating, concentration, duration of exposure, and chemical composition significantly influence MNP interactions with biological systems. For example, iron oxide NPs typically enter cells through endocytosis, a process that is highly dependent on the NP's size and coating.^[^ ^104^ ^]^ At high concentrations, MNPs can generate ROS, which may affect cellular health and viability.^[^ ^105^ ^]^ Critical challenges must be addressed to ensure the safe medical use of MNPs, including the prevention of adverse immune responses and the risk of NP deposition in vital organs. Thus, current research efforts focus on enhancing the long‐term stability and biodegradability of MNPs in biological environments while minimizing health risks.

**Figure 4:**
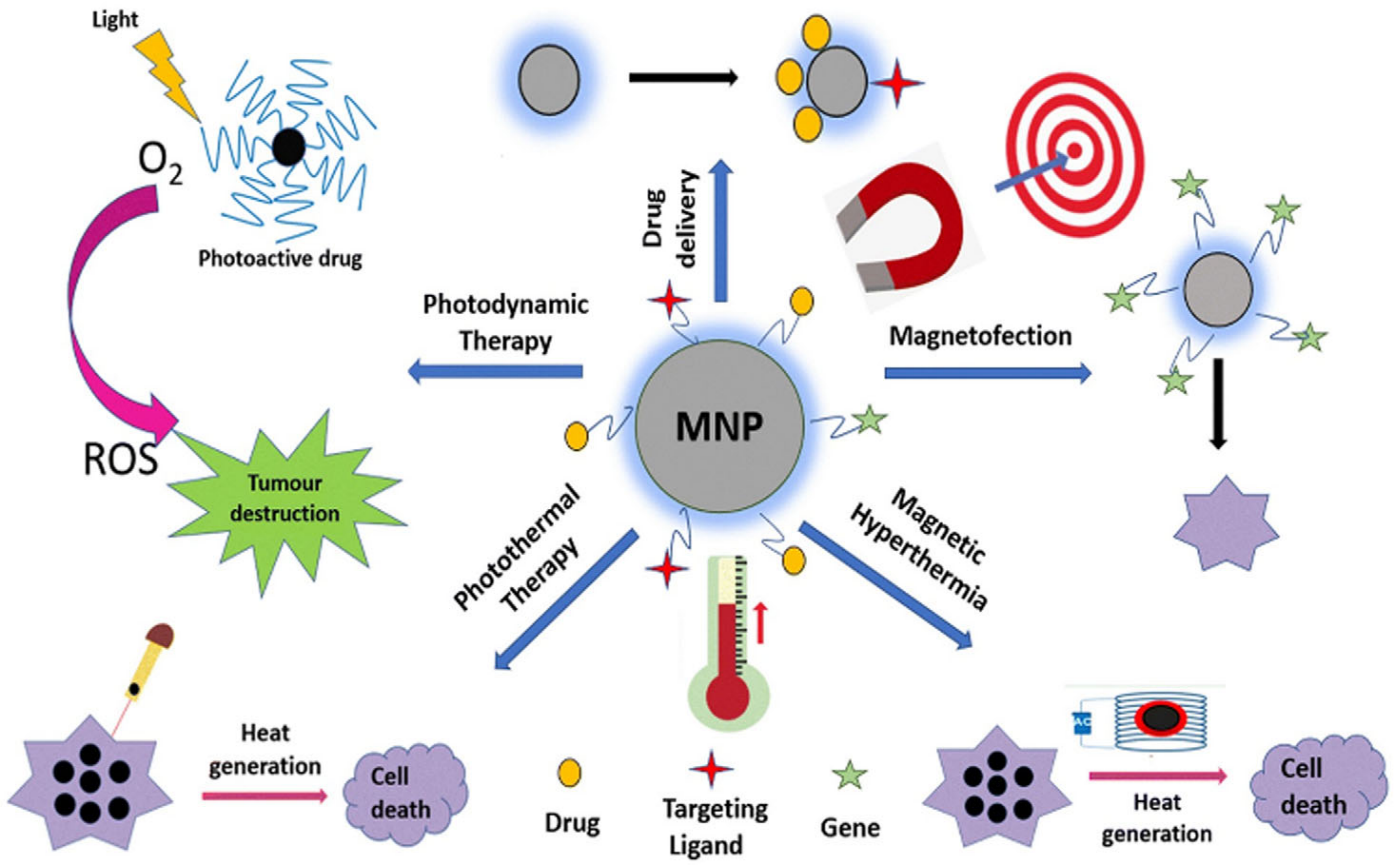
Diverse therapeutic uses of MNPs. Reproduced (Adapted) with permission.^[^ ^44^ ^]^ Copyright 2022, RSC advances.

The unique magnetic properties of MNPs serve as a key driver for their wide‐ranging biomedical applications, from diagnostic imaging to therapeutic interventions. This versatility is reflected in numerous ongoing studies and clinical developments.^[^ ^105^, ^106^ ^]^ **Figure** **4** schematically illustrates the broad spectrum of MNP‐based therapeutic applications, which will be discussed in detail, with an emphasis on recent advancements.

#### 3.1.1 MHT

Among the most promising approaches to cancer treatment, MHT utilizes MNPs in conjunction with an AMF to induce localized heating. The generated heat can raise the temperature of tumor tissues to 42–46 °C, damaging cancer cells, which are more sensitive to temperature changes than normal cells. This technique relies on the magnetic losses of MNPs, such as SPIONs, which dissipate heat under AMF exposure due to Néel and Brownian relaxation mechanisms, as explained earlier. Recent advancements have focused on improving the heating efficiency of MNPs, particularly through surface modifications. For example, sodium citrate coatings enhance colloidal dispersion and stability in biological media. Additionally, closed‐loop temperature control systems have been developed to ensure precise heating, minimizing risks to surrounding healthy tissues. MHT is often combined with other treatments, such as chemotherapy or immunotherapy, to enhance its effectiveness. However, challenges remain in optimizing NP delivery and achieving uniform heat distribution within tumors.^[^ ^107^, ^108^, ^109^, ^110^ ^]^

The effectiveness of MHT is often evaluated using the specific absorption rate (SAR), which quantifies the conversion of magnetic energy into thermal energy. SAR is calculated using the Equation (1)^[^ ^111^, ^112^ ^]^

(1) $$\text{SAR}=C\left(\frac{dT}{dt}\right)\left(\frac{m_{s}}{m_{\text{MNP}}}\right)$$

where *C* is the specific heat capacity of the solvent, *dT/dt* represents the initial slope of the time‐dependent heating curve, *m*_*s*_ is the mass of the solvent, and *m*_MNP_ is the mass of MNPs. A higher SAR value is desirable as it allows for effective treatment with a smaller quantity of MNPs and a shorter AMF exposure time.^[^ ^113^ ^]^

The effectiveness of MNPs in MH is influenced by several critical factors. Size is one of the most important parameters; the spleen and liver primarily absorb particles larger than 200 nm, while those around 10 nm are rapidly cleared from the body via the renal pathway.^[^ ^114^ ^]^ The shape also plays a crucial role, with cubic MNPs being considered optimal for MHT due to their superior SAR. The SAR efficiency generally follows this order: nanocubes > nanoflowers > nano‐octahedra > truncated MNPs > nanorods.^[^ ^108^, ^109^ ^]^ Additionally, biocompatible surface coatings help prevent agglomeration, enhance stability, and prolong circulation time, ultimately improving therapeutic outcomes.^[^ ^115^ ^]^ These factors collectively determine the design and efficiency of MNPs in MH applications.

Various types of magnetic nanomaterials have been explored for hyperthermia, including iron oxide‐based NPs, such as magnetite NPs (Fe_3_O_4_) and maghemite NPs (γ‐Fe_2_O_3_) and metallic NPs, like manganese (Mn), iron (Fe), cobalt (Co), nickel (Ni), zinc (Zn), gadolinium (Gd), and magnesium (Mg). Iron oxide‐based agents, including magnetite and maghemite, can be stabilized with ligands, such as dextran, cationic liposomes, polyvinyl alcohol, hydrogel, and lauric acid.^[^ ^114^, ^116^, ^117^, ^118^, ^119^, ^120^ ^]^

Additionally, various ferrites have been investigated, including simple ferrites, such as cobalt ferrite CoFe_2_O_4_, manganese ferrite MnFe_2_O_4_, nickel ferrite NiFe_2_O_4_, lithium ferrite Li_0_._5_Fe_2_._5_O_4_, mixed ferrites, such as nickel‐zinc–copper ferrite Ni_0_._65_Zn_0_._35_Cu_0_._1_Fe_1_._9_O_4_, and cobalt–nickel ferrite CoxNi(1−x)Fe_2_O_4_. Other ferromagnetic NPs, such as Fe‐doped gold (Au), Zn–Mn‐doped iron oxides ZnxMn(1−x)Fe_3_O_4_, and Mn–Zn–Gd‐doped iron oxide composites Mn_x_Zn_x_Gd_x_Fe_(2−x)_O_4_ have also been investigated. Notably, FeCo metallic NPs have demonstrated excellent heating performance, with SAR values ranging from 1300 to 1600 W g^−1^.^[^ ^114^, ^115^, ^116^, ^117^, ^118^, ^119^, ^121^, ^122^ ^]^

Iron oxide‐based MNPs remain highly favored for biomedical applications due to their biocompatibility and low toxicity, as they participate in metabolic pathways that regulate iron homeostasis. Magnetite (Fe_3_O_4_) offers several advantages over cobalt‐based NPs, including higher Curie temperatures, greater saturation magnetization (≈90–98 emu g^−1^), and lower toxicity in preclinical studies. Clinical studies, such as those investigating thermo radiotherapy for prostate cancer using Co–Pd thermoseeds, have successfully achieved intraprostatic temperatures of 42–46 °C without inducing toxicity. Theoretical and experimental research highlights the crucial role of key parameters in ensuring efficient hyperthermia, including particle size and size distribution, magnetic properties (magnetic moment and magnetic anisotropy), and viscosity of the dispersion medium. These parameters optimize MNPs, as discussed in the following sections.^[^ ^123^, ^124^ ^]^

MH leverages MNPs’ ability to generate localized heat (41–47 °C) under AMFs, enabling selective tumor cell targeting while sparing healthy tissue. MH therapy offers advantages over chemotherapy, including greater specificity and reduced damage to normal cells, and has been in practice since the 1950s. Recent advancements have further enhanced the precision and efficacy of MH therapy.^[^ ^125^, ^126^, ^127^ ^]^

For example, Angelakeris et al. (2023) synthesized magnetite NPs (16–76 nm) via pH‐tuned aqueous precipitation and evaluated their structural and magnetic properties under varying medium compositions, MNP concentrations, and applied magnetic field parameters. The study found that heating efficiency was optimal for specific particle sizes and field conditions, with heat generation attributed to Brownian, Néel, and hysteresis losses. Additionally, clinical studies using agarose‐immobilized MNPs identified optimal conditions for low‐field MH therapy applications, further refining treatment precision.^[^ ^128^ ^]^

Recent studies have explored Fe_3_O_4_/Halloysite nanotube composites synthesized via coprecipitation techniques, with NP sizes ranging from 30 to 70 nm. The saturation magnetizations for Fe_3_O_4_ and its composite were reported as 73.84 and 30.63 emu g^−1^, respectively. The maximum SAR values were recorded at 94 W g^−1^ at 400 kHz and 53 W g^−1^ at 200 kHz, indicating their potential as promising candidates for effective hyperthermia treatment [305]. Additionally, Fe_3_O_4_ MNPs alone exhibited a SAR value of 72.42 W g^−1^, highlighting their capability for selective tumor heating with minimal damage to surrounding healthy cells.^[^ ^129^ ^]^

New xanthan hydrogels and silk fibroin matrices incorporating Fe_3_O_4_ colloids have demonstrated high SAR values even at very low concentrations (7 W g^−1^ at 1 mg mL^−1^), supporting their potential for localized hyperthermia treatment. However, further studies are required to assess long‐term stability, in vivo biocompatibility, and optimal magnetic field conditions for clinical applications.^[^ ^130^ ^]^

Recent work by Seal et al. (2025) highlighted magnetite composites with multiwalled carbon nanotubes (MWCNTs), demonstrating that optimized MWCNT concentrations enhanced SAR values through Néel relaxation mechanisms.^[^ ^118^ ^]^ Other newly developed composites, such as iron oxide–gold hybrid and cross‐linked pectin–cellulose hydrogels, have shown improved heating properties and/or biocompatibility, offering promising avenues for new cancer therapies.^[^ ^128^, ^129^, ^131^ ^]^ A recent study reported the development of MnZn‐SPION‐7, a doped superparamagnetic NP engineered for enhanced performance in MHT and MRI. The investigation provided a comprehensive characterization of the NPs’ magnetic and physicochemical properties, along with thorough assessments of their biocompatibility, colloidal stability, and theranostic efficacy in both in vitro and in vivo models. Comparative analyses with conventional SPIONs underscored the advantages of the MnZn doping strategy, offering valuable insights into toxicity profiles and functional performance relevant to clinical translation.^[^ ^132^ ^]^

Surface coatings play an important role in enhancing MNP performance. For example, Fe_3_O_4_ NPs coated with sodium citrate exhibited an SLP of ≈170 W g^−1^ under specific field conditions, demonstrating improved stability and reduced aggregation.^[^ ^133^ ^]^ These studies emphasize the necessity of biocompatible surface functionalization to achieve higher therapeutic efficacy.^[^ ^109^ ^]^ Optimizing NP size, surface functionalization, and magnetic field parameters remains critical for advancing MH therapy. Novel strategies under investigation include mitochondrially targeted MNPs and biosynthetically fabricated MNPs inside the cancer cells, both of which have shown promising improvements in treatment performance.^[^ ^130^, ^134^, ^135^, ^136^, ^137^, ^138^ ^]^ A study investigated the impact of three biocompatible coatings—sodium citrate, (3‐aminopropyl)triethoxysilane (APTES), and dextran—on SPIONs. The study demonstrated that these coatings effectively stabilized the NPs, influenced their clustering behavior, and altered their magnetic properties. It also examined oxidation kinetics and long‐term stability, providing valuable insights for optimizing functionalization strategies in biomedical applications.^[^ ^139^ ^]^ The study provided direct evidence that surface functionalization (sodium citrate, APTES, or dextran) not only improves colloidal stability but also tailors the magnetic and structural properties of SPIONs. This customization is critical for their performance in drug delivery, imaging, and hyperthermia.^[^ ^139^ ^]^

Yuling et al. also discussed the use of polydopamine and other coatings to enhance cellular targeting, modulate immune responses, and improve therapeutic outcomes. Their research emphasized the upregulation of key chemokine receptors for better cell homing.^[^ ^140^ ^]^

Additionally, recent research has significantly advanced our understanding of MNPs, particularly SPIONs and iron‐based functionalized MNPs, with a strong focus on toxicity assessment and the influence of various surface coatings. For instance, Chapa González et al. systematically investigated chitosan‐coated magnetite NPs, demonstrating that synthesis conditions, especially pH, profoundly affect particle size, surface charge, coating mass, and magnetic properties. All of these factors are crucial for optimizing biomedical safety and efficacy. The work also highlighted that appropriate surface functionalization, such as chitosan coating, can enhance colloidal stability and biocompatibility, directly impacting biodistribution and pharmacokinetics in vivo.^[^ ^141^ ^]^ Despite significant advancements, key challenges remain in translating experimental nanomaterial research into clinical applications, particularly concerning long‐term safety, reproducibility, and biocompatibility. Critical issues, such as controlling synthesis and functionalization, ensuring effective cancer cell targeting, and addressing potential toxicity, must be addressed to facilitate broader clinical adoption.^[^ ^142^, ^143^ ^]^ In a separate study, researchers loaded monocytic THP‐1 cells with citrate‐coated and gold‐coated SPIONs. They found that both coatings facilitated efficient cellular uptake, demonstrated excellent biocompatibility, and produced minimal ROS. Significantly, the coated SPIONs enabled effective magnetic steering of the cells, and computational models accurately predicted the magnetic enrichment. This study is particularly relevant for applications in immunotherapy and targeted cell delivery.^[^ ^144^ ^]^ Considerable emphasis has been placed on optimizing material properties, particularly SAR and biocompatibility, as they are essential for enhancing therapeutic efficacy while minimizing toxicity. These findings highlight the promising potential of innovative nanocomposite systems, especially those derived from natural polymers through greener synthesis methods, for applications in drug delivery, imaging, and cancer therapy. This progress paves the way for more targeted and efficient nanomedicine approaches, offering exciting prospects for the future of personalized disease treatment. Another recent study combined traditional toxicological methods with proteomics to assess the toxicity of intravenously administered IONPs in rats. The results showed that high doses caused mild toxic effects and significant molecular changes, particularly in the spleen. The study identified the AKT/mTOR/TFEB pathway as being involved in autophagy and lysosomal activation. This thorough mechanistic investigation enhances our understanding of IONP toxicity and emphasize the importance of multiomics approaches in safety assessment.^[^ ^145^ ^]^ Additionally, Modi et al. also rigorously evaluated the biocompatibility and ROS generation of various SPION coatings, confirming their safety for biomedical applications.^[^ ^144^ ^]^

Despite significant advances, several key challenges remain in the biomedical application of nanomaterials. These include NP biocompatibility and toxicity, challenges in synthesis and functionalization control, and effective targeting of cancerous cells. Additionally, the heterogeneous responses of cancer cells, such as radioresistance and drug resistance in certain cell populations, present major obstacles to improving therapeutic outcomes. Future research must focus on translating nanocomposites into multifunctional systems that offer enhanced therapeutic performance with minimal or negligible side effects. There is also an imminent need to further investigate NP interactions in complex biological systems, ensuring their safety and efficacy. Furthermore, adopting green synthesis approaches will be crucial for developing nanomaterials that are both less harmful and highly effective, paving the way for safer and more sustainable biomedical applications.

The clearance of MNPs by the liver, spleen, kidneys, and bone marrow is influenced by several critical factors, including particle size, shape, and surface charge. Particles larger than 100 nm are rapidly cleared, and smaller particles have a more significant surface‐to‐volume ratio, hence a better adhesion of proteins. On the contrary, such adhesion enhances recognition through macrophages, being subsequently cleared by the liver and spleen. Additionally, the surface charge of MNPs strongly affects their circulation and residence times, as well as their heating efficiency. A high surface charge is typically associated with increased plasma protein adsorption, resulting in faster sequestration by macrophages and shorter circulation times in the bloodstream.

Current magnetic NP‐based hyperthermia strategies aim to maximize therapeutic efficacy while minimizing side effects. These advances are driving MHT closer to clinical application, both as a standalone treatment and in combination with other therapeutic modalities. Recent research has also explored the magnetization of natural and synthetic polymers by incorporating ferrite oxide NPs, enhancing their susceptibility to hyperthermia. Key parameters, such as NP type, concentration, SAR value, and the saturation magnetization of magnetic cross‐linked nanobiocomposites, are summarized in Table S1, S2, Supporting Information.

MHT has gained significant interest as a rapidly advancing cancer treatment modality. This approach utilizes superparamagnetic NPs that generate heat within tumor tissues upon exposure to an AMF, typically raising the temperature to 42–46 °C. This temperature range is considered sufficient to induce apoptosis in cancerous cells, enabling a targeted tumor ablation strategy.^[^ ^146^, ^147^ ^]^ The efficacy of MHT depends on several factors, including NP size and shape, magnetic properties, and the intensity and frequency of the applied AMF. Heat generation primarily occurs through magnetic hysteresis losses, which are particularly effective in viscous media. Recent progress has led to the development of multifunctional nanocomposites with both therapeutic and diagnostic capabilities, offering enhanced treatment outcomes. Additionally, biocompatible coatings have been introduced to improve colloidal stability and heating efficiency. However, several challenges remain, including the potential for nonspecific heating, which could inadvertently damage healthy tissues, and the development of improved targeting mechanisms to ensure NP accumulation at tumor sites.^[^ ^146^, ^148^ ^]^ Further optimization of NP magnetic properties, stability, and delivery systems is necessary to minimize potential damage to surrounding tissues. Despite these challenges, MHT remains a promising noninvasive treatment for cancer. Active research continues to fully harness its clinical potential.^[^ ^149^ ^]^

MHT is an innovative and effective therapeutic strategy that uses superparamagnetic NPs, primarily composed of iron oxide (Fe_3_O_4_). The treatment generates heat in tumor tissue when exposed to an AMF. Several key factors influence this process, including the size, shape, and magnetic properties of the NPs, as well as the intensity and frequency of the applied magnetic field. Research has shown that the heating efficiency, quantified as the SAR, is significantly influenced by these variables and tends to be higher for larger NPs.^[^ ^150^, ^151^ ^]^ Recent advancements in this field have led to the development of mechanically enhanced, multifunctional nanocomposites with improved performance.

Further developments in NP synthesis have explored methods, such as hydrothermal techniques, coprecipitation techniques, and greener approaches that utilize plant extracts. These methods have been designed to enhance the properties of MNPs, with a focus on their magnetic characteristics and biocompatibility. Advanced characterization techniques, including XRD, TEM, and thermogravimetric analysis, have been employed to confirm the structural integrity and thermal stability of the nanocomposites. However, several challenges remain unresolved. For instance, nonselective heating may damage healthy tissues surrounding tumors. Additionally, effectively targeting NPs at tumor sites remains a significant challenge. The scalability of NP synthesis, while maintaining quality and uniformity, also presents a significant challenge for clinical applications.

Future research should focus on refining synthesis methodologies to enhance the magnetic properties of NPs and improve their stability, as well as discovering new materials with improved performance and developing effective delivery systems for precise targeting. Addressing these challenges is crucial for advancing MHT as a noninvasive cancer therapy. Although MHT shows great promise, substantial further research and engineering are required to overcome existing obstacles and fully leverage this technique for effective tumor treatment.

#### 3.1.2 Photodynamic Therapy (PDT)

PDT is a rapidly developing treatment modality that uses light‐activated photosensitizers to induce cytotoxic effects in targeted tissues, offering a novel approach to cancer treatment. This technique involves administering a photosensitizing agent, which is activated by light of a specific wavelength. The combined effect of the photosensitizer and the light generates ROS, leading to cell death through pathways such as apoptosis and necrosis. Recent advancements in nanotechnology, particularly with the application of MNPs, have significantly enhanced the efficacy and specificity of PDT.^[^ ^152^ ^]^

MNPs are particularly advantageous in PDT due to their unique properties, such as superparamagnetism, which enables the manipulation of these nanomaterials using external magnetic fields. This capability not only facilitates the targeted delivery of therapeutic agents but also enhances the photodynamic effect when exposed to light. For example, silica‐coated Fe_3_O_4_ MNPs loaded with curcumin, a natural photosensitizer, have shown effective results in vivo for treating breast cancer. When irradiated with appropriate laser wavelengths, these NPs produce both hyperthermia and singlet oxygen, significantly improving the overall therapeutic outcomes compared to conventional PDT alone.^[^ ^153^ ^]^

Integrating MNPs in PDT protocols has led to the development of multifunctional nanocarriers that can carry drugs while providing enhanced imaging capabilities. A dual‐functioning magnetic NP conjugated with carbon quantum dots has been synthesized to combine hyperthermia with PDT. This approach enhances therapeutic efficacy and minimizes the side effects associated with conventional cancer treatments.^[^ ^154^ ^]^

Additionally, functionalizing MNPs to improve their biocompatibility and targeting capabilities represents another significant advancement in PDT. By modifying the surface of these NPs with biocompatible materials, such as silica or polymers, researchers can effectively conjugate photosensitizers with targeting ligands. The strategy enhances the accumulation of therapeutic agents in tumor tissues while reducing systemic toxicity. Recent studies have demonstrated the effectiveness of these modified NPs in achieving higher fluorescence intensity and improved photodynamic effects in cancer cells.^[^ ^152^ ^]^

Particle size, shape, and magnetic properties play important roles in optimizing MNPs’ performance for PDT applications. Smaller particles can penetrate more deeply into tissues and accumulate more in tumors, while larger particles enhance magnetic targeting.^[^ ^44^, ^155^ ^]^

Combining PDT with MHT represents one of the most promising advancements in cancer treatment. This synergistic approach leverages the unique properties of MNPs to enhance the effectiveness of both therapies. In PDT, photosensitizers are activated by light, leading to the generation of ROS that induce cell death. In contrast, MHT involves heating MNPs through an AMF. For instance, the heat produced by MHT increases blood flow and oxygenation in the tumor, thereby enhancing the efficacy of PDT, which depends on oxygen to produce reactive species.^[^ ^156^ ^]^ Additionally, targeting is improved, as external magnetic fields can be used to guide MNPs directly to the tumor site, ensuring more precise delivery of both the photosensitizer and the hyperthermia effect.^[^ ^154^ ^]^ The combination of PDT with MHT has demonstrated strong synergistic antitumor activity, which induces a tumor‐specific immune response and may help combat primary tumors as well as metastasis.

**Figure 5:**
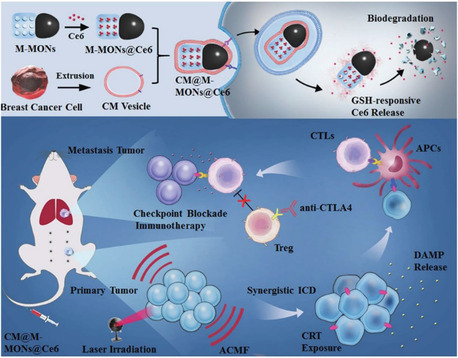
Schematic representation of the synthesis of cancer cell membrane‐coated Janus magnetic mesoporous organosilica NPs loaded with Ce6 for combined PDT and MHT with enhanced CTLA‐4 blockade therapy. This novel strategy is directed toward promoting synergistic antitumor immunity as a potent approach against cancer metastasis. Reproduced (Adapted) with permission.^[^ ^156^ ^]^ Copyright 2019, Wiley‐VCH GmbH.

The combination of PDT with MHT can help overcome some limitations associated with PDT alone, particularly its reduced effectiveness in oxygen‐deficient tumor environments.^[^ ^157^ ^]^ Wang et al. demonstrated the synergistic potential of MHT when used in conjunction with PDT to induce an antitumor immune response. This approach involved bullet‐shaped organosilica‐modified MNPs loaded with the photosensitizer chlorin e6 (Ce6) for the treatment of breast cancer cells. The NPs displayed prolonged circulation in the bloodstream and targeted tumors uniformly. By integrating PDT and MHT, an enhanced antitumor efficiency was observed, especially against tumor metastasis, as shown in **Figure** **5**.^[^ ^44^, ^156^ ^]^

#### 3.1.3 PTT

**Figure 6:**
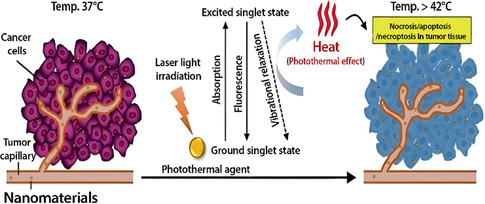
Nanomaterial‐mediated PTT is based on accumulation within a solid tumor via the enhanced permeability and retention effect due to leaky tumor vasculature. In such processes, nanomaterials with strong NIR absorbance may effectively convert laser energy into heat. This act is induced because of photothermal effects from generated heat >42 °C in the vibrational relaxation process of excited PTT agents, causing ablation of a tumor through necrosis, apoptosis, and necroptosis of tumor tissue. Reproduced (Adapted) with permission.^[^ ^158^ ^]^ Copyright 2021, MDPI.

Among the newest and least invasive cancer treatment methods is PTT, which utilizes light‐absorbing agents to induce localized heating necessary for tumor ablation. This technique has undergone extensive exploration in the last decade due to its potential for effective and precise cancer treatment with minimal side effects.^[^ ^158^ ^]^ The light absorption of photothermal agents in a specific wavelength enhances heating in target cells or tissues. After irradiation, the agents are excited from the ground singlet state to the excited singlet state. Then, via vibrational relaxation, energy is released nonradiatively to return to the ground state through collisions with surrounding molecules. This increases kinetic energy in the environment, therefore serving to heat the tumor microenvironment (**Figure** **6**).^[^ ^158^ ^]^

The general principle of PTT involves a two‐step process. First, photothermal agents are administered and then light in the near‐infrared (NIR) spectrum is applied. These agents absorb light energy and convert it into heat by relaxing nonradiatively, thereby increasing the localized temperature within the tumor microenvironment. When the temperature rises between 43 and 55 °C, it promotes cellular damage, leading to destruction through various mechanisms, including protein denaturation, disruption of the cellular membrane, and vascular damage.^[^ ^159^ ^]^

MNPs have emerged as promising photothermal agents due to their unique physicochemical properties and multifunctional capabilities. NPs, particularly those based on iron oxide, offer several advantages for PTT applications. They are highly biocompatible, easily functionalized for targeted delivery, and possess inherent magnetic properties that facilitate MRI and magnetic field‐guided delivery.^[^ ^160^ ^]^

Recent studies have demonstrated that MNPs are effective in PTT applications. For instance, the functionalized MNPs developed for hyperthermia can achieve a temperature of 54.1 °C when irradiated with a 793 nm laser, exhibiting a photothermal conversion efficiency of 35.21%.^[^ ^161^ ^]^ This high efficiency enables tumor ablation at relatively low laser power densities, thereby minimizing damage to surrounding healthy tissues.

It is anticipated that integrating PTT with other therapeutic modalities will improve treatment outcomes. For example, combining PTT with MHT—using an AMF to generate heat—has shown synergistic effects in cancer treatment.^[^ ^162^ ^]^ This dual approach harnesses the strengths of both techniques, potentially overcoming limitations such as the restricted penetration depth of light in PTT.

Additionally, MNPs facilitate the integration of diagnostic and therapeutic functions within a single platform, a concept referred to as the theranostic principle. By coupling MRI capabilities with PTT, these NPs enable real‐time localization of tumors and monitoring of therapeutic efficacy, thereby supporting more precise and personalized treatment strategies.^[^ ^163^, ^164^ ^]^

Recent advancements in the development of multifunctional nanomaterials have significantly enhanced both PDT and PTT. Due to the EPR effect, NPs ranging from 10 to 200 nm in diameter can selectively accumulate in tumor tissues, enhancing the therapeutic effect of both PDT and PTT.

Photosensitizers can be integrated into these NPs to improve fluorescence, generate ROS, and minimize unwanted side effects by releasing the photosensitizer directly at the tumor site. Additionally, NPs serve as external photo absorbers for PTT, which helps reduce the amount of external energy needed to destroy tumors while protecting the surrounding healthy tissues. By adjusting the size, shape, and surface chemistry or targeting ligands of NPs, tumor targeting can be improved. Both organic and inorganic NPs are currently being explored for delivering photoactive agents.^[^ ^159^ ^]^

Espinosa et al. have shown that iron oxide NPs can effectively function as both magnetic and photothermal agents. The authors fabricated iron oxide nanocubes, demonstrating improved heating efficiency when subjected to AMF and NIR laser irradiation. In vivo experiments with mice bearing subcutaneous tumors revealed that combining MHT with PTT significantly enhanced the therapeutic effect compared to either treatment alone. This synergy resulted in complete tumor regression and improved survival rates for the treated mice.^[^ ^165^ ^]^

The use of MNPs in PTT has opened new avenues for addressing the challenges associated with cancer treatment. For example, Fe_5_C_2_ NPs have demonstrated significant potential due to their magneto‐photothermal properties, which are particularly beneficial for hyperthermia treatment in cancer therapy.^[^ ^166^ ^]^ These NPs can respond to magnetic fields and/or NIR light.

However, despite the promising developments, several challenges remain to be addressed before clinical application can be realized. Key issues include optimizing NP design to improve tumor accumulation, enhancing photothermal conversion efficiency, and addressing long‐term toxicity concerns.^[^ ^158^ ^]^ Additionally, there is an urgent need for the standardization of treatment protocols and the development of real‐time monitoring techniques for clinical use.

#### 3.1.4 TDD

TDD aims to increase the efficiency of drugs while assuring minimum systemic toxicity. TDD involves administering therapeutics at high concentrations at the site of action, ultimately enhancing both pharmacodynamic and pharmacokinetic properties compared to traditional methods. Recent studies highlight the growing need for TDD techniques in response to the poor outcomes associated with conventional approaches including toxic side effects.^[^ ^167^, ^168^ ^]^ Nanotechnology‐based TDD has introduced innovative carrier designs that can effectively navigate biological barriers to deliver drugs precisely where needed. For example, the use of NPs allows for controlled release mechanisms that can be stimulated by external factors, providing a dynamic response tailored to the treatment's requirements.^[^ ^169^, ^170^ ^]^

MNPs are a promising tool for TDD due to their unique magnetic properties, which allow for precise control of their movements within the body using external magnetic fields. This capability enhances the localization of drugs at disease sites, such as tumors, while significantly reducing off‐target effects, thereby improving overall therapeutic efficacy.^[^ ^171^ ^]^

MNPs can be functionalized with various therapeutic agents, such as chemotherapeutics, proteins, and nucleic acids, enabling their release in response to environmental triggers or magnetic stimuli.^[^ ^172^ ^]^ Research has shown that this targeted approach effectively targets cancerous cells while minimizing toxicity to normal tissues.^[^ ^167^ ^]^ Additionally, combining MNPs with imaging techniques contributes to theranostics, a field that merges therapy and diagnostics.^[^ ^171^ ^]^

The effectiveness of MNP‐based drug delivery systems is influenced by several factors, including size, crystal structure, shape, surface chemistry, and magnetization. Recent studies have focused on optimizing these properties to enhance the efficacy of TDD.^[^ ^9^ ^]^ Multifunctional nanocarriers capable of coloading multiple drugs and therapeutic modalities have been designed for synergistic oncotherapy, integrating targeting molecules with delivery vehicles like liposomes, polymers, and silica.^[^ ^173^ ^]^

Pioneering approaches in magnetically TDD involve encapsulating MNPs within medicinal molecules for precise delivery, particularly to complex sites, such as blocked arteries. This strategy can reduce the required dosage and minimize systemic side effects.^[^ ^174^ ^]^ However, despite their potential, TDD systems face challenges related to the complexity of tumor structure, biosafety, and rapid drug clearance. Additionally, several regulatory and scalability issues need to be addressed before these systems can be successfully translated from the laboratory to clinical applications.^[^ ^175^ ^]^

**Figure 7:**
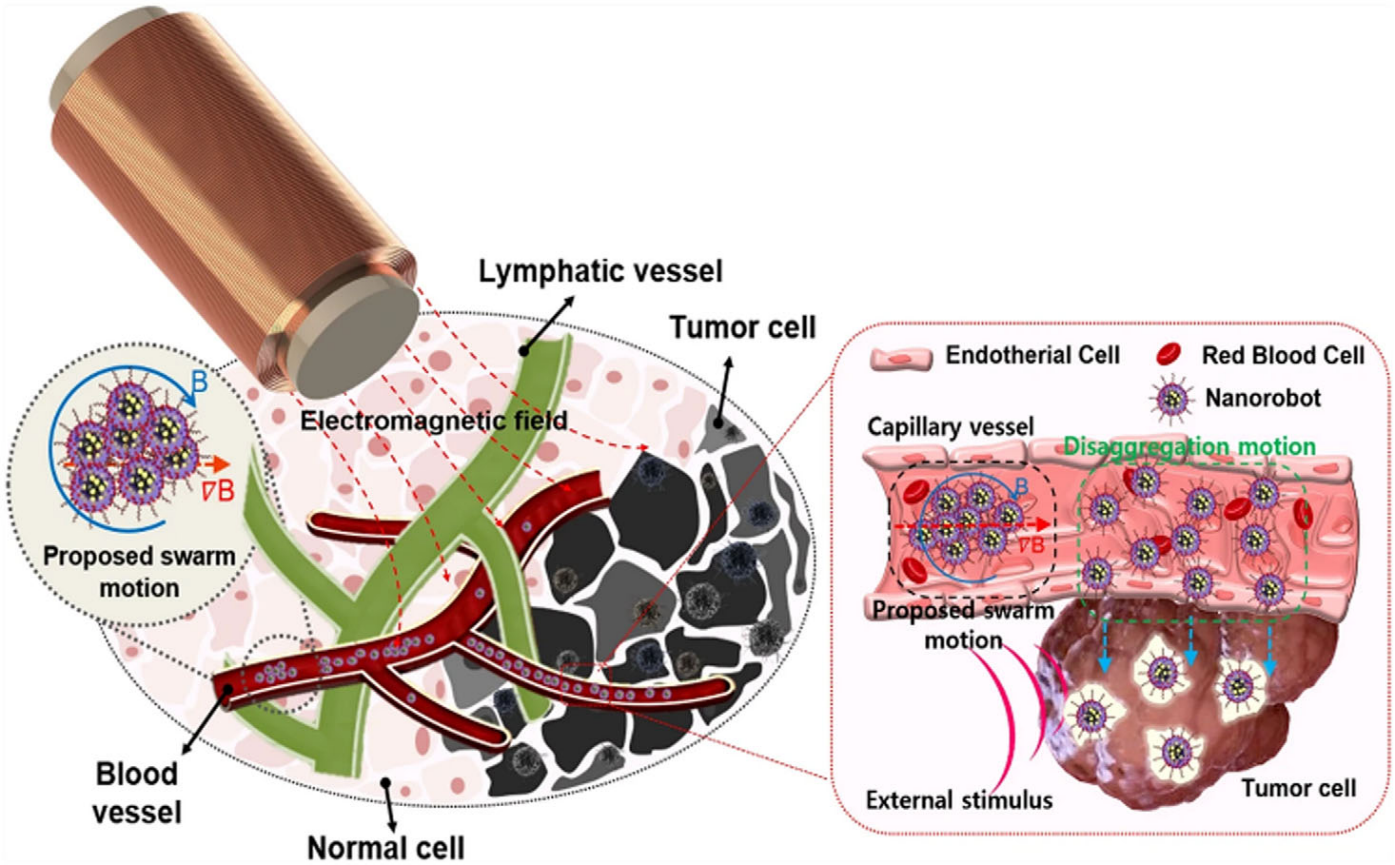
The concept of TDD using nanorobots. Guided by EnEMAs and visualized through X‐ray imaging, the nanorobots move in a coordinated swarm toward the target site. Once there, enhanced tumbling motion helps steer magnetic clusters precisely to the area of interest. These clusters then break apart into smaller particles to navigate through tiny blood vessels. Finally, upon reaching the target, an external stimulus triggers the release of the drug. Reproduced (Adapted) with permission.^[^ ^176^ ^]^ Copyright 2021, Nature (scientific report).

Recent research by Nguyen et al. (2021) focuses on the development of MNPs for TDD. The study utilized an electromagnetic actuation system to enhance the locomotion and disaggregation of these NPs, thereby improving their therapeutic efficiency. **Figure** **7** illustrates a conceptual diagram of this TDD approach, which employs paramagnetic NPs as therapeutic agents. In this system, nanorobots are guided to specific target areas using the ennead electromagnets actuation system (EnEMAs). This guidance follows a proposed swarm motion that is monitored through X‐ray imaging.

The enhanced tumbling motion of the magnetic clusters facilitates their navigation toward the target site. At the same time, the ability to disaggregate allows these clusters to break down into smaller particles that can enter the microvessels at the targeted location. Once the nanorobots reach the area of interest, an external stimulus triggers the release of the drug, enabling precise delivery of therapeutic agents to the desired site.^[^ ^176^ ^]^

TDD represents a significant shift in medicine toward more effective therapies with fewer side effects. As research progresses, these systems could enhance patient outcomes and transform treatments, especially in oncology and cardiovascular medicine. However, several challenges must be addressed before they can be successfully applied in clinical settings.

#### 3.1.5 Antimicrobial/Antibacterial Activity

Antimicrobial resistance has become an escalating health crisis recently. If current trends continue without any intervention, it is predicted that by 2050, there will be 10 million deaths annually due to this issue. The COVID‐19 pandemic has further exacerbated the problem, as the increased and often inappropriate use of antibiotics has accelerated the global spread of multidrug‐resistant bacterial strains. This resistance has rendered many traditional antibiotics less effective, making even the treatment of simple infections increasingly difficult. The limited development of new antibiotics reinforces the urgent need to explore alternative antimicrobial strategies.^[^ ^177^ ^]^

MNPs, particularly Fe_3_O_4_ NPs, have emerged as promising candidates for combating bacterial infections due to their remarkable physicochemical properties. They generate ROS, disrupt bacterial membranes, and enhance antibacterial efficacy through MHT, making them strong alternatives to conventional antibiotics.^[^ ^178^, ^179^ ^]^ Additionally, MNPs can be functionalized with antimicrobial agents or combined with other therapeutic modalities to effectively target resistant bacterial strains.^[^ ^180^ ^]^ For example, biosynthesized MNPs can significantly reduce bacteria viability without the need for antibiotics when an AMF is applied.^[^ ^177^ ^]^ The latest advancements in the mechanisms of action of MNPs, their efficacy against various bacterial strains, and the challenges associated with MNP‐based therapies are discussed next.

##### 3.1.5.1 Mechanism of Antibacterial Activities

**Figure 8:**
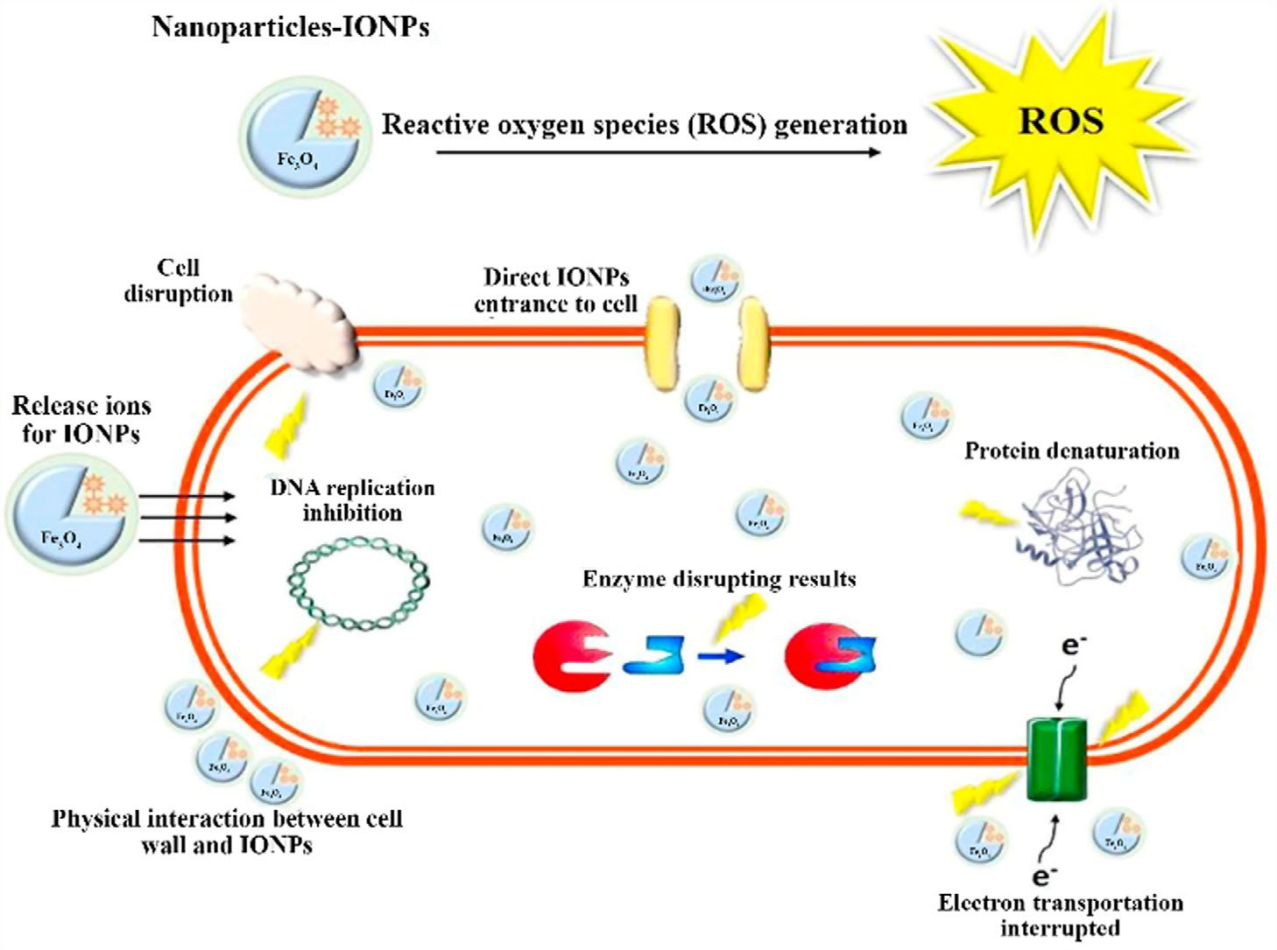
Antimicrobial mechanisms of iron oxide NPs—most importantly, magnetite—are related to the relationship between NPs and ROS in general. Reproduced (Adapted) with permission.^[^ ^247^ ^]^ Copyright 2019, Elsevier B.V.

In recent years, studying the antimicrobial activity of IONPs or MNPs has been a significant area of interest due to their potential application in combating bacterial infections. Their mechanism of action primarily involves inducing oxidative stress and physically interacting with bacterial cells. This process is explained step‐by‐step, as shown in **Figure** **8**.

###### Induction of ROS

One of the primary mechanisms by which IONPs exert their antimicrobial effects is through the generation of ROS. IONPs can induce oxidative stress by producing ROS, such as superoxide and hydrogen peroxide radicals, when they interact with bacterial cells. This process ultimately leads to cell death by damaging cellular components, including lipids, proteins, and nucleic acids. Various studies have indicated that positively charged IONPs produce significantly higher levels of ROS compared to negatively charged ones, which correlates with their increased antimicrobial efficacy.^[^ ^177^, ^178^ ^]^

###### Electrostatic and Van der Waals Interactions

IONPs can adhere to bacterial cell walls through electrostatic interactions, particularly when the bacterial membranes are predominantly negatively charged. Positively charged IONPs may aggregate on the surface of the bacterium, compromising the integrity of the cell wall and membrane, and leading to the leakage of cellular contents due to cell lysis.^[^ ^178^, ^179^ ^]^

###### Membrane Disruption

IONPs, upon attaching to the bacterial membrane, disrupt its integrity through multiple mechanisms. They can penetrate into the cytoplasm leading to the formation of vacuoles that interfere with cellular functions. Additionally, IONPs can disrupt ATP synthesis by impairing the activity of ATPase, which is essential for maintaining membrane potential and energy production in bacteria.^[^ ^181^, ^182^ ^]^

###### Enzyme Denaturation

The presence of IONPs in bacterial cells causes the denaturation of essential enzymes. This denaturation affects the enzymes, which are important for metabolic processes related to the growth and replication of bacteria.^[^ ^182^ ^]^ Consequently, the malfunctioning of these enzymes contributes to the antimicrobial effects of the NPs.

###### Enhanced Delivery Systems

MNPs can be modified with antibiotics or therapeutic agents to improve their delivery and targeting. This modification allows for the controlled release of antimicrobial agents directly at the site of infection, resulting in better therapeutic outcomes and reduced side effects.^[^ ^179^, ^183^ ^]^

The multifunctional action of IONPs and MNPs—through both physical disruption and oxidative stress—underscores their potential as effective antibacterial agents. Advancing their clinical application, however, will require further optimization to balance efficacy with minimized toxicity, especially in targeting resistant pathogens.^[^ ^184^ ^]^

###### Methods for Conducting Antibacterial Test

MNPs have emerged as promising antibacterial agents due to their unique physicochemical properties and responsiveness to external magnetic fields. Several methods are commonly used to evaluate their antibacterial efficacy. The agar well diffusion method involves creating wells in Mueller–Hinton agar plates inoculated with the target bacterium, into which MNP suspensions are introduced. The antibacterial effectiveness is then assessed by measuring the zone of inhibition surrounding the wells.^[^ ^185^ ^]^ A similar approach, the disk diffusion method, replaces wells with paper disks infused with MNPs, making it particularly useful for studying synergistic interactions between MNPs and antibiotics.^[^ ^186^ ^]^ The broth dilution method determines the minimum inhibitory concentration (MIC) and minimum bactericidal concentration of MNPs by preparing serial dilutions in a liquid growth medium containing test bacteria.^[^ ^187^ ^]^ The time‐kill assay provides insights into the bactericidal activity of MNPs over time by quantifying bacterial viability at various concentrations.^[^ ^187^ ^]^ Since biofilms are highly resistant to conventional antibiotics, specialized assays are used to evaluate the ability of MNPs to inhibit and disrupt biofilms. Recent studies have demonstrated that microenvironment‐responsive magnetic nanocomposites containing silver NPs and gentamicin can significantly disrupt biofilms when exposed to a magnetic field^[^ ^188^ ^]^ Additionally, the standard colony‐forming unit (CFU) counting method is used to quantify viable bacteria following MNP exposure, where serial dilutions of bacterial suspensions are plated on agar and the resulting colonies are counted.^[^ ^189^ ^]^

###### Recent Studies on MNPs for Antibacterial Application

MNPs have the potential to combat infections caused by bacteria and effectively address biofilm‐related problems. This effectiveness arises from mechanisms such as the generation of ROS, disruption of microbial membranes, and the release of toxic metal ions.

Over the past decade, there has been a significant focus on IONPs, particularly SPIONs, due to their potential antibacterial properties. In a study conducted by Zhang and Miao in 2024, it was found that ferrous ions released from SPIONS inhibited the growth of *Staphylococcus aureus* and *E. coli* under aerobic conditions. This suggests that IONPs may serve as a potential source of antibacterial ferrous ions.

Additionally, another study by Faisal et al.^[^ ^190^ ^]^ reported the synthesis of iron NPs functionalized with anthranilic acid through microwave‐induced precipitation. These functionalized NPs demonstrated enhanced antibacterial activity against methicillin‐resistant *Staphylococcus aureus* (MRSA). The results showed that the as‐synthesized functionalized NPs had superior antibacterial effects compared to nonfunctionalized ones, highlighting the importance of surface modifications in enhancing antibacterial action.

Nanocomposites based on magnetite have demonstrated remarkable antibacterial effectiveness in certain situations, particularly when loaded with antibiotics. Caciandone et al.^[^ ^191^ ^]^ prepared magnetite NPs functionalized with streptomycin‐ and neomycin to target Gram‐negative *Pseudomonas aeruginosa* and Gram‐positive *S. aureus*. Their research revealed that the MICs for *P. aeruginosa* were twice as low as those for *S. aureus*. These nanocomposites exemplify the development of targeted antibacterial therapies.

Research on antimicrobial NPs has explored a new area involving metal‐doped IONPs. Tasnim et al.^[^ ^179^ ^]^ reported the development of ZnFe_2_O_4_, as well as Cu‐ and Se‐doped IONPs, which exhibited significant bacterial inhibition zones of 20 mm or greater against various strains including *Bacillus subtilis*, *Klebsiella pneumoniae*, *E. coli, S. aureus*, and *Proteus vulgaris*. Additionally, Saba et al.^[^ ^184^ ^]^ investigated Ni‐doped Fe_3_O_4_/ZnO NPs and found that increasing the concentration of the Ni dopant reduced the size of the NPs and enhanced their antibacterial activity, achieving a zone of inhibition of 14 mm for *S. aureus*. These findings underscore the important role of dopants in modulating the antibacterial properties of MNPs.

Core–shell nanostructures have been investigated for their effectiveness as antibacterial agents. Imran et al.^[^ ^192^ ^]^ synthesized core–shell NPs of ZnO@NiO with an average crystal size of 13.059 nm. These NPs exhibited higher antibacterial activity that increased proportionally with the concentration of the NPs. Similarly, Saravanan et al. reported a significant reduction in the viability of *S. aureus* and *E. coli*, which dropped below 8% after 12 h of incubation with chitosan‐coated nickel oxide core–shell nanocomposites. This demonstrates the promising antimicrobial applications of core–shell architectures against both types of bacteria.

Disrupting biofilms remains one of the critical challenges in antimicrobial therapy, but MNPs have shown promising results in tackling this problem. Grumezescu et al.^[^ ^193^ ^]^ explored the use of Fe_3_O_4_ NPs loaded with sodium lauryl sulfate and cephalosporins, demonstrating their effectiveness in significantly reducing biofilm formation on various surfaces. Additionally, MgFe_2_O_4_ NPs exhibited potent antibiofilm activity at a low concentration of 10 μg ml^−1^ against both *E. coli* and *S. aureus.* ^[^ ^194^ ^]^ Furthermore, Zhang and Miao (2024) highlighted that mesoporous Fe_3_O_4_ NPs, when combined with AMF treatment, showed remarkable improvements in antibacterial activity, achieving a 99.15% reduction in viability for *E. coli* and a 79.88% viability reduction for *S. aureus.* ^[^ ^183^ ^]^

Other MNPs tested against microbes include copper oxide (CuO) and maghemite (Fe_2_O_3_) NPs. Studies have shown that CuO is more effective against MRSA and *E. coli* compared to maghemite.^[^ ^195^ ^]^ However, some research suggests that MNPs may not inhibit biofilm formation and could even promote bacterial growth under certain conditions.^[^ ^196^, ^197^ ^]^ Despite these varying findings, the antimicrobial effectiveness of iron ions in MNPs remains high.^[^ ^198^ ^]^

The surface charge and functionalization are crucial factors influencing the antibacterial effectiveness of MNPs. Javanbakht et al.^[^ ^199^ ^]^ demonstrated that SPIONs with a positive charge exhibited a stronger bacterial killing effect compared to their negatively charged counterparts. Wu et al.^[^ ^200^ ^]^ showed that chitosan‐coated IONPs increased ROS production, resulting in enhanced antibacterial activity. Similarly, Al‐Momani et al. biosynthesized Ag NPs with an average diameter of 11 nm, which showed MIC values of 15.6 μg ml^−1^ and successfully inhibited biofilm formation of *P. aeruginosa* at subinhibitory concentrations. These findings emphasize the importance of optimizing surface properties to maximize the antimicrobial potential of MNPs. Furthermore, Table S3, Supporting Information, provides an interesting overview of recent studies focusing on the development of magnetic nanocomposites, their antibacterial activities, and the types of bacteria tested in each investigation.

MNPs represent a versatile and potent platform for combating biofilms and multidrug‐resistant infections, with functionalization and synergistic strategies significantly enhancing their efficacy. Continued research is essential to refine their selectivity and safety, while addressing regulatory and environmental considerations will be crucial for their broader clinical and environmental deployment.

## 4 Use of MNPs in Agriculture

Agricultural pollution arises from the excessive and inappropriate use of pesticides and fertilizers, as well as practices associated with large‐scale farming and intensive animal husbandry. It has become a pressing global environmental concern.^[^ ^186^, ^201^ ^]^ Agricultural activities release a variety of pollutants, including organic compounds, such as phenolic substances, azo dyes, phthalates, and pesticides, and inorganic contaminants, notably heavy metals like arsenic, lead, mercury, and cadmium, along with inorganic salts. These organic and inorganic pollutants pose significant environmental and health risks due to their biotoxicity, thereby undermining efforts toward sustainable development.^[^ ^202^, ^203^ ^]^ In response, various strategies have been developed to detect and monitor pollutants originating from agricultural sources.

**Figure 9:**
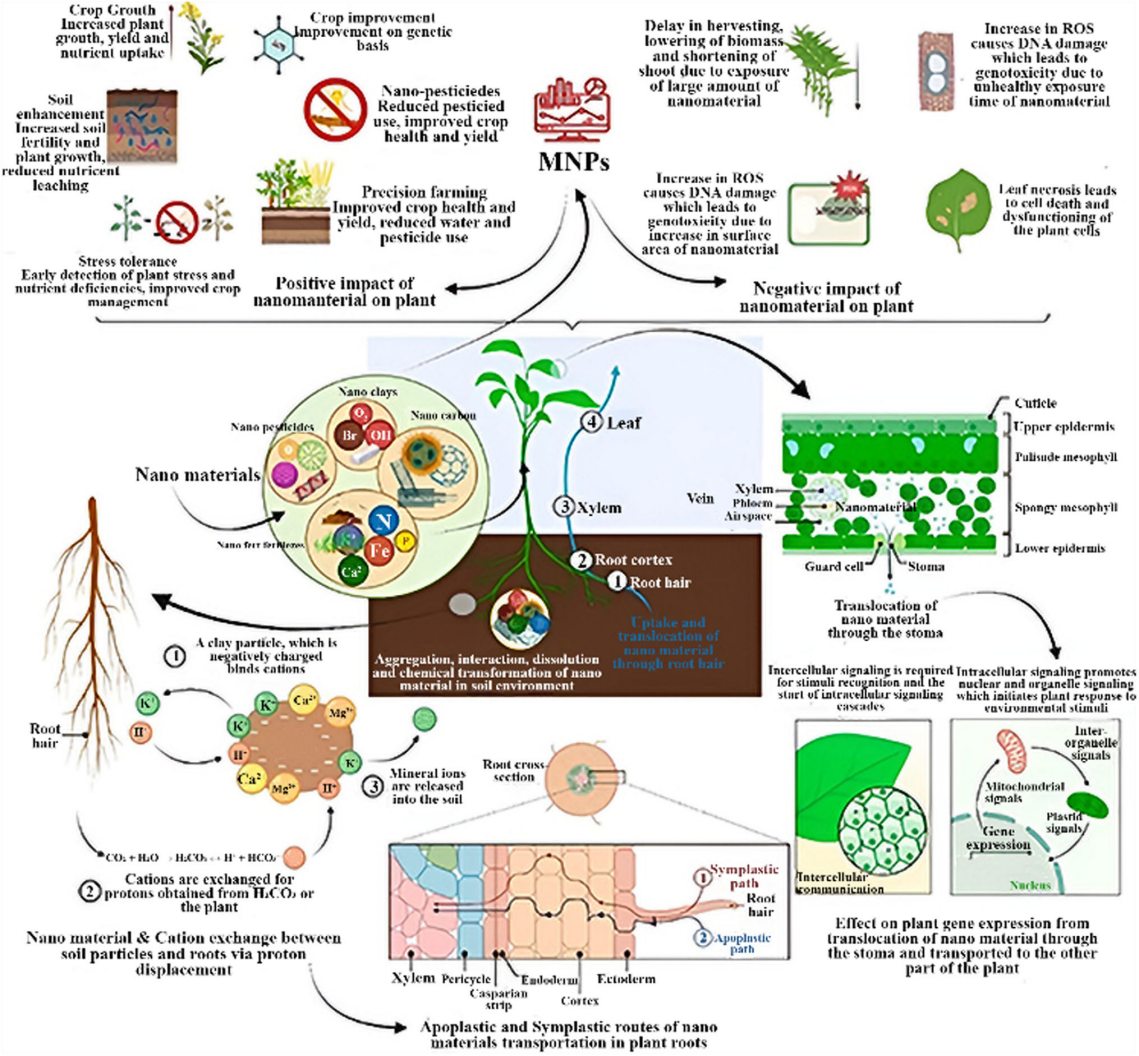
A depiction of the potential benefits and drawbacks of utilizing NPs in agriculture. Reproduced (Adapted) with permission.^[^ ^207^ ^]^ Copyright 2024, American Chemical Society.

**Figure** **9** summarizes some of the possible advantages and disadvantages of the use of NPs in agriculture. Application of NPs increases crop growth, soil fertility, and thus, decreases the application of chemical fertilizers and pesticides. They promote water retention in the soil, regulation of plant growth, optimization of land use, and quality and quantity of food output. However, toxicity and environmental hazards remain significant concerns. They could leach into water, contaminate the food chain, and, in the process, be toxic to human health and the ecosystem. The use of nanotechnology in agriculture requires a detailed assessment of associated risks and benefits, considering the ethical, social, and legal implications of this introduction. While nanomaterials offer enormous benefits, their interaction with plants and the environment remains largely unexplored and warrants further research.

For instance, the effects of NPs on plant physiology and metabolism, including their possible accumulation in the food chain and longer‐term consequences for soil microbiota and ecosystem functioning, are not well understood. Fully addressing such knowledge gaps and concomitant risks associated with the use of nanomaterials in agriculture will require interdisciplinary collaboration underpinned by sound regulatory frameworks. Coordinated efforts among scientists, policymakers, and stakeholders are urgently needed to ensure the responsible and safe deployment of nanotechnology, minimizing environmental and health risks while maximizing its benefits in agriculture.^[^ ^204^, ^205^, ^206^, ^207^ ^]^

Numerous studies have demonstrated the successful application of NPs in enhancing plant protection, promoting seed germination, and improving soil quality.^[^ ^208^, ^209^ ^]^ For instance, iron oxide MNPs have proven effective as nutrient supplements in soil, contributing to increased crop yields while minimizing environmental harm.^[^ ^210^ ^]^ Iron plays a crucial role in various physiological processes, including respiration, chlorophyl synthesis, biosynthetic pathways, and redox reactions. Many crops, particularly peanuts, are prone to iron deficiency, which adversely affects growth and productivity. In this context, several studies have explored the use of iron‐based NPs to improve iron uptake in plants, thereby mitigating nutrient deficiencies and supporting overall plant health.^[^ ^206^, ^207^ ^]^

### 4.1 Pesticides and Herbicides

MNPs offer a promising approach to improving pesticide and herbicide performance while minimizing their environmental footprint in agriculture. Various studies published in recent times are proving some new applications in this direction. One of the most notable approaches involves the use of MNPs in magnetic solid‐phase extraction. For example, Gamonchuang et al. (2022) synthesized magnetic MOFs derived from benzoate ligands for the magnetic solid‐phase extraction of carbamate pesticides. Their method demonstrated high adsorption capacities ranging from 2193–4196 mg kg^−1^ and achieved detection limits as low as 0.005–0.090 μg L^−1^ when coupled with high‐performance liquid chromatography. Such techniques are increasingly being employed to monitor and mitigate pesticide contamination in fruits and vegetables.^[^ ^211^ ^]^

MNPs have also been explored for the removal of herbicides from water and soil. In one study, Lu Yang et al. employed polyionic liquid‐functionalized, silica‐coated MNPs for the extraction of sulfonylurea herbicides from soil samples. The method demonstrated strong analytical performance, achieving detection limits between 1.62 and 2.94 ng mL^−1^ highlighting its significant potential for environmental monitoring and remediation.^[^ ^212^ ^]^ The researchers have also developed Fe_3_O_4_/graphene aerogel composites for the removal of herbicide 2,4‐dichlorophenoxyacetic acid or 2,4‐D from water. These composites allow for facile separation and reuse of the adsorbent material due to their magnetic properties, exhibiting a saturation magnetization of 20.66 emu g^−1^.^[^ ^213^ ^]^

**Figure 10:**
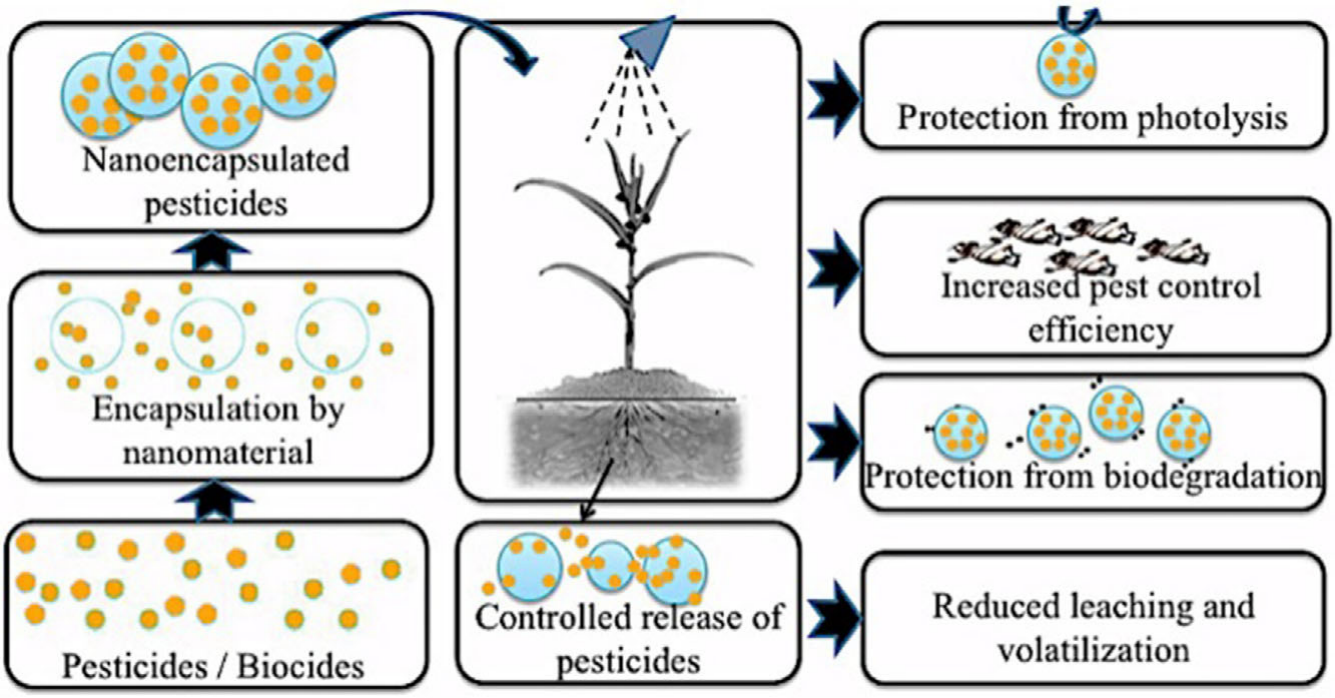
Nanoencapsulation process of the nanomaterials. Reproduced (Adapted) with permission.^[^ ^214^ ^]^ Copyright 2016, American Chemical Society.

Nanoencapsulation is the process of enclosing one substance within another material at the nanoscale, as shown in **Figure** **10**. The substance to be encapsulated, typically pesticides, is referred to as the internal phase, core material, filler, or fill, whereas the encapsulating material, such as nanocapsules, is known as the external phase, shell, coating, or membrane. This technology has been tested for encapsulating commercial pesticides and biocides, with the aim of enhancing their physical properties and facilitating their controlled application. The word nanoencapsulation of pesticides can be explained in simple words as the preparation of pesticide‐loaded or pesticide‐containing particles, having at least one dimension in the nanoscale, with dimensions in the range from 1–100 nm,^[^ ^214^ ^]^ there has been some controversy about the use of particle size in NP colloidal pesticide system.^[^ ^215^ ^]^ Kah et al. proposed that the size of nanopesticides can range from 1 to 1000 nm.^[^ ^215^ ^]^ In contrast, other studies have reported that the actual size of encapsulated pesticides labeled “nano” was mostly more significant than 100 nm, probably due to enhanced efficacy associated with their small particulate size. Grillo et al. have noted that in fields such as medicine and agriculture—where the conventional size threshold of  <100 nm for defining NPs may be applied more flexibly—particles exceeding 100 nm can still exhibit distinct nanoscale behaviors and novel physicochemical properties.^[^ ^214^, ^216^ ^]^

Pesticide nanoencapsulation in MNPs has emerged as a strategy to enhance its efficacy while reducing environmental impact. Thereafter, specific mechanisms for improving effectiveness, particularly stability, targeted delivery, and controlled release, come into action. For instance, polymeric NPs loaded with imidacloprid showed a 200‐fold reduction in required concentration compared to commercial formulations against crop pests.^[^ ^211^ ^]^ Nanoencapsulation of herbicides with polymeric NPs has also been shown to enhance the efficiency of weed control and protection of active ingredients in crop fields. Nanoencapsulated metribuzin, for example, exhibited higher efficiency in weed control against *Ipomoea grandifolia* even at lower doses compared to the conventional formulation.^[^ ^217^ ^]^ MNPs functionalized with specific ligands have been used to develop targeted delivery systems for pesticides, which permit reduced dosages and minimized environmental impact. For example, poly(lactic*‐co*‐glycolic acid) NPs loaded with imidacloprid showed 200 times lower required concentration compared to commercial formulations against crop pests.^[^ ^218^ ^]^

However, there are challenges associated with the use of MNPs in pesticide and herbicide applications, including their accumulation in soil and their impact on nontarget organisms. Further studies should be conducted on the long‐term environmental fate and toxicity of such materials. Additionally, the scalability and cost of MNP‐based pesticide formulations may further limit their widespread use in agriculture. Despite the challenges, the future outlook for MNPs in pesticide and herbicide applications still sounds promising. Further research is currently developing towards green, highly effective nanoformulations that enhance residual pesticide detection and effective delivery at the target site. As this field continues to advance, it is essential to contextualize future developments within a framework that balances the anticipated benefits with a comprehensive assessment of associated risks, in order to fully realize the potential of nanotechnology for sustainable agriculture.

### 4.2 Fertilizers and Plant Growth

Nanofertilizers represent a promising strategy for enhancing plant mineral nutrition. Several studies have demonstrated that nanomaterials can outperform conventional fertilizers by enabling controlled nutrient release, which facilitates improved nutrient uptake by plants while minimizing environmental losses due to reduced leaching and volatilization. However, contrasting reports suggest that in some cases, nanomaterials may exhibit comparable or even lower efficacy than traditional fertilizers. NPs utilized for nutrient delivery have the potential to enhance fertilizer use efficiency, reduce application rates, and mitigate the risk of nutrient runoff.^[^ ^219^ ^]^ Among the innovative developed to enhance fertilizer efficiency and promote plant growth in agriculture, MNPs have emerged. Fe and Zn metal nanocitrates have shown promising results when applied as plant nutrients in soil. Keerthi et al. studied the behavior of Fe and Zn nanocitrates in soil and their uptake by groundnut seedlings. Combined nanocitrate compositions showed the highest available Fe and Zn soil content after leaching, with 150.5 and 18.9 mg kg^−1^, respectively, against 6.0 and 0.7 mg kg^−1^ in untreated soil. The plant Fe content increased to 0.48 mg/pot for the combined nanocitrate composition, compared to 0.02 mg/pot in the untreated plants. Similarly, for pure zinc citrate, plant Zn content reached 82.3 μg/pot, while for untreated plants, it was 2.1 μg/pot. The results suggest a potential application of nanocitrates in enhancing seed quality and their ability to uptake Fe and Zn nutrients through the plant, outperforming commercial fertilizers in this regard.^[^ ^220^ ^]^

Iron is involved in various physiological processes of plants such as biosynthesis of photosynthetic pigments, photosynthesis, and respiration.^[^ ^221^ ^]^ Some studies have reported that Fe‐based NPs improve growth under nonstress conditions. For instance, the research of Liu et al. pointed out that lettuce seedlings in an aqueous medium at different concentrations showed positive effects on shoot length in a range of 12%−26% at low FeOXNPs concentrations of 5–20 mg L^−1^.^[^ ^222^ ^]^ Ghafariyan et al. reported increased chlorophyl content by the use of Fe_2_O_3_NPs at 30–60 mg L^−1^ applications in soybeans grown in hydroponics.^[^ ^223^ ^]^ Li et al. reported a 27.2% increase in germination rate and an 11.5% increase in root length among maize seedlings grown under hydroponic conditions using 20 mg L^−1^ γ‐Fe_2_O_3_. Even at very low concentrations, plant growth showed a positive response to the application of NPs containing iron oxide.^[^ ^224^ ^]^ Palchoudhury et al. found that plant soaking with α‐Fe_2_O_3_NPs, even as low as 5.54 × 10–3 mg L^−1^, enhanced their root lengths by as much as 88%−366% over those without any treatment. More recently, Rui et al. investigated Fe_2_O_3_ NPs as an alternative to conventional Fe‐based fertilizers.^[^ ^225^ ^]^ Also, it has been documented that under drought stress, γ‐ Fe_2_O_3_ NPs reduce oxidative stress in *Brassica napus* plants by significantly lowering the H_2_O_2_ and malondialdehyde levels at 2 and 1 mg mL^−1^, respectively.^[^ ^226^ ^]^ The design of Fe‐based NPs can be used for multifunctional alterations and may contribute valuable insights into further nanoagrochemical studies.

Iron oxide NPs have also been used to help in the iron deficiency of plants. An experiment was conducted by Ju et al. using magnetic particle spectroscopy (MPS) for the detection and distribution of EDTA‐capped IONPs in garden cress. They recorded a rise in total biomass as eightfold, but chlorophyl production enhanced by 1.4 times with respect to the plants treated with Fe‐EDTA commercial chelated iron fertilizer. This work demonstrates the potential of IONPs as efficient iron fertilizers and highlights the use of MPS as a reliable analytical tool for studying IONP uptake in plants.^[^ ^227^ ^]^

**Figure 11:**
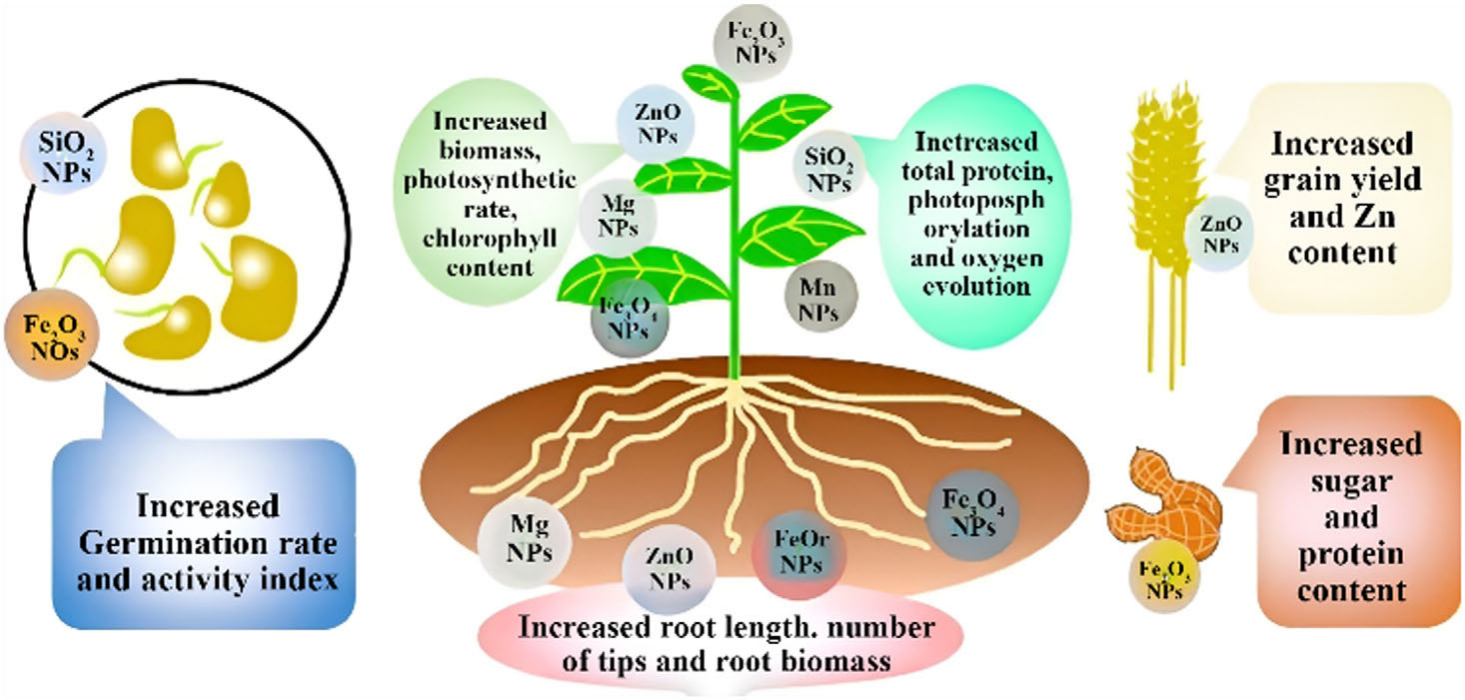
The influence of nanofertilizers on seed germination, plant growth, biomass, and grain yield, the content of valuable plant products. Reproduced (Adapted) with permission.^[^ ^226^ ^]^ Copyright 2020, American Chemical Society.

Functionalized magnetic nanomaterials have been able to enhance crop yields and deliver agrochemicals and nutrients in an effective and targeted manner. For example, *β*‐keratinase‐bound MNPs were utilized for the conversion of chicken feathers into organic products, which are valuable for seed germination and plant growth. The application of this organic fertilizer in the soil enhanced the germination of Bengal gram seeds, resulting in increased plant height, fresh biomass, and a higher microbial population in the soil.^[^ ^228^ ^]^ Calcium‐ and magnesium‐enriched nanohexaferrites have shown positive effects on barley plants in hydroponic systems. At specific concentrations, the NPs led to increases in germination rate, tissue growth, biomass, protein content, and chlorophyl pigments compared to untreated samples. The study also demonstrated NP uptake by the plants, with increased concentrations of Fe, Ca, Mg, and Sr detected in the plant's leaves.^[^ ^227^, ^228^ ^]^ Hence, NPs enhanced photosynthesis by improving light harvesting, electron transport, and scavenging ROS as summarized.^[^ ^226^, ^229^, ^230^ ^]^ The application of nanofertilizers to improve plant growth and productivity in agriculture as is shown in **Figure** **11**.^[^ ^226^ ^]^

However, the use of MNPs in agriculture presents certain challenges. Conflicting reports have been obtained in a few studies, which may be linked to the variation in their experimental parameters, such as the size and shape of NPs, surface coating, plant types, and growth conditions. Additionally, the probability of NPs accumulating in soil and their potential effects on nontarget organisms is a cause for concern. Their long‐term environmental fate and toxicity need further investigation.^[^ ^227^ ^]^

Despite various challenges, the future also looks promising for MNPs in agriculture. The latest research focuses on developing greener and more efficient nanoformulations, enhancing residual NP detection methods, and improving nutrient delivery to the site of action. It is thus anticipated that this new development, along with its balanced assessment of benefits and potential risks, will enable one to fully leverage this technology in sustainable agriculture.

### 4.3 Food Safety in Agriculture and Fertilizer

Food safety and quality play an immense role in relation to public health. Nowadays, an increasing number of consumers require food to be free from any contamination, whether during production or packaging. The contaminants that are usually present from chemical ones up to biological agents can pose very serious health risks, leading to a wide variety of problems such as food toxicity, malnutrition, and gastrointestinal diseases, among others. Most conventional methods are time‐consuming and expensive. Faster and more precise tools need to be developed.

**Figure 12:**
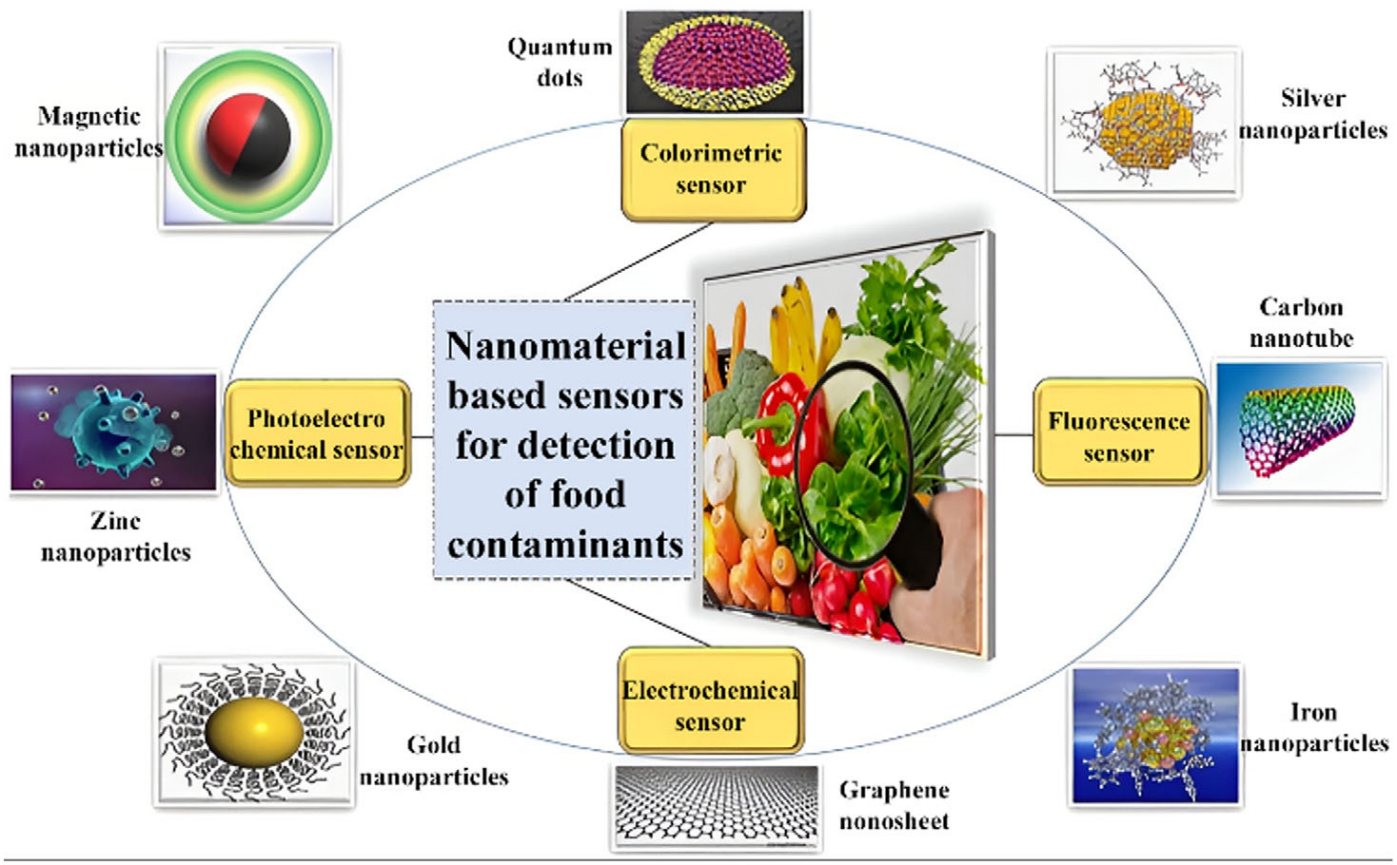
Schema of metal nanomaterials for food contaminant detection. Reproduced (Adapted) with permission.^[^ ^248^ ^]^ Copyright 2024, Taylor and Francis.

Nanotechnology offers new solutions in detecting food toxicity, while nanosensors are poised to revolutionize food packaging and storage.^[^ ^231^ ^]^ **Figure** **12** provides an overview of metal nanomaterials for detecting food contaminants. Agriculture is showing keen interest in addressing food safety issues related to chemical contaminants, such as pesticides, mycotoxins, and allergens, which pose significant risks to public health. The applications of MNPs in food analysis involve sample preconcentration and extraction, as well as the enhancement of sensitivity in detection. Most of them are typically modified with specific ligands, which enable them to selectively capture contaminants, and the contaminants are then analyzed using techniques, such as high‐performance liquid chromatography, polymerase chain reaction (PCR), and immunoassays. For example, Speroni et al. presented a magnetic particle‐based enzyme‐linked immunosorbent assay for the detection of peanut allergens, exhibiting a detection limit of 0.2 mg kg^−1^.^[^ ^232^ ^]^ In pathogen detection, Yang et al. utilized MNPs for bacteria preconcentration, which increased the sensitivity of subsequent PCR detection and reduced the limit of detection from 10^5^ to 10^2^ CFU mL^−1^.^[^ ^233^ ^]^ MNPs have also been utilized in the development of biosensors. For example, an impedimetric biosensor was constructed using MNPs conjugated with antibodies for the detection of *E. coli*, demonstrating a 35% increase in sensitivity. Additionally, MNP‐based biosensors have been applied in glucose monitoring, as well as in the detection of food adulterants, including herbicide and antibiotic residues. These systems have demonstrated high sensitivity and analytical efficiency, underscoring their potential in ensuring food safety and quality control.^[^ ^234^ ^]^

The application of MNPs as nanosensors for the rapid and sensitive detection of foodborne pathogens can be considered one of the most promising uses in food safety. For example, Banerjee et al. developed multiparametric magneto‐fluorescent nanosensors capable of detecting *E. coli* O157:H7 at concentrations as low as 1 CFU in pure culture and 8.0 × 10^5^ CFU mL^−1^ in ground beef samples, with a total assay time of only 35 min.^[^ ^235^ ^]^ Such sensitivity and speed far exceed the days required to obtain results using conventional culturing methods. By leveraging their magnetic properties, the nanosensors enable the efficient separation and concentration of target pathogens from complex food matrices, while their fluorescence properties provide for rapid and sensitive detection.

MNPs have also been utilized to enhance the extraction and detection of various contaminants in food. For example, Fe_3_O_4_ NPs modified with *γ*‐mercaptopropyl trimethoxysilane have been used for the determination of heavy metal ions in food samples at lower limits of detection. The thiol groups of thus functionalized MNPs interact efficiently with metal ions, enabling selective extraction and concentration and thereby enhancing the sensitivity of the previously developed methods.^[^ ^236^ ^]^ Further, MNPs have been utilized for the development of sensitive detection methods for foodborne pathogens. It was once illustrated using AB‐functionalized SPIONs and a highly effective high‐transition‐temperature superconducting quantum interference device to develop a biosensing technique capable of detecting *L. monocytogenes* at a low limit of 5.6 × 10^6^ cells 20^−1^ μL.^[^ ^237^ ^]^

While MNPs offer tremendous opportunities for improving food safety, there is a pressing need to consider the long‐term consequences on human health and environmental impact. Some research has identified that certain types of NPs may potentially accumulate in living organisms and persist in the environment, which could induce unforeseen effects. Thus, it is important that thorough safety and ecological risk assessments of MNPs be conducted before widespread applications of this technology in food safety.^[^ ^232^ ^]^

The application of MNPs holds considerable promise for improving food safety in agriculture, particularly through the rapid and sensitive detection of pathogens and chemical contaminants, as well as potential antimicrobial interventions. Moreover, the integration of MNPs in fertilizer management could contribute to more precise and efficient nutrient delivery. However, realizing these benefits requires sustained fundamental and applied research to better understand and mitigate the potential risks associated with MNP use. In conclusion, ongoing advances in nanotechnology suggest an expanding role for MNPs in enhancing the safety, quality, and sustainability of the food supply chain.

### 4.4 Water Purification in Agriculture

MNP‐based purification has great potential for irrigating water, reducing crop contamination risks, and further improving yields. Application of NPs in agriculture will not only contribute to the improvement of yields but also to minimizing the environmental impacts of agriculture and improving food safety and quality.^[^ ^238^ ^]^

Iron oxide NPs, mainly magnetite (Fe_3_O_4_), represent an effective application in water remediation, as reflected in a wide variety of aspects, such as a large surface area‐to‐volume ratio, superparamagnetic characteristics, and simple separation via external magnetic fields. Recently, the great capability of ultrafine mesoporous magnetite nanoparticles (UFMNPs) was proved in removing more than one heavy metal ion from river water in a single run. It was noticed that UFMNPs showed a removal efficiency of 98% for Pb^2+^, 87% for Cd^2+^, 90% for Cu^2+^, and 78% for Ni^2+^ ions in single‐ion systems. For mixed‐ion systems, the removal percentages decreased but still showed good efficiencies of 86% for Pb^2+^, 80% for Cd^2+^, 84% for Cu^2+^, and 54% for Ni^2+^, respectively.^[^ ^239^ ^]^ This underlines the potential of MNPs in tackling the challenges posed by complex, multicontaminant water systems that are commonly encountered in agriculture.

The application of MNPs in water purification has the potential to significantly enhance agricultural practices by improving both crop yield and quality. As noted by Zhuang and Gentry showed that the use of NPs for purifying irrigation water can substantially reduce the risk of crop contamination, thereby contributing to safer and healthier agricultural produce.^[^ ^238^ ^]^

Other applications beyond water purification include the study of the effects of MNPs for nutrient delivery and uptake in plants. A study recently conducted by Huang et al. on wheat plants used MnFe_2_O_4_ NPs as a foliar fertilizer. The results showed that at 100 mg L^−1^, NP dose NPs after three successive applications significantly improved the grain yield to 5.0 ± 0.12 t/ha and improved the harvest index to 0.46 ± 0.001. Besides, the treatment enhanced grain Fe, Mn, and Ca content and increased the crude protein from 13 ± 0.79% to 15 ± 0.58%.^[^ ^240^ ^]^ The findings thus suggest that MNPs can be utilized in agriculture for dual purpose: water purification and improving crop nutrition.

However, at the same time, potential environmental impacts due to MNPs in agriculture should also be considered. Sharma et al. drew on the fact that future studies are needed for better understanding of the NPs’ long‐term impact on microbial communities and ecosystem functions in soils.^[^ ^241^ ^]^

Overall, MNPs may serve well in applications aimed at improving water quality and enhancing crop productivity in agriculture. Besides their ability to take out more than one contaminant simultaneously, its nutrient carrier ability makes them a top tool for farming sustainably. However, further investigation is needed on the long‐term environmental impact of MNP and the optimization of efficiency under varying agricultural conditions. It is envisioned that with advancements in nanotechnology, even more innovative and effective MNP‐based solutions will emerge in water treatment and crop improvement.

Recent research has focused on the ability of MNPs to remove contaminants and enhance nutrient uptake, thereby increasing yield while addressing key challenges related to food safety and environmental sustainability. However, challenges persist regarding their environmental impact, bioaccumulation, scalability, and the development of appropriate regulatory frameworks. Further research is needed to focus on optimizing formulations, developing site‐targeting systems for MNP, and conducting a comprehensive safety evaluation to enable their responsible use in agriculture. By addressing these challenges and leveraging the numerous benefits that MNPs offer, the agricultural sector can adopt more sustainable and efficient practices that enhance food security while ensuring environmental health.

MNPs represent a new approach to ancient agriculture and hold much potential in several fields. For example, MNPs exhibited superior activity in improving the effectiveness of pesticides and herbicides, enhancing the transportation of fertilizers, and promoting plant growth. Functionalized MNPs are commonly used for detecting and eliminating pesticides and herbicides. Some magnetic MOFs have demonstrated high adsorption capacity and low detection limits, thereby, significantly improving the monitoring and control of pesticide contamination in agricultural products. Besides, functionalized MNPs have been found useful in the removal of herbicides from soil and water, thus providing a feasible solution for environmental remediation.

Fertilizer delivery and plant growth are other roles that MNPs have increased the nutrient use efficiency of plants. It was observed that the application of iron (Fe) and zinc (Zn) nanocitrates greatly increased soil nutrient content and plant absorption compared to traditional fertilizers. IONPs have become particularly interesting for improving iron deficiencies in plants due to their enhanced biomass production and chlorophyl synthesis. Given these benefits, MNPs have a role in helping agricultural productivity.

MNPs have also played a significant role in food safety, contributing to the detection and elimination of foodborne pathogens. Thus, functionalized SPIONs have demonstrated high sensitivity for detecting *L. monocytogenes* pathogens, while antibody‐functionalized gold nanorods have been utilized for targeting *P. aeruginosa*. These applications prove the utility of MNPs in ensuring the quality and safety of food.

The ease with which MNPs can be separated from water by using magnetic fields makes them promising for large‐scale treatment of agricultural water. Precision agriculture and smart farming are also emerging technologies that will gain much from the integration of MNPs. Further research is bound to make the solutions more efficient, targeted, and environmentally compatible for sustainable agriculture.

Despite their promise, several challenges face MNPs. The long‐term effects of MNPs on soil ecosystems and nontarget organisms are poorly understood, and their potential to accumulate in soil and the food chain raises serious concerns.^[^ ^223^, ^237^ ^]^ The large‐scale production and application of MNPs may prove economically unviable, hence limiting widespread adoption.^[^ ^223^, ^238^ ^]^

Additionally, since there are no specific laws governing the use of nanomaterials, their application in agriculture can be standardized accordingly. A detailed understanding of the interaction between MNP and the plant‐soil microorganisms interface still remains a significant knowledge gap.^[^ ^223^, ^240^ ^]^

Future research should prioritize the development of targeted delivery systems utilizing MNPs for precise and efficient application of nutrients and pesticides.^[^ ^223^, ^240^ ^]^ Another critical research direction involves exploring the role of MNPs in enhancing plant resilience to abiotic stresses, including drought, salinity, and temperature extremes. Such investigations could pave the way for more sustainable and stress‐resilient agricultural systems under changing climatic conditions.^[^ ^228^ ^]^ MNPs also hold potential in the field of biofortification, offering a promising strategy to address global micronutrient deficiencies by enhancing nutrient concentrations in food crops.^[^ ^242^ ^]^ To facilitate broader adoption, the development of ecofriendly and cost‐effective synthesis approaches, such as green synthesis using plant extracts, should be prioritized.^[^ ^23^ ^]^ Furthermore, comprehensive toxicity studies and life cycle assessments are critical to evaluate the long‐term environmental impacts, safety, and overall sustainability of MNP‐based agricultural products.^[^ ^237^, ^241^ ^]^

Establishing synergistic interactions between MNPs and plant growth‐promoting microorganisms represents a promising strategy to further enhance crop productivity.^[^ ^243^ ^]^ The development and adoption of standardized protocols for characterizing and applying MNPs are essential to ensure consistency, reproducibility, and comparability across research studies.^[^ ^244^ ^]^ In addition, advancements in MNP‐based nanosensors offer significant potential for real‐time monitoring of crop health and environmental parameters, thereby contributing to the advancement of precision agriculture.^[^ ^48^, ^240^ ^]^

## 5 Conclusion and Future Perspectives

In recent years, MNPs have emerged as highly versatile materials with the potential to significantly impact various fields, including water remediation, healthcare, antibacterial applications, and agriculture. Their unique magnetic properties, high surface area, and tunable surface chemistry have paved the way for innovative solutions to contemporary global challenges. This review highlights key advancements in the synthesis of MNPs, along with notable applications and future research directions.

In the medical field, MNPs have significantly advanced diagnosis and treatment, particularly in cancer management. These NPs enable more precise drug targeting, which reduces side effects and enhances therapeutic benefits. Techniques such as MHT can selectively heat and kill cancerous cells using MNPs. Additionally, advancements in MRI and magnetic particle imaging are being achieved with these particles. There is also growing interest in applying MNPs to combat drug‐resistant bacteria. This can be done by damaging cell membranes, generating ROS, and enhancing antibiotic effectiveness through exposure to magnetic fields.

Another important area of application is agriculture. MNPs can enhance the nutrient uptake efficiency of plants, reduce pesticide use, and aid in cleaning soil contaminants, thereby contributing to more environmentally friendly farming methods. They play a crucial role in precision agriculture by providing nanosensors for soil health, which can lead to improved crop yields. Furthermore, MNPs can be utilized in the biofortification of crops with essential nutritional elements, making it a vital step in combating global malnutrition.

MNPs are not just laboratory curiosities; they have the potential to significantly impact people's lives. As research in this area progresses, it fosters advancements in medical technology.

### 5.1 Challenges and Future Perspectives

Despite the broad potential of MNPs, several critical challenges must be addressed to enable their safe and effective deployment across various applications. Foremost among these are environmental and toxicity concerns. Comprehensive, long‐term studies are required to elucidate the biological interactions, ecotoxicological impacts, and potential for bioaccumulation of MNPs in ecosystems. The development of biodegradable and ecofriendly MNP formulations will be essential to ensure environmental sustainability.

Scalability and cost‐effectiveness also present substantial barriers. Bridging the gap between laboratory‐scale synthesis and industrial‐scale production demands scalable, economically viable methods that maintain the physicochemical integrity and functionality of MNPs. Furthermore, the lack of standardized protocols for synthesis, characterization, and risk assessment, along with the absence of harmonized regulatory frameworks, continues to impede broader commercial adoption.

From a functional perspective, improving the performance of MNPs remains a key research priority. Enhancements in selectivity, stability, and reusability are necessary to meet application‐specific requirements. In addition, challenges, such as NP agglomeration, reduced activity under dynamic or fluidized conditions, and surface fouling, must be addressed to maintain long‐term efficiency. Future research should also explore synergistic combinations of MNPs with other nanomaterials or biological agents to expand their multifunctionality and responsiveness in complex environments.

### 5.2 Innovative Recommendations

Several strategies may represent the next wave in the development of MNP technology. First, green synthesis methods that utilize biological templates, plant extracts, or microorganisms provide environmentally friendly approaches to reduce the ecological footprint of MNP preparation. Additionally, advanced functionalization techniques could enhance the specificity of MNPs toward targeted pollutants, pathogens, or plant nutrients, thereby improving their effectiveness in practical applications.

Designing hybrid systems that combine MNPs with other nanomaterials or technologies, such as MOFs, could lead to robust solutions for applications in water treatment, healthcare, and agriculture. Furthermore, stimuli–responsive nanomaterials, which can be activated by changes in pH, temperature, or magnetic fields, provide exceptional opportunities for TDD, pollutant removal, and precision agriculture.

Integrating MNP‐based nanosensors with artificial intelligence could facilitate real‐time monitoring and predictive maintenance related to crop health and environmental conditions in agriculture. Conducting mechanistic studies on how MNPs interact with biological systems, contaminants, and environmental matrices will be essential for optimizing their design and applications.

Performing life cycle assessments from synthesis to disposal will ensure sustainability and minimize ecological impact. Lastly, developing these techniques for use in existing industrial and healthcare systems will enhance their practicality and encourage broader acceptance.

The transformative potential of MNPs spans medicine, environmental protection, and agriculture, offering innovative solutions to various modern challenges. Ongoing research suggests that these nanoscale materials could revolutionize industries and improve daily life. However, key factors such as improved production scalability, safety, and regulatory frameworks are essential for selecting the appropriate processes and applications. Achieving these goals will require a collaborative effort among scientists, engineers, and policymakers. If implemented effectively, MNPs could become crucial components in the development of next‐generation technologies.

## Conflict of Interest

The authors declare no conflict of interest.

## Supporting information

Supplementary Material

## References

[1] W. Zaman , H. Manghwar , Microorganisms 2024, 12, 489. 38543540 10.3390/microorganisms12030489 PMC10974228

[2] S. Wang , Y. Hou , Adv. Sci. 2024, 11, 2305459. 10.1002/advs.202305459 PMC10885654 37988692

[3] M. Bustamante‐Torres , D. Romero‐Fierro , B. Arcentales‐Vera , S. Pardo , E. Bucio , Polym. Basel, 13, 2998, 2021. 10.3390/polym13172998 PMC8434030 34503038

[4] D. F. Picchi , C. Biglione , P. Horcajada , ACS Nanosci. Au 2023, 4, 85. 38644966 10.1021/acsnanoscienceau.3c00041 PMC11027209

[5] I. Ban , M. Drofenik , H. Bukšek , I. Petrinic , C. Helix‐Nielsen , S. Vohl , S. Gyergyek , J. Stergar , Environ. Sci.: Water Res. Technol. 2023, 9, 442.

[6] Y. Ha , S. Ko , I. Kim , Y. Huang , K. Mohanty , C. Huh , J. A. Maynard , Mater. 2018, 1, 512. 10.1021/acsanm.7b00025 PMC5999228 29911680

[7] Z. Dong , G. Si , X. Zhu , C. Li , R. Hua , J. Teng , W. Zhang , L. Xu , W. Qian , B. Liu , J. Wang , T. Wang , Y. Tang , Y. Zhao , X. Gong , Z. Tao , Z. Xu , Y. Li , B. Chen , X. Kong , Y. Xu , N. Gu , C. Li , Circ.: Cardiovasc. Imaging 2023, 16, 580. 37463240 10.1161/CIRCIMAGING.123.015404

[8] Y. Huang , J. C. Hsu , H. Koo , D. P. Cormode , Theranostics 2022, 12, 796. 34976214 10.7150/thno.67375 PMC8692919

[9] A. Pusta , M. Tertis , I. Crăciunescu , R. Turcu , S. Mirel , C. Cristea , Pharm. 2023, 15, 1872. 10.3390/pharmaceutics15071872 PMC10383769 37514058

[10] Ali. Farzin , S. Alireza Etesami , J. Quint , A. Memic , A. Tamayol , Adv. Healthc. Mater. 2020, 9, 1901058. 10.1002/adhm.201901058 PMC7482193 32196144

[11] M. Muthu Meenakshi , G. Annasamy , S. Krishnan , M. Hema Brindha , A. K. Narasimhan , Engineered Magnetic Nanoparticles As Environmental Remediation Agents, https://books.rsc.org/books/edited‐volume/2127/chapter/7696424/Engineered‐Magnetic‐Nanoparticles‐as‐Environmental (accessed: January 2025).

[12] H. Veisi , M. Pirhayati , P. Mohammadi , T. Tamoradi , S. Hemmati , B. Karmakar , RSC Adv. 2023, 13, 20530. 37435379 10.1039/d3ra01208e PMC10331794

[13] G. Dee , O. O’Donoghue , E. Devitt , T. Giroud , A. Rafferty , L. Gannon , C. McGuinness , Y. K. Gun’ko , ACS Omega 2024, 9, 4347. 38313544 10.1021/acsomega.3c06593 PMC10832022

[14] R. Bushra , M. Ahmad , K. Alam , F. Seidi , Qurtulen, S. Shakeel, J. Song, Y. Jin, H. Xiao , Sustainable Mater. Technol. 2024, 40, e00985.

[15] D. Chen , Q. Tang , X. Li , X. Zhou , J. Zang , W. Xue , J. Xiang , C. Guo , Int. J. Nanomed. 2012, 7, 4973. 10.2147/IJN.S35140 PMC3446860 23028225

[16] M. Keshtkar , D. Shahbazi‐Gahrouei , M. A. Mehrgardi , M. Aghaei , S. M. Khoshfetrat , J Biomed Phys. Eng. 2018, 8, 357. 30568925 PMC6280118

[17] A. A. Akintola , S. Yahaya , Int. J. Res. Publ. Rev. 2024, 5, 843.

[18] R. Baby , M. Z. Hussein , A. H. Abdullah , Z. Zainal , Polym. Basel 2022, 14, 583. 10.3390/polym14030583 PMC8838446 35160572

[19] A. Karnwal , T. Malik , Front. Environ. Sci. 2024, 12, 1393694.

[20] V. Chikan , E. J. McLaurin , Nanomater. Basel 2016, 6, 85. 10.3390/nano6050085 PMC5302497 28335212

[21] J. C. Rendón‐Angeles , G. Seong , Nanomater. Basel 2023, 13, 1463. 10.3390/nano13091463 PMC10180294 37177008

[22] K. A. Altammar , Front. Microbiol. 2023, 14, 1155622. 37180257 10.3389/fmicb.2023.1155622 PMC10168541

[23] H. Singh , M. F. Desimone , S. Pandya , S. Jasani , N. George , M. Adnan , A. Aldarhami , A. S. Bazaid , S. A. Alderhami , Int. J. Nanomed. 2023, 18, 4727. 10.2147/IJN.S419369 PMC10444627 37621852

[24] B. Bhardwaj , P. Singh , A. Kumar , S. Kumar , V. Budhwar , Adv. Pharm. Bull. 2020, 10, 566. 33072534 10.34172/apb.2020.067 PMC7539319

[25] J. Hong , L. Wang , Q. Zheng , C. Cai , X. Yang , Z. Liao , Mater. Basel 2024, 17, 2870. 10.3390/ma17122870 PMC11204782 38930238

[26] B. Issa , I. M. Obaidat , B. A. Albiss , Y. Haik , Int. J. Mol. Sci. 2013, 14, 21266. 24232575 10.3390/ijms141121266 PMC3856004

[27] Z. Ma , J. Mohapatra , K. Wei , J. P. Liu , S. Sun , Chem. Rev., 123, 3904. 34968046 10.1021/acs.chemrev.1c00860

[28] H.‐L. Duan , Z.‐Q. Shen , X.‐W. Wang , F.‐H. Chao , J.‐W. Li , World J Gastroenterol. 2005, 11, 3660. 15968716 10.3748/wjg.v11.i24.3660 PMC4316012

[29] S. P. Ravindranath , L. J. Mauer , C. Deb‐Roy , J. Irudayaraj , Anal. Chem. 2009, 81, 2840. 19281189 10.1021/ac802158y

[30] A. Ali , T. Shah , R. Ullah , P. Zhou , M. Guo , M. Ovais , Z. Tan , Y. Rui , Front. Chem. 2021, 9, 10.3389/fchem.2021.629054. PMC8314212 34327190

[31] N. Rarokar , S. Yadav , S. Saoji , P. Bramhe , R. Agade , S. Gurav , P. Khedekar , V. Subramaniyan , L. S. Wong , V. Kumarasamy , Int. J. Pharm.: X 2024, 7, 100231. 38322276 10.1016/j.ijpx.2024.100231 PMC10844979

[32] L. Gandarias , R. L. Kimber , G. Ona‐Nguema , Elements, 19, 228, 2023.

[33] R. C. Popescu , E. Andronescu , B. S. Vasile , Nanomater. Basel, 9, 1791, 2019. 10.3390/nano9121791 PMC6956201 31888236

[34] G. Masciocchi , J. W. van der Jagt , M.‐A. Syskaki , J. Langer , G. Jakob , J. McCord , B. Borie , A. Kehlberger , D. Ravelosona , M. Kläui , Phys. Rev. Appl. 2023, 20, 014001.

[35] H. M. Williams , Bioscience Horizons: Int. J. Stud. Res. 2017, 10, hzx009.

[36] S. Mukherjee , L. Liang , O. Veiseh , Pharm. 2020, 12, 147. 10.3390/pharmaceutics12020147 PMC7076668 32053995

[37] S. Khizar , N. M. Ahmad , N. Zine , N. Jaffrezic‐Renault , A. Errachid‐el‐salhi , A. Elaissari , ACS Appl. Nano Mater. 2021, 4, 4284.

[38] A. Ullah Khan , L. Chen , G. Ge , Inorg. Chem. Commun. 2021, 134, 108995. 34658663 10.1016/j.inoche.2021.108995 PMC8500685

[39] N. Sezer , İ. Arı , Y. Biçer , M. Koç , J. Magn. Magn. Mater. 2021, 538, 168300.

[40] E. M. Materón , C. M. Miyazaki , O. Carr , N. Joshi , P. H. S. Picciani , C. J. Dalmaschio , F. Davis , F. M. Shimizu , Appl. Surface Sci. Adv. 2021, 6, 100163.

[41] S. Bai , X. Zhang , Y. Zou , Y. Lin , Z. Liu , K. Li , P. Huang , T. Yoshida , Y. Liu , M. Li , W. Zhang , X. Wang , M. Zhang , C. Du , Front. Bioeng. Biotechnol. 2024, 12, 1382085. 38572358 10.3389/fbioe.2024.1382085 PMC10987818

[42] S. Vohl , M. Kristl , J. Stergar , Nanomater. Basel 2024, 14, 1179. 10.3390/nano14141179 PMC11279442 39057856

[43] J. Dulińska‐Litewka , A. Łazarczyk , P. Hałubiec , O. Szafrański , K. Karnas , A. Karewicz , Mater. Basel 2019, 12, 617. 10.3390/ma12040617 PMC6416629 30791358

[44] Kritika, I. Roy , Mater. Adv., 3, 7425, 2022.

[45] C. Pucci , A. Degl’Innocenti , M. B. Gümüş , G. Ciofani , Biomater. Sci. 2022, 10, 2103. 35316317 10.1039/d1bm01963e

[46] J. Göransson , T. Zardán Gómez De La Torre , M. Strömberg , C. Russell , P. Svedlindh , M. Strømme , M. Nilsson , Anal. Chem. 2010, 82, 9138. 20977277 10.1021/ac102133e

[47] F. D. Guerra , M. F. Attia , D. C. Whitehead , F. Alexis , Molecules 2018, 23, 1760. 30021974 10.3390/molecules23071760 PMC6100491

[48] K. Zhang , X. Song , M. Liu , M. Chen , J. Li , J. Han , Water 2023, 15, 3077.

[49] A. Yadollahpour , S. Rashidi , Orient. J. Chem. 2015, 312015, 25.

[50] A. G. Díez , M. Rincón‐Iglesias , S. Lanceros‐Méndez , J. Reguera , E. Lizundia , Mater. Today Chem. 2022, 26, 101220.

[51] G. Kasparis , A. P. Sangnier , L. Wang , C. Efstathiou , A. P. LaGrow , A. Sergides , C. Wilhelm , N. T. K. Thanh , J. Mater. Chem. B 2023, 11, 787. 36472454 10.1039/d2tb01338j PMC9890495

[52] P. Kowalik , J. Mikulski , A. Borodziuk , M. Duda , I. Kamińska , K. Zajdel , J. Rybusinski , J. Szczytko , T. Wojciechowski , K. Sobczak , R. Minikayev , M. Kulpa‐Greszta , R. Pazik , P. Grzaczkowska , K. Fronc , M. Lapinski , M. Frontczak‐Baniewicz , B. Sikora , J. Phys. Chem. C Nanomater Interfaces 2020, 124, 6871. 32952770 10.1021/acs.jpcc.9b11043 PMC7497709

[53] E. S. Vasquez , E. M. Prehn , K. B. Walters , J. Therm. Anal. Calorim 2021, 143, 35.

[54] R. T. Busch , F. Karim , Y. Sun , H. C. Fry , Y. Liu , C. Zhao , E. S. Vasquez , Front. Nanotechnol. 2021, 3, 10.3389/fnano.2021.653744.

[55] A. Zeleňáková , V. Zeleňák , E. Beňová , B. Kočíková , N. Király , P. Hrubovčák , J. Szűcsová , Ľ. Nagy , M. Klementová , J. Mačák , V. Závišová , J. Bednarčík , J. Kupčík , A. Jacková , D. Volavka , J. Košuth , Š. Vilček , Sci. Rep. 2024, 14, 14427. 38910140 10.1038/s41598-024-64839-2 PMC11194262

[56] M. Bañobre‐López , A. Teijeiro , J. Rivas , Reports of Practical Oncology & Radiotherapy 2013, 18, 397. 24416585 10.1016/j.rpor.2013.09.011 PMC3863197

[57] V. Vilas‐Boas , F. Carvalho , B. Espiña , Molecules, 2020, 25, 2874. 32580417 10.3390/molecules25122874 PMC7362219

[58] J. Jose , R. Kumar , S. Harilal , G. E. Mathew , D. G. T. Parambi , A. Prabhu , M. S. Uddin , L. Aleya , H. Kim , B. Mathew , Environ Sci Pollut Res 2020, 27, 19214. 10.1007/s11356-019-07231-2 31884543

[59] P. Ilg , M. Kröger , Phys. Chem., Chem. Phys., 2020, 22, 22244. 33001111 10.1039/d0cp04377j

[60] X. Yu , R. Yang , C. Wu , B. Liu , W. Zhang , Sci Rep 2022, 12, 16055. 36163493 10.1038/s41598-022-20558-0 PMC9513098

[61] J. V. Nuzhina , A. A. Shtil , A. Y. Prilepskii , V. V. Vinogradov , J. Drug Delivery Sci. Technol. 2019, 54, 101282.

[62] S. Keshri , S. Biswas , Prog. Biomater. 2022, 11, 347. 36163543 10.1007/s40204-022-00204-8 PMC9626731

[63] M. G. Miguel , J. P. Lourenço , M. L. Faleiro , Int. J. Mol. Sci. 2020, 21, 6633. 32927821 10.3390/ijms21186633 PMC7555169

[64] D. L. Huber , Small 2005, 1, 482.17193474

[65] Z. Ren , R. Fu , L. Sun , H. Li , Z. Bai , Y. Tian , G. Zhang , Sci. Total Environ. 2024, 915, 169852. 38190907 10.1016/j.scitotenv.2023.169852

[66] W. Xu , T. Yang , S. Liu , L. Du , Q. Chen , X. Li , J. Dong , Z. Zhang , S. Lu , Y. Gong , L. Zhou , Y. Liu , X. Tan , Environ. Int. 2022, 158, 106980.

[67] S. Abdpour , L. Rademacher , M. N. A. Fetzer , T. H. Y. Beglau , C. Janiak , Solids, 2023, 4, 181.

[68] A. Heuer‐Jungemann , N. Feliu , I. Bakaimi , M. Hamaly , A. Alkilany , I. Chakraborty , A. Masood , M. F. Casula , A. Kostopoulou , E. Oh , K. Susumu , M. H. Stewart , I. L. Medintz , E. Stratakis , W. J. Parak , A. G. Kanaras , Chem. Rev. 2019, 119, 4819. 30920815 10.1021/acs.chemrev.8b00733

[69] L. Tan , B. Liu , K. Siemensmeyer , U. Glebe , A. Böker , Polym. Basel 2018, 10, 1053. 10.3390/polym10101053 PMC6404081 30960978

[70] F. Liu , J. Zhu , W. Yang , Y. Dong , Y. Hou , C. Zhang , H. Yin , S. Sun , Angew. Chem. Int. Ed. 2014, 53, 2176. 10.1002/anie.201309723 24453167

[71] S. Sun , C. B. Murray , D. Weller , L. Folks , A. Moser , Sci. 2000, 287, 1989. 10.1126/science.287.5460.1989 10720318

[72] J. Bai , J.‐P. Wang , Appl. Phys. Lett. 2005, 87, 152502.

[73] W. S. Seo , J. H. Lee , X. Sun , Y. Suzuki , D. Mann , Z. Liu , M. Terashima , P. C. Yang , M. V. McConnell , D. G. Nishimura , H. Dai , Nat. Mater. 2006, 5, 971. 17115025 10.1038/nmat1775

[74] M. G. Montiel Schneider , M. J. Martín , J. Otarola , E. Vakarelska , V. Simeonov , V. Lassalle , M. Nedyalkova , Pharm. 2022, 14, 204. 10.3390/pharmaceutics14010204 PMC8780449 35057099

[75] Y. Shi , M. Lin , X. Jiang , S. Liang , J. Nanomater. 2015, 2015, 467873.

[76] Y.‐L. Hou , S. Gao , Journal of Alloys and Compounds 2004, 365, 112.

[77] Y. Zeng , R. Hao , B. Xing , Y. Hou , Z. Xu , Chem. Commun. 2010, 46, 3920. 10.1039/c0cc00246a 20428516

[78] Y. Hou , H. Kondoh , M. Shimojo , T. Kogure , T. Ohta , J. Phys. Chem. B 2005, 109, 19094. 16853462 10.1021/jp0521149

[79] C. Yang , H. Zhao , Y. Hou , D. Ma , J. Am. Chem. Soc. 2012, 134, 15814. 22938192 10.1021/ja305048p

[80] J. Zhu , J. Wu , F. Liu , R. Xing , C. Zhang , C. Yang , H. Yin , Y. Hou , Nanoscale 2013, 5, 9141. 23913136 10.1039/c3nr02911e

[81] Z. Xu , Y. Hou , S. Sun , J. Am. Chem. Soc. 2007, 129, 8698. 17590000 10.1021/ja073057v

[82] C. Caro F. Gámez , P. Quaresma , J. M. Páez‐Muñoz , A. Domínguez , J. R. Pearson , M. Pernía Leal , A. M. Beltrán , Y. Fernandez‐Afonso , J. M. De la Fuente , R. Franco , E. Pereira , M. L. García‐Martín , Pharm. 2021, 13, 416. 10.3390/pharmaceutics13030416 PMC8003746 33804636

[83] N. Duan , S. Qi , Y. Guo , W. Xu , S. Wu , Z. Wang , LWT 2020, 134, 110017.

[84] E. Kozenkova , K. Levada , M. V. Efremova , A. Omelyanchik , Y. A. Nalench , A. S. Garanina , S. Pshenichnikov , D. G. Zhukov , O. Lunov , M. Lunova , I. Kozenkov , C. Innocenti , M. Albino , M. A. Abakumov , C. Sangregorio , V. Rodionova , Nanomater. Basel 2020, 10, 1646. 10.3390/nano10091646 PMC7558883 32825748

[85] J. Wu , Y. Hou , S. Gao , Nano Res. 2011, 4, 836.

[86] L. Yu , C. Yang , Y. Hou , Nanoscale 2014, 6, 10638. 25088826 10.1039/c4nr02163k

[87] M. Cialone , F. Celegato , M. Coïsson , G. Barrera , G. Fiore , R. Shvab , U. Klement , P. Rizzi , P. Tiberto , Sci Rep 2017, 7, 16691. 29192271 10.1038/s41598-017-16963-5 PMC5709423

[88] A. Akbarzadeh , M. Samiei , S. Davaran , Nanoscale Res. Lett. 2012, 7, 144. 22348683 10.1186/1556-276X-7-144 PMC3312841

[89] I. Castellanos‐Rubio , O. Arriortua , D. Iglesias‐Rojas , A. Barón , I. Rodrigo , L. Marcano , J. S. Garitaonandia , I. Orue , M. L. Fdez‐Gubieda , M. Insausti , Chem. Mater. 2021, 33, 8693. 34853492 10.1021/acs.chemmater.1c02654 PMC8619619

[90] Y. Hashimoto , M. Taguchi , S. Fukami , H. Momono , T. Matsushita , H. Matsuda , F. Matsui , H. Daimon , Surf. Interface Anal. 2019, 51, 115.

[91] Ö. S. Pekdur , S. Ö. Yıdırım , Z. Büyükmumcu , J. Mol. Struct. 2020, 1222, 128895.

[92] P. Soltanpour , R. Naderali , K. Mabhouti , Sci. Rep. 2024, 14, 21287. 39266615 10.1038/s41598-024-72026-6 PMC11393359

[93] Y. Hadadian , H. Masoomi , A. Dinari , C. Ryu , S. Hwang , S. Kim , B. K. Cho , J. Y. Lee , J. Yoon , ACS Omega 2022, 7, 15996. 35571799 10.1021/acsomega.2c01136 PMC9097206

[94] S. Angela , M. Ludmila , I. Cornelia‐Ioana , F. Denisa , C. Cristina , P. Natalia , G. Sabina , M. Mateusz , K. Joanna , S. Adrian‐Vasile , T. Doina Roxana , O. Ovidiu Cristian , F. Anton , Sci. Rep. 2024, 14, 26228. 39482399 10.1038/s41598-024-76552-1 PMC11528115

[95] J. K. Patra , K.‐H. Baek , J. Photochem. Photobiol. B 2017, 173, 291. 28623821 10.1016/j.jphotobiol.2017.05.045

[96] I. Ahmad , M. A. Aftab , A. Fatima , S. D. Mekkey , S. Melhi , S. Ikram , Coord. Chem. Rev. 2024, 514, 215904.

[97] T. S. Natarajan , P. K. Gopi , K. Natarajan , H. C. Bajaj , R. J. Tayade , Water‐Energy Nexus 2021, 4, 103.

[98] M. Cohen‐Erner , R. Khandadash , R. Hof , O. Shalev , A. Antebi , A. Cyjon , D. Kanakov , A. Nyska , G. Goss , J. Hilton , D. Peer , ACS Appl. Nano Mater. 2021, 4, 11187.

[99] M. D. Nguyen , H.‐V. Tran , S. Xu , T. R. Lee , Appl. Sci. Basel 2021, 11, 11301. 35844268 10.3390/app112311301 PMC9285867

[100] U. S. Ezealigo , B. N. Ezealigo , S. O. Aisida , F. I. Ezema , JCIS Open 2021, 4, 100027.

[101] E. Bianchetti , C. Di Valentin , J. Phys. Chem. Lett. 2022, 13, 9348. 36190176 10.1021/acs.jpclett.2c02186 PMC9575150

[102] M. G. Santos , D. T. de Carvalho , L. B. Caminitti , B. B. A. de Lima , M. H. da Silva Cavalcanti , D. F. R. dos Santos , L. S. Virtuoso , D. B. Hirata , E. C. Figueiredo , Food Chem. 2021, 353, 129442. 33714116 10.1016/j.foodchem.2021.129442

[103] P. Farinha , J. M. P. Coelho , C. P. Reis , M. M. Gaspar , Nanomater. Basel 2021, 11, 3432. 10.3390/nano11123432 PMC8706278 34947781

[104] A. Mittal , I. Roy , S. Gandhi , Magnetochemistry, 2022, 8, 107.

[105] V. F. Cardoso , A. Francesko , C. Ribeiro , M. Bañobre‐López , P. Martins , S. Lanceros‐Mendez , Adv. Healthcare Mater. 2018, 7, 1700845. 10.1002/adhm.201700845 29280314

[106] A. Saravanan , P. S. Kumar , M. Govarthanan , C. S. George , S. Vaishnavi , B. Moulishwaran , S. P. Kumar , S. Jeevanantham , P. R. Yaashikaa , Chemosphere 2021, 267, 129226. 33338712 10.1016/j.chemosphere.2020.129226

[107] A. Ahmed , E. Kim , S. Jeon , J.‐Y. Kim , H. Choi , Adv. Therapeutics 2022, 5, 2100237.

[108] M. Peiravi , H. Eslami , M. Ansari , H. Zare‐Zardini , J. Indian Chem. Soc. 2022, 99, 100269.

[109] M. Vassallo , D. Martella , G. Barrera , F. Celegato , M. Coïsson , R. Ferrero , E. S. Olivetti , A. Troia , H. Sözeri , C. Parmeggiani , D. S. Wiersma , P. Tiberto , A. Manzin , ACS Omega 2023, 8, 2143. 36687092 10.1021/acsomega.2c06244 PMC9850460

[110] Z. W. Tay , P. Chandrasekharan , A. Chiu‐Lam , D. W. Hensley , R. Dhavalikar , X. Y. Zhou , E. Y. Yu , P. W. Goodwill , B. Zheng , C. Rinaldi , S. M. Conolly , ACS Nano 2018, 12, 3699. 29570277 10.1021/acsnano.8b00893 PMC6007035

[111] A. Cabral‐Prieto , I. García‐Sosa , E. Reguera , N. N. Entzana , H. Tadeo‐Huerta , R. Ramírez‐Suárez , AIP Adv. 2025, 15, 045306.

[112] S. E. Sandler , B. Fellows , O. T. Mefford , Anal. Chem. 2019, 91, 14159. 31566353 10.1021/acs.analchem.9b03518

[113] J. B. Mamani , J. P. Borges , A. M. Rossi , L. F. Gamarra , Pharm. 2023, 15, 1663. 10.3390/pharmaceutics15061663 PMC10305209 37376111

[114] P. Pradhan , J. Giri , G. Samanta , H. D. Sarma , K. P. Mishra , J. Bellare , R. Banerjee , D. Bahadur , J. Biomed. Mater. Res., Part B 2007, 81B, 12. 10.1002/jbm.b.30630 16924619

[115] X. L. Liu , H. M. Fan , J. B. Yi , Y. Yang , E. S. G. Choo , J. M. Xue , D. D. Fan , J. Ding , J. Mater. Chem. 2012, 22, 8235.

[116] S. Bae , S. W. Lee , Y. Takemura , E. Yamashita , J. Kunisaki , S. Zurn , C. S. Kim , IEEE Transactions on Magnetics 2006, 42, 3566.

[117] C. L. Dennis , A. J. Jackson , J. A. Borchers , R. Ivkov , A. R. Foreman , P. J. Hoopes , R. Strawbridge , Z. Pierce , E. Goerntiz , J. W. Lau , C. Gruettner , J. Phys. D: Appl. Phys. 2008, 41, 134020.

[118] N. Kawai , M. Futakuchi , T. Yoshida , A. Ito , S. Sato , T. Naiki , H. Honda , T. Shirai , K. Kohri , The Prostate 2008, 68, 784. 18302228 10.1002/pros.20740

[119] M. Lévy , C. Wilhelm , J.‐M. Siaugue , O. Horner , J.‐C. Bacri , F. Gazeau , J. Phys.: Condens. Matter 2008, 20, 204133. 21694262 10.1088/0953-8984/20/20/204133

[120] M. Xing , J. Mohapatra , J. Beatty , J. Elkins , N. K. Pandey , A. Chalise , W. Chen , M. Jin , J. P. Liu , J. Alloys Compd. 2021, 871, 159475.

[121] H. Gavilán , S. K. Avugadda , T. Fernández‐Cabada , N. Soni , M. Cassani , B. T. Mai , R. Chantrell , T. Pellegrino , Chem. Soc. Rev. 2021, 50, 11614. 34661212 10.1039/d1cs00427a

[122] O. S. Sánchez , T. Castelo‐Grande , P. A. Augusto , J. M. Compaña , D. Barbosa , Nanomater. 2021, 11, 1652. 10.3390/nano11071652 PMC8306292 34201717

[123] D.‐H. Kim , S.‐H. Lee , K.‐N. Kim , K.‐M. Kim , I.‐B. Shim , Y.‐K. Lee , J. Magn. Magn. Mater. 2005, 293, 320.

[124] K. Nojima , S. Ge , Y. Katayama , S. Ueno , K. Iramina , J. Appl. Phys. 2010, 107, 09B320.

[125] R. Sharma , C. J. Chen , J. Nanopart Res. 2009, 11, 671.

[126] J. L. Corchero , A. Villaverde , Trends in Biotechnol. 2009, 27, 468. 10.1016/j.tibtech.2009.04.003 19564057

[127] S. Deger , D. Boehmer , I. Türk , J. Roigas , V. Budach , S. A. Loening , Eur. Urol. 2002, 42, 147. 12160585 10.1016/s0302-2838(02)00277-4

[128] E. Myrovali , K. Papadopoulos , G. Charalampous , P. Kesapidou , G. Vourlias , T. Kehagias , M. Angelakeris , U. Wiedwald , ACS Omega 2023, 8, 12955. 37065034 10.1021/acsomega.2c05962 PMC10099415

[129] F. Radinekiyan , M. R. Naimi‐Jamal , R. Eivazzadeh‐Keihan , H. A. M. Aliabadi , M. S. Bani , S. Shojaei , A. Maleki , Carbohydr. Polym. Technol. Appl. 2024, 7, 100481.

[130] L. Choopani , H. A. M. Aliabadi , F. Ganjali , A. Kashtiaray , R. Eivazzadeh‐Keihan , A. Maleki , M. Salimibani , A. H. Karimi , N. Salehpour , M. Mahdavi , Carbohydr. Polym. Technol. Appl. 2024, 7, 100495.

[131] R. K. Gilchrist , R. Medal , W. D. Shorey , R. C. Hanselman , J. C. Parrott , C. B. Taylor , Ann. Surg. 1957, 146, 596. 13470751 10.1097/00000658-195710000-00007 PMC1450524

[132] J. Y. Lee , Y. R. Na , C. M. Na , P. W. Im , H. W. Park , M. K. Kim , Y. Kim , J. H. You , D. S. Kang , H. E. Moon , H. R. Park , M. G. Kim , P. Kim , S. H. Park , H. W. Youn , Y. D. Son , Y. Takemura , C. W. Song , D. Ling , Y. Piao , S. H. Paek , Theranostics 2025, 15, 2883. 40083938 10.7150/thno.103503 PMC11898280

[133] P. Kharey , M. Goel , Z. Husain , R. Gupta , D. Sharma , M. M. I. A. Palani , S. Gupta , Mater. Chem. Phys. 2023, 293, 126859.

[134] F. Ahmadpour , F. Ganjali , F. Radinekiyan , R. Eivazzadeh‐Keihan , M. Salimibani , H. Bahreinizad , M. Mahdavi , A. Maleki , RSC Adv. 2024, 14, 13676. 38665491 10.1039/d3ra08067f PMC11044123

[135] H. Alibakhshi , H. Esfahani , E. Sharifi , Ceramics Int. 2024, 50, 8017.

[136] M. Kaneko , H. Yamazaki , T. Ono , M. Horie , A. Ito , Cancer Sci. 2023, 114, 3750. 37409483 10.1111/cas.15895 PMC10475774

[137] A. Manohar , V. Vijayakanth , K. H. Kim , J. Alloys Compd. 2021, 886, 161276.

[138] A. Nasser , A. Qdemat , H. Unterweger , R. Tietze , X. Sun , J. Landers , J. Kopp , B. Wu , M.‐S. Appavou , A. Murmiliuk , E. P. Gilbert , O. Petracic , A. Feoktystov , Phys. Chem. Chem, Phys. 2024, 26, 24912. 39291756 10.1039/d4cp01735h

[139] Y. Chen , S. Hou , Cell Death Discov. 2023, 9, 1. 37380637 10.1038/s41420-023-01490-2 PMC10307851

[140] C. Chapa González , J. U. Navarro Arriaga , P. E. García Casillas , Polym., Polym. Compos. 2021, 29, S1009.

[141] N. Desai , AAPS J 2012, 14, 282. 22407288 10.1208/s12248-012-9339-4 PMC3326161

[142] S. Malik , K. Muhammad , Y. Waheed , Molecules 2023, 28, 6624.37764400

[143] L. Mödl , L. R. Carnell , R. Stein , A. Kerpes , F. Pfister , B. Wirthl , W. A. Wall , C. Alexiou , C. Janko , Front. Bioeng. Biotechnol. 2025, 12. 10.3389/fbioe.2024.1498120 PMC11750997 39845377

[144] J. Han , Y. Tian , M. Wang , Y. Li , J. Yin , W. Qu , C. Yan , R. Ding , Y. Guan , Q. Wang , Front. Pharmacol. 2022, 13. 10.3389/fphar.2022.1011065 PMC9512491 36172182

[145] M. M. Patton , A. Puzari , J. Borah , J. Magn. Magn. Mater. 2024, 599, 172062.

[146] N. Van Khien , C. Thi Anh Xuan , L. H. Nguyen , P. H. Nam , T. Thi Thao , Mater. Today Commun. 2024, 38, 107982.

[147] R. Das , N. Rinaldi‐Montes , J. Alonso , Z. Amghouz , E. Garaio , J. A. García , P. Gorria , J. A. Blanco , M. H. Phan , H. Srikanth , ACS Appl. Mater. Interfaces 2016, 8, 25162. 27589410 10.1021/acsami.6b09942

[148] K. Simeonidis , S. Liébana‐Viñas , U. Wiedwald , Z. Ma , Z.‐A. Li , M. Spasova , O. Patsia , E. Myrovali , A. Makridis , D. Sakellari , I. Tsiaoussis , G. Vourlias , M. Farle , M. Angelakeris , RSC Adv. 2016, 6, 53107. 10.1038/srep37934 PMC5126575 27897195

[149] A. Rezanezhad , A. Hajalilou , F. Eslami , E. Parvini , E. Abouzari‐Lotf , B. Aslibeiki , J Mater. Sci.: Mater. Electron. 2021, 32, 24026.

[150] R. R. Shah , T. P. Davis , A. L. Glover , D. E. Nikles , C. S. Brazel , J. Magn. Magn. Mater. 2015, 387, 96. 25960599 10.1016/j.jmmm.2015.03.085 PMC4422114

[151] P. Zhang , T. Han , H. Xia , L. Dong , L. Chen , L. Lei , Front Oncol 2022, 12, 836397. 35372087 10.3389/fonc.2022.836397 PMC8966402

[152] A. Ashkbar , F. Rezaei , F. Attari , S. Ashkevarian , Sci. Rep. 2020, 10, 21206. 33273672 10.1038/s41598-020-78241-1 PMC7713176

[153] M. Choppadandi , K. Soumya , S. Ghosh , A. Balu , T. Shingote , S. S. Babu , V. S. Prasanna , S. Arumugam , R. Velyutham , M. M. Yallapu , G. Kapusetti , Nanotheranostics 2024, 8, 442. 38961886 10.7150/ntno.91871 PMC11217784

[154] A. Farzin , S. A. Etesami , J. Quint , A. Memic , A. Tamayol , Adv. Healthc Mater. 2020, 9, e1901058. 32196144 10.1002/adhm.201901058 PMC7482193

[155] Z. Wang , F. Zhang , D. Shao , Z. Chang , L. Wang , H. Hu , X. Zheng , X. Li , F. Chen , Z. Tu , M. Li , W. Sun , L. Chen , W.‐F. Dong , Adv. Sci. 2019, 6, 1901690. 10.1002/advs.201901690 PMC6864517 31763151

[156] A. Urazaliyeva , P. Kanabekova , A. Beisenbayev , G. Kulsharova , T. Atabaev , S. Kim , C.‐K. Lim , Sci. Rep. 2024, 14, 17507. 39080400 10.1038/s41598-024-68077-4 PMC11289472

[157] H. S. Han , K. Y. Choi , Biomedicines 2021, 9, 305.33809691

[158] M. Overchuk , R. A. Weersink , B. C. Wilson , G. Zheng , ACS Nano 2023, 17, 7979. 37129253 10.1021/acsnano.3c00891 PMC10173698

[159] S. Wang , Y. Hou , J. Appl. Phys. 2021, 130, 070902.

[160] L. Zhang , G. Alimu , Z. Du , T. Yan , H. Li , R. Ma , Z. Lan , Z. Yu , N. Alifu , K. Sun , ACS Omega 2023, 8, 21793. 37360441 10.1021/acsomega.3c01374 PMC10286267

[161] J. Liu , X. Guo , Z. Zhao , B. Li , J. Qin , Z. Peng , G. He , D. J. L. Brett , R. Wang , X. Lu , Appl. Mater. Today 2020, 18, 100457.

[162] Z. Chu , Z. Wang , L. Chen , X. Wang , C. Huang , M. Cui , D.‐P. Yang , N. Jia , ACS Appl. Nano Mater. 2018, 1, 2332.

[163] J. Mohapatra , S. Nigam , J. George , A. C. Arellano , P. Wang , J. P. Liu , Mater. Today Phys. 2023, 32, 101003. 40740662 10.1016/j.mtphys.2023.101003 PMC12308503

[164] A. Espinosa , R. Di Corato , J. Kolosnjaj‐Tabi , P. Flaud , T. Pellegrino , C. Wilhelm , ACS Nano 2016, 10, 2436. 26766814 10.1021/acsnano.5b07249

[165] J. Yu , Y. Ju , L. Zhao , X. Chu , W. Yang , Y. Tian , F. Sheng , J. Lin , F. Liu , Y. Dong , Y. Hou , ACS Nano 2016, 10, 159. 26602632 10.1021/acsnano.5b04706

[166] J. F. Liu , B. Jang , D. Issadore , A. Tsourkas , Wiley Interdiscip. Rev.: Nanomed. Nanobiotechnol. 2019, 11, e1571. 31241251 10.1002/wnan.1571 PMC6788948

[167] A. Tewabe , A. Abate , M. Tamrie , A. Seyfu , E. A. Siraj , JMDH 2021, 14, 1711. 34267523 10.2147/JMDH.S313968 PMC8275483

[168] T. C. Ezike , U. S. Okpala , U. L. Onoja , C. P. Nwike , E. C. Ezeako , O. J. Okpara , C. C. Okoroafor , S. C. Eze , O. L. Kalu , E. C. Odoh , U. G. Nwadike , J. O. Ogbodo , B. U. Umeh , E. C. Ossai , B. C. Nwanguma , Heliyon 2023, 9, e17488. 37416680 10.1016/j.heliyon.2023.e17488 PMC10320272

[169] P. M. Price , W. E. Mahmoud , A. A. Al‐Ghamdi , L. M. Bronstein , Front. Chem. 2018, 6. 10.3389/fchem.2018.00619 PMC6297194 30619827

[170] M. Shapiro , Scilight 2023, 2023, 461102.

[171] M. M. Selim , S. El‐Safty , A. Tounsi , M. Shenashen , APL Materials 2024, 12, 010601.

[172] J. Kurczewska , B. Dobosz , Appl. Sci. 2024, 14, 1132.

[173] K. Ulbrich , K. Holá , V. Šubr , A. Bakandritsos , J. Tuček , R. Zbořil , Chem. Rev. 2016, 116, 5338. 27109701 10.1021/acs.chemrev.5b00589

[174] R. Al‐Obaidy , A. J. Haider , S. Al‐Musawi , N. Arsad , Sci. Rep. 2023, 13, 3180. 36823237 10.1038/s41598-023-30221-x PMC9950487

[175] K. T. Nguyen , G. Go , J. Zhen , M. C. Hoang , B. Kang , E. Choi , J.‐O. Park , C.‐S. Kim , Sci. Rep. 2021, 11, 15122. 34302003 10.1038/s41598-021-94446-4 PMC8302636

[176] S. Kaushik , J. Thomas , V. Panwar , P. Murugesan , V. Chopra , N. Salaria , R. Singh , H. S. Roy , R. Kumar , V. Gautam , D. Ghosh , Nanoscale 2022, 14, 1713. 35072191 10.1039/d1nr07435k

[177] S. M. B. Bataineh , I. M. Arafa , S. M. Abu‐Zreg , M. M. Al‐Gharaibeh , H. M. Hammouri , Y. H. Tarazi , H. Darmani , Magnetochemistry 2024, 10, 49.

[178] N. T. Tasnim , N. Ferdous , Md. M. H. Rumon , M. S. Shakil , ACS Omega 2024, 9, 16. 38222657 10.1021/acsomega.3c06323 PMC10785672

[179] M. M. Abdelaziz , A. Hefnawy , A. Anter , M. M. Abdellatif , M. A. F. Khalil , I. A. Khalil , ACS Omega 2022, 7, 30161. 36061717 10.1021/acsomega.2c03226 PMC9434613

[180] A. Allafchian , S. S. Hosseini , IET Nanobiotechnol. 2019, 13, 786. 31625518 10.1049/iet-nbt.2019.0146 PMC8676097

[181] J. Zúñiga‐Miranda , J. Guerra , A. Mueller , A. Mayorga‐Ramos , S. E. Carrera‐Pacheco , C. Barba‐Ostria , J. Heredia‐Moya , L. P. Guamán , Nanomater. 2023, 13, 2919. 10.3390/nano13222919 PMC10674528 37999273

[182] T.‐G. Zhang , C.‐Y. Miao , Nanomater. 2024, 14, 1311.

[183] I. Saba , K. M. Batoo , K. Wani , R. Verma , S. Hameed , Cureus 2024, 16, e69556. 39421116 10.7759/cureus.69556 PMC11484742

[184] E. Chung , G. Ren , I. Johnston , R. K. Matharu , L. Ciric , A. Walecka , Y.‐K. Cheong , Bioeng. Basel 2023, 10, 1259. 10.3390/bioengineering10111259 PMC10669044 38002383

[185] N. Banura , K. Murase , Jpn. J. Appl. Phys. 2017, 56, 088001.

[186] E. M. Sharaf , A. Hassan , F. A. AL‐Salmi , F. M. Albalwe , H. M. R. Albalawi , D. B. Darwish , E. Fayad , Front. Microbiol. 2022, 13, 10.3389/fmicb.2022.929491. PMC9478199 36118244

[187] R. M. Pinto , F. A. Soares , S. Reis , C. Nunes , P. Van Dijck , Front. Microbiol. 2020, 11, 10.3389/fmicb.2020.00952. PMC7264105 32528433

[188] F. Hassanzadeh‐Afruzi , Z. Amiri‐Khamakani , S. Bahrami , M. R. Ahghari , A. Maleki , Sci. Rep. 2022, 12, 4503. 35297399 10.1038/s41598-022-08318-6 PMC8927411

[189] S. Faisal , S. Sadiq , M. Mustafa , M. Hayat Khan , M. Sadiq , Z. Iqbal , M. Khan , RSC Sustainability 2023, 1, 139.

[190] M. Caciandone , A.‐G. Niculescu , V. Grumezescu , A. C. Bîrcă , I. C. Ghica , B. Ştefan Vasile , O. Oprea , I. C. Nica , M. S. Stan , A. M. Holban , A. M. Grumezescu , I. Anghel , A. G. Anghel , Antibiot. Basel 2022, 11, 623. 10.3390/antibiotics11050623 PMC9137518 35625267

[191] H. J. Imran , K. A. Hubeatir , K. A. Aadim , Sci. Rep. 2023, 13, 5441. 37012294 10.1038/s41598-023-32330-z PMC10070463

[192] A. M. Grumezescu , R. Cristescu , M. C. Chifiriuc , G. Dorcioman , G. Socol , I. N. Mihailescu , D. E. Mihaiescu , A. Ficai , O. R. Vasile , M. Enculescu , D. B. Chrisey , Biofabrication 2015, 7, 015014. 25797361 10.1088/1758-5090/7/1/015014

[193] A. M. El‐Khawaga , M. Ayman , O. Hafez , R. E. Shalaby , Sci. Rep. 2024, 14, 12877. 38834648 10.1038/s41598-024-62868-5 PMC11150482

[194] M. Agarwala , B. Choudhury , R. N. S. Yadav , Indian J. Microbiol. 2014, 54, 365. 24891746 10.1007/s12088-014-0462-z PMC4039716

[195] J. Borcherding , J. Baltrusaitis , H. Chen , L. Stebounova , C.‐M. Wu , G. Rubasinghege , I. A. Mudunkotuwa , J. C. Caraballo , J. Zabner , V. H. Grassian , A. P. Comellas , Environ. Sci.: Nano 2014, 1, 123. 25221673 10.1039/C3EN00029J PMC4158920

[196] C. Haney , J. J. Rowe , J. B. Robinson , J. Biomater. Nanobiotechnol. 2012, 3, 508.

[197] Z. Vardanyan , A. Trchounian , Biochem. Biophys. Res. Commun. 2012, 417, 541. 22166211 10.1016/j.bbrc.2011.11.159

[198] T. Javanbakht , S. Laurent , D. Stanicki , K. J. Wilkinson , PLOS ONE 2016, 11, e0154445. 27115356 10.1371/journal.pone.0154445 PMC4845983

[199] H. Wu , J.‐J. Yin , W. G. Wamer , M. Zeng , Y. M. Lo , J. Food Drug Anal. 2014, 22, 86. 24673906 10.1016/j.jfda.2014.01.007 PMC9359154

[200] P. Dames , B. Gleich , A. Flemmer , K. Hajek , N. Seidl , F. Wiekhorst , D. Eberbeck , I. Bittmann , C. Bergemann , T. Weyh , L. Trahms , J. Rosenecker , C. Rudolph , Nat. Nanotech 2007, 2, 495. 10.1038/nnano.2007.217 18654347

[201] D. D. Dantzger , M. Dantzger , C. M. Jonsson , H. Aoyama , Water, Air, Soil Pollut. 2017, 228, 391.

[202] A. Hidangmayum , A. Debnath , A. Guru , B. N. Singh , S. K. Upadhyay , P. Dwivedi , Int. J. Environ. Sci. Technol. 2023, 20, 11693. 10.1007/s13762-022-04560-7 PMC9521565 36196301

[203] J. Allan , S. Belz , A. Hoeveler , M. Hugas , H. Okuda , A. Patri , H. Rauscher , P. Silva , W. Slikker , B. Sokull‐Kluettgen , W. Tong , E. Anklam , Regul. Toxicol. Pharmacol. 2021, 122, 104885. 33617940 10.1016/j.yrtph.2021.104885 PMC8121750

[204] A. Mech , S. Gottardo , V. Amenta , A. Amodio , S. Belz , S. Bøwadt , J. Drbohlavová , L. Farcal , P. Jantunen , A. Małyska , K. Rasmussen , J. Riego Sintes , H. Rauscher , Regul. Toxicol. Pharmacol. 2022, 128, 105093. 34864125 10.1016/j.yrtph.2021.105093 PMC8795056

[205] E. S. Okeke , T. P. C. Ezeorba , G. Mao , Y. Chen , W. Feng , X. Wu , Environ. Pollut. 2022, 295, 118722. 34952184 10.1016/j.envpol.2021.118722

[206] A. V. Singh , A. Shelar , M. Rai , P. Laux , M. Thakur , I. Dosnkyi , G. Santomauro , A. K. Singh , A. Luch , R. Patil , J. Bill , J. Agric. Food Chem. 2024, 72, 2835. 38315814 10.1021/acs.jafc.3c06466

[207] Y. S. El‐Temsah , A. Sevcu , K. Bobcikova , M. Cernik , E. J. Joner , Chemosphere 2016, 144, 2221. 26598990 10.1016/j.chemosphere.2015.10.122

[208] M. Rui , C. Ma , Y. Hao , J. Guo , Y. Rui , X. Tang , Q. Zhao , X. Fan , Z. Zhang , T. Hou , S. Zhu , Front. Plant Sci. 2016, 7, 10.3389/fpls.2016.00815. PMC4899443 27375665

[209] S. Mishra , C. Keswani , P. C. Abhilash , L. F. Fraceto , H. B. Singh , Front. Plant Sci. 2017, 8. 10.3389/fpls.2017.00471. PMC5378785 28421100

[210] J. Gamonchuang , Y. Santaladchaiyakit , R. Burakham , ACS Omega 2022, 7, 12202. 35449973 10.1021/acsomega.2c00596 PMC9016810

[211] L. Yang , P. Su , X. Chen , R. Zhang , Y. Yang , Anal. Methods 2015, 7, 3246.

[212] T. H. Thi Nguyen , K. Thuy Nguyen , B. Hung Le , X. Truong Nghiem , D. Duong La , D. Khiem Nguyen , H. P. Thi Nguyen , RSC Adv. 2024, 14, 22304. 39010918 10.1039/d4ra03567d PMC11247437

[213] Md. Nuruzzaman , M. M. Rahman , Y. Liu , R. Naidu , J. Agric. Food Chem. 2016, 64, 1447. 26730488 10.1021/acs.jafc.5b05214

[214] M. Kah , S. Beulke , K. Tiede , T. Hofmann , Crit. Rev. Environ. Sci. Technol. 2013, 43, 1823.

[215] R. Grillo , N. Z. P. dos Santos , C. R. Maruyama , A. H. Rosa , R. de Lima , L. F. Fraceto , J. Hazard. Mater. 2012, 231–232, 1. 10.1016/j.jhazmat.2012.06.019 22795586

[216] V. Takeshita , L. B. Carvalho , J. A. Galhardi , G. V. Munhoz‐Garcia , R. F. Pimpinato , H. C. Oliveira , V. L. Tornisielo , L. F. Fraceto , ACS Nanosci. Au 2022, 2, 307. 37102067 10.1021/acsnanoscienceau.1c00055 PMC10125138

[217] A. Sikder , A. K. Pearce , S. J. Parkinson , R. Napier , R. K. O’Reilly , ACS Appl. Polym. Mater. 2021, 3, 1203.

[218] P. M. Kopittke , E. Lombi , P. Wang , J. K. Schjoerring , S. Husted , Environ. Sci.: Nano 2019, 6, 3513.

[219] K. S. V. P. Chandrika , A. A. Qureshi , A. Singh , C. Sarada , B. Gopalan , ACS Omega 2022, 7, 45481. 36530273 10.1021/acsomega.2c06096 PMC9753186

[220] D. T. Clarkson , H. Marschner , Ann. Bot. 1995 1996, 78, 527.

[221] R. Liu , H. Zhang , R. Lal , Water, Air, Soil Pollut. 2016, 227, 42.

[222] M. H. Ghafariyan , M. J. Malakouti , M. R. Dadpour , P. Stroeve , M. Mahmoudi , Environ. Sci. Technol. 2013, 47, 10645. 23951999 10.1021/es402249b

[223] J. Li , J. Hu , C. Ma , Y. Wang , C. Wu , J. Huang , B. Xing , Chemosphere 2016, 159, 326. 27314633 10.1016/j.chemosphere.2016.05.083

[224] S. Palchoudhury , K. L. Jungjohann , L. Weerasena , A. Arabshahi , U. Gharge , A. Albattah , J. Miller , K. Patel , R. A. Holler , RSC Adv. 2018, 8, 24075. 35539206 10.1039/c8ra04680h PMC9081864

[225] L. Zhao , L. Lu , A. Wang , H. Zhang , M. Huang , H. Wu , B. Xing , Z. Wang , R. Ji , J. Agric. Food Chem. 2020, 68, 1935. 32003987 10.1021/acs.jafc.9b06615

[226] M. Ju , M. Navarreto‐Lugo , S. Wickramasinghe , N. B. Milbrandt , A. McWhorter , A. C. S. Samia , Nanoscale 2019, 11, 18582. 31528944 10.1039/c9nr05477d

[227] A. Spanos , K. Athanasiou , A. Ioannou , V. Fotopoulos , T. Krasia‐Christoforou , Nanomater. 2021, 11, 3106. 10.3390/nano11113106 PMC8623625 34835870

[228] Y. Liu , L. Yue , Z. Wang , B. Xing , Environ. Chem. 2019, 16, 430.

[229] T. A. Swift , T. A. A. Oliver , M. C. Galan , H. M. Whitney , Interface Focus 2018, 9, 20180048. 30603068 10.1098/rsfs.2018.0048 PMC6304006

[230] S. K. Sonawane , S. S. Arya , J. G. J. Leblanc , N. Jha , Use of Nanomaterials in the Detection of Food Contaminants, 2014, https://ri.conicet.gov.ar/handle/11336/2904 (accessed: December 2024).

[231] F. Speroni , L. Elviri , M. Careri , A. Mangia , Anal. Bioanal. Chem. 2010, 397, 3035. 20607526 10.1007/s00216-010-3851-0

[232] K. Yang , D. M. Jenkins , W. W. Su , J. Microbiol. Methods 2011, 86, 69. 21473888 10.1016/j.mimet.2011.03.018

[233] R. Khan , A. Rehman , A. Hayat , S. Andreescu , Magnetochemistry 2019, 5, 63.

[234] M. Varshney , Y. Li , Biosens. Bioelectron. 2007, 22, 2408. 17045791 10.1016/j.bios.2006.08.030

[235] S. Targuma , P. B. Njobeh , P. G. Ndungu , Molecules 2021, 26, 4284. 34299560 10.3390/molecules26144284 PMC8303358

[236] B. S. Inbaraj , B. H. Chen , J. Food Drug Anal. 2015, 24, 15. 28911398 10.1016/j.jfda.2015.05.001 PMC9345428

[237] J. Zhuang , R. W. Gentry , Biotechnology And Nanotechnology Risk Assessment: Minding And Managing The Potential Threats Around Us, American Chemical Society 2011, pp. 41–67.

[238] T. P. Fato , D.‐W. Li , L.‐J. Zhao , K. Qiu , Y.‐T. Long , ACS Omega 2019, 4, 7543. 31459847 10.1021/acsomega.9b00731 PMC6648574

[239] X. Huang , X. Wang , X. Liu , L. Cheng , J. Pan , X. Yang , Plants 2024, 13, 1395. 38794464 10.3390/plants13101395 PMC11124989

[240] R. Sharma , V. Kumar , Waste Management Bulletin 2024, 2, 152.

[241] B. Nandini , K. S. Mawale , P. Giridhar , Biotech 2023, 13, 73. 10.1007/s13205-023-03470-w PMC9898490 36748014

[242] K. Mgadi , B. Ndaba , A. Roopnarain , H. Rama , R. Adeleke , Front Microbiol. 2024, 15, 1354440. 38511012 10.3389/fmicb.2024.1354440 PMC10951078

[243] K. Wu , J.‐P. Wang , N. A. Natekar , S. Ciannella , C. González‐Fernández , J. Gomez‐Pastora , Y. Bao , J. Liu , S. Liang , X. Wu , L. Nguyen , T. Tran , K. Mercedes Paz González , H. Choe , J. Strayer , P. R. Iyer , J. Chalmers , V. K. Chugh , B. Rezaei , S. Mostufa , Z. W. Tay , C. Saayujya , Q. Huynh , J. Bryan , R. Kuo , E. Yu , P. Chandrasekharan , B. Fellows , S. Conolly , R. L. Hadimani , et al., Nanotechnology 2025, 36, 042003.

[244] A. K. Mittal , Y. Chisti , U. C. Banerjee , Biotechnol. Adv. 2013, 31, 346. 23318667 10.1016/j.biotechadv.2013.01.003

[245] S. Shukla , R. Khan , A. Daverey , Environ. Technol. Innovation 2021, 24, 101924.

[246] G. R. Rodrigues , C. López‐Abarrategui , I. de la Serna Gómez , S. C. Dias , A. J. Otero‐González , O. L. Franco , Int. J. Pharm. 2019, 555, 356. 30453018 10.1016/j.ijpharm.2018.11.043

[247] N. Tarannum , A. Gautam , T. Chauhan , D. Kumar , Sens. Technol. 2024, 2, 2373196.

[248] E. S. Vasquez , J. M. Feugang , S. T. Willard , P. L. Ryan , K. B. Walters , J. Nanobiotechnol. 2016, 14, 20. 10.1186/s12951-016-0168-y PMC4794913 26984640

[249] A. Baki , F. Wiekhorst , R. Bleul , Bioeng. Basel 2021, 8, 134. 10.3390/bioengineering8100134 PMC8533261 34677207

[250] D. D. Stueber , J. Villanova , I. Aponte , Z. Xiao , V. L. Colvin , Pharm. 2021, 13, 943. 10.3390/pharmaceutics13070943 PMC8309177 34202604

[251] X. Wang , T. Yang , Q. Li , Responsive Mater. 2024, 2, e20240027.

[252] C. Alexiou , R. Jurgons , C. Seliger , H. Iro , J. Nanosci. Nanotechnol. 2006, 6, 2762. 17048480 10.1166/jnn.2006.464

[253] A. J. Giustini , A. A. Petryk , S. M. Cassim , J. A. Tate , I. Baker , P. J. Hoopes , Nano Life 2010, 1, 10.1142/S1793984410000067. PMC3859910 24348868

[254] J. Hong , L. Wang , Q. Zheng , C. Cai , X. Yang , Z. Liao , Mater. 2024, 17, 2870. 10.3390/ma17122870 PMC11204782 38930238

[255] S. Konnova , E. Rozhina , Int. J. Mol. Sci. 2024, 25, 5847. 38892034 10.3390/ijms25115847 PMC11172554

[256] D. D. Stueber , J. Villanova , I. Aponte , Z. Xiao , V. L. Colvin , Pharm. 2021, 13, 943. 10.3390/pharmaceutics13070943 PMC8309177 34202604

[257] M. Latorre , C. Rinaldi , P R Health Sci. J. 2009, 28, 227. 19715115

[258] N. Korkmaz , R. İmamoğlu , A. Karadağ , E. Şahin Yıldırım , Y. Ceylan , F. Şen ,ChemistryOpen 2025, 2500189, 10.1002/open.202500189. PMC12598807 40525672

[259] C. Miguel‐Rojas , A. Pérez‐de‐Luque , Emerg Top. Life Sci. 2023, 7, 229. 37921102 10.1042/ETLS20230070 PMC10754331

[260] A. Ali , H. Zafar , M. Zia , I. ul Haq , A. R. Phull , J. S. Ali , A. Hussain , Nanotechnol. Sci. Appl. 2016, 9, 49. 27578966 10.2147/NSA.S99986 PMC4998023

[261] F. Aslani , S. Bagheri , N. Muhd Julkapli , A. S. Juraimi , F. S. G. Hashemi , A. Baghdadi , Sci. World J. 2014, 2014, 641759. 10.1155/2014/641759 PMC4150468 25202734

[262] M. Wang , L. Jin , P. Hang‐Mei Leung , F. Wang‐Ngai Chow , X. Zhao , H. Chen , W. Pan , H. Liu , S. Li , Front. Bioeng. Biotechnol. 2024, 12, 10.3389/fbioe.2024.1393789. PMC11079239 38725992

[263] S. Delshadi , G. Blaire , P. Kauffmann , M. Fratzl , T. Devillers , D. Delabouglise , M. Weidenhaupt , N. M. Dempsey , O. Cugat , F. Bruckert , P. N. Marche , Bioanalysis 2017, 9, 517. 10.4155/bio-2016-0232 28225302

[264] R. Abedini‐Nassab , M. Pouryosef Miandoab , M. Şaşmaz , Micromachines Basel 2021, 12, 768. 34210058 10.3390/mi12070768 PMC8306075

[265] B. Tian , T. Z. G. de la Torre , M. Donolato , M. F. Hansen , P. Svedlindh , M. Strömberg , Anal. Methods 2016, 8, 5009.

[266] M. Jiang , L. Zhang , F. Wang , J. Zhang , G. Liu , B. Gao , D. Wei , Sci. Rep. 2017, 7, 13329. 29042612 10.1038/s41598-017-13648-x PMC5645317

[267] A. Dasari , J. Xue , S. Deb , Nanomater. Basel 2022, 12, 757. 10.3390/nano12050757 PMC8911835 35269245

[268] A. R. Abdel Fattah , N. Kolaitis , K. Van Daele , B. Daza , A. G. Rustandi , A. Ranga , Nat. Commun. 2023, 14, 5281. 37644160 10.1038/s41467-023-41037-8 PMC10465512

[269] J. Xue , N. Gurav , S. Elsharkawy , S. Deb , ACS Appl. Bio Mater. 2024, 7, 168. 10.1021/acsabm.3c00732 PMC10792668 38109842

[270] T. S. S. Carvalho , P. M. C. Torres , J. H. Belo , J. Mano , S. M. Olhero , Adv. NanoBiomed Res. 2023, 3, 2300035.

[271] T. Nguyen , J. Gao , P. Wang , A. Nagesetti , P. Andrews , S. Masood , Z. Vriesman , P. Liang , S. Khizroev , X. Jin , Neurotherapeutics 2021, 18, 2091. 34131858 10.1007/s13311-021-01071-0 PMC8609092

[272] S. D. Kong , J. Lee , S. Ramachandran , B. P. Eliceiri , V. I. Shubayev , R. Lal , S. Jin , J Control Release 2012, 164, 49. 23063548 10.1016/j.jconrel.2012.09.021 PMC4440873

[273] R. Qiao , C. Fu , H. Forgham , I. Javed , X. Huang , J. Zhu , A. K. Whittaker , T. P. Davis , Adv. Drug Delivery Rev. 2023, 197, 114822. 10.1016/j.addr.2023.114822 37086918

[274] M. Wankhede , A. Bouras , M. Kaluzova , C. G. Hadjipanayis , Expert Rev. Clin. Pharmacol. 2012, 5, 173. 22390560 10.1586/ecp.12.1 PMC3461264

[275] H. Zhou , C. C. Mayorga‐Martinez , S. Pané , L. Zhang , M. Pumera , Chem. Rev. 2021, 121, 4999. 33787235 10.1021/acs.chemrev.0c01234 PMC8154323

[276] C. Wang , Z. Zhao , J. Han , A. A. Sharma , H. Wang , X. S. Zhang , Adv. Sci. Weinh 2024, 11, 2308619. 39041885 10.1002/advs.202308619 PMC11425225

