# Supplementary material for: Magnetic Nanoparticles: Synthesis and Applications in Life Sciences
Source: ChemistryOpen. 2025 Jul 29;14(12):e202500214. doi: 10.1002/open.202500214 (PMC12680574; doi:10.1002/open.202500214)
Supplement: Supplementary file 1 — Supplementary Material [file OPEN-14-e202500214-s001.pdf]

Supplementary data

Magnetic Nanoparticles: Synthesis and Applications in Life Sciences

Kishore Chand[a], Erick S. Vasquez-Guardado[a,*]

a] Kishore Chand
Department of Chemical and Materials Engineering
University of Dayton
300 college park, Dayton, OH 45469-0256, USA.
E-mail: chandk1@udayton.edu

[b] *Erick S. Vasquez-Guardado
Department of Chemical and Materials Engineering
University of Dayton
300 college park, Dayton, OH 45469-0256, USA.
E-mail: evasquez1@udayton.edu

Table S1. SAR values obtained from the different kinds of nanocomposites reported in recent years by using the co-precipitation method

Entry	Magnetic nanocomposites	Synthesis method	Concentration (mg/mL)	Frequency(kHz)	SAR (W/g)	Saturation Magnetization (emu/g)	Reference
4	Fe_3O_4	Co precipitation method	5	765.85	145.84	70.37	[1]
5	Iron Oxide (Fe_3O_4)	Co precipitation method	2.5	332.8	261	Not specified	[2]
6	Fe_3O_4/ HNTs	Co-precipitation	0.5, 1, and 2	400 and 200	94 at 400 kHz and 53 at 200 kHz	73.84 for Fe_3O_4 and 30.63 for Fe_3O_4/ HNTs	[3]
9	Fe_3O_4-Pectigel	Co-precipitation	3.2	276	79	51	[4]
10	Fe_3O_4-MNPs	Co-precipitation	2.5, 5, and 10	Not specified	261.21, 163.42, and 84.28 W/g	Not specified	[2]
13	Fe_3O_4/Bentonite	Co-precipitation	0.5, 1, and 2	400	83	48.84	[5]
14	Fe_3O_4/ZNS	Co-precipitation	2, 4, and 6	336	52.84, 34.98 and 24.21 for Fe_3O_4, and 79.69 , 46.12 , and 31.72 for Fe_3O_4/ZNS	Saturation magnetization value of pure Fe_3O_4 is 50.10 , while for Fe_3O_4/ZnS nanocomposites it is 14.01.	[6]

24	Fe_3O_4@Pectin	Coprecipitation method	0.5	400	214	33	[7]
26	Fe_3O_4 with peppermint	Coprecipitation method	9	92	21.27	52	[8]
12	Fe_3O_4@γ-Fe_2O_3	Co-precipitation method	0.1	570	118	70.84	[9]

Table S2. SAR values obtained from the different kinds of nanocomposites reported in recent years by using different synthesis methods

**Entry**	**Magnetic nanocomposites**	**Synthesis method**	**Concentration (mg/mL)**	**Frequency(kHz)**	**SAR (W/g)**	**Saturation Magnetization (emu/g)**	**Reference**
1	Mn Fe_3O_4	Not specified	20	100-200	250–400	Not specified	[10]
2	Fe_3O_4	Not specified	1	Not specified	426	89	[11]
25	Fe_3O_4 -Au using leaf extract (Diocia)	Green synthesis	0.5	405	+-4.5	65	[12]
3	$MnFe_2O_4$	Flame spray pyrolysis	5	592.2	High	Not specified	[13]
7	magnetic nanocomposite of SA-TA hydrogel/SF/ Fe_3O_4	Ionic cross linking	2	300	73.53	0.90	[14]
27	$Co_{0.4}Zn_{0.}6Fe_2O_4$	Green method	Not specified	100	2.56	Not specified	[15]
28	Citric acid coated $CoFe_2O_4$ NPs (CFO@CA NPs) and other $CoFe_2O_4$ nanocomposites	Microwave assisted, Coprecipitation method and Hydrothermal method	Not specified	316	142	Not specified	[16]
11	Fe_3O_4@Ag	Green synthetic method	Not specified	15	1.25	43.2	[17]
16	Fe_3O_4 -peel<br>Fe_3O_4-pupl	Green synthetic method	0.55 and 0.15	100	0.48 for Fe_3O_4 -peel and 1.33 for Fe_3O_4 -pupl	Not specified	[18]
17	Fe_3O_4@Ag	Hydrothermal-Co-precipitation	The $AgNO_3$ used were in the following concentrations:1.2 g/L and 0.22 g/L which change to 1200 mg/mL and 220 mg/mL	274	43	56	[19]

			respectively.				
18	TREG coated $CuFe_2O_4$	Solvethermal	Not specified	120	44.9	Not specified	[20]
19	Fe_3O_4-alginate/PVA	In-situ cross-linking and synthesis of magnetic nanocomposite hydrogels.	1	72.42	49.27	9	[21]
20	Magnetic alginate-tannic acid hydrogel embedded with silk fibroin	Cross linking, sonication, In-Situ synthesis	5	100-400	22.3	15.96	[22]
21	Magnetic solid lipid NPs (mSLNs) and superparamagnetic iron oxide NPs (SPIONs)	Coprecipitation and Emulsification method	100 µg/mL	5-10	200 and 450	67	[23]
22	Super paramagnetic IONCs and nanocomposies	Thermal deposition	1	587.1	2524.25	63.74	[24]
23	CS hydrogel/SF/CN/ Fe_3O_4nanocomposites	Ionic cross linking	1	200	47.44	23.94	[25]

Table S3. Recent studies have focused on MNPs and their nanocomposites and their applications in combating various bacterial strains

**Nanocomposite/ NPs**	**Size (nm)**	**Saturation Magnetization (emu/g)**	**Synthesis Method**	**Mechanism**	**Target Bacteria**	**Ref**
Fe_3O_4/Lignin	100-900	Not specified	Chemical reduction method	adsorption-release mechanism of ciprofloxacin from the NPs	*Salmonella enterica* and *Escherichia coli*	[26]
rGO/ Fe_3O_4 and bismuth doped Fe_3O_4 (FB-NPs), and Bi-rGO double doped Fe_3O_4 nanocomposites (FBR-NCs)	FB is 29.22, pristine Fe_3O_4 is 25.15, for FR (rGO doped Fe_3O_4) is 23.74 and for FBR (Bi-rGO double doped Fe_3O_4) is 21.35	Not specified	Co-precipitation method	Various gram-positive and gram-negative bacteria; bismuth-doped rGO reflects a high zone of inhibition. Mixed oxidation states and increased surface area enhance the activity against the bacterias.	Gram-positive *Staphylococcus aureus*<br>Gram-positive *Bacillus cereus*<br>Gram-negative *Pseudomonas aeruginosa*<br>Gram-negative *Vibrio cholerae*	[27]
Ag@Fe_3O_4	Ag core 70 , Ag@Fe_3O_4 220	72.8	Polyl reduction process	The antibacterial mechanism in Ag@ Fe_3O_4 includes the binding of Ag+ to the negative-charged bacterial cell wall, disturbing the membrane permeability and	*E. coli*	[28]

				degrading such important components, leading to the cell wall rupture and leakage of cellular fluid, causing the final bacterial death.<br>Antibiotic delivery, ROS generation		
Fe_3O_4@COF-AuNPs	385	55.5	Polyol reduction process	E. coli triggered a conformational change of hairpin aptamer probe and allowed the occurrence of TICA for ultrasensitive detection of E. coli, electrochemical biosensor	*E. coli*	[29]
MWCNT/Fe_3O_4/Cu(BDC)	Not mentiond	17.1	Ultra sound assisted method	The mechanism of action includes the interaction of the nanocomposite with bacterial cells, enhancing its antibacterial performance by releasing copper ions.	*E. coli* and *S. aureus*<br>Minimum inhibitory concentration and minimum bactericidal concentration assessments	[30]
Fe_3O_4@AgAu@PDA	Nanorods 10-30 nm long and 3-5 nm wide	29	Solvothermal method, In situ redox polymerization method	Plate colony counting method	*E. coli* and *S. aureus*	[31]
1T/2HMoS2/ Fe_3O_4/Ag	25 and 900 nm	-	Hydrothermal, coprecipitation and reduction techniques	MIC	*E. coli* and *S. aureus*	[32]
Fe_3O_4@SiO_2-Ag	Ag NPs have a diameter of 9.57 ± 2.96 nm, and the silica thickness is 7.95 ± 3.23 nm	36.42	Seed-mediated growth method	The antibacterial activities therein are associated with generating ROS.	The target bacterias are *E. coli* representing the gram-negative bacterial strains and *S. aureus*, which is of a gram-positive bacterial origin, while the antibacterial activities were assessed by investigating zones of inhibition.	[33]
Multi-branched Fe_3O_4@Au core@shell	30-100	Fe_3O_4 NPs: 50 emu/g<br>Fe_3O_4@SDS: 27 emu/g<br>Fe_3O_4@MgCuAl-LDH@CS: 16 emu/g<br>Fe_3O_4@MgCuAl-LDH@CS/Cu (II) catalyst: 12 emu/g	Template-free liquid-liquid interfacial growth reaction	The as-prepared nanocomposites effectively kill bacteria under the irradiation of a 980 nm laser.	*E. coli* and *S. aureus*	[34]
SnO_2@Fe_3O_4	34.57	-	Green synthesis method	Active oxygen species are generated which interact with the cell membrane of the bacteria and can easily pass through it.	*E. coli* and *S. aureus*, and *Pseudomonas aeruginosa (Pa)*	[35]
St/ Fe_3O_4/MIL-88(Fe) nanocomposite	Not specified	83.1, 25.6, and 20.3 emu/g for the synthesized Fe_3O_4 NPs,	Co precipitation and Hydrothermal assisted method	This improved antibacterial activity arises because of the release of a variety of antibacterial agents, including loaded drugs and metal	Target Bacteria: *E. coli* and *S. aureus*. The minimum inhibitory concentration (MIC) is determined as one of the	[36]

		St/ $Fe_3O_4$and St/Fe3O4/MIL-88(Fe), respectively		ions, from the nanocomposite.	detection methods.	
α -Fe_2O_3/Co_3O_4	25.34 (XRD)	82.49	Co-precipitation Method	This increases the total number of oxygen free radicals produced by the interaction of cytoplasmic water with the composite samples within the bacterial cell.	*B. subtilis*<br>*S. aureus*<br>*E. coli*<br>*S. typhi*	[37]
Fe_3O_4/ZnO NPs	Not specified	Not specified	Not specified	ROS generation, ion release	Not specified	[38]
Fe_3O_4/QSM/Ag nanocomposite	Size of the Fe_3O_4/QSM/Ag nanocomposite is determined to be 73 nm, with more than 90% of the measured particles having diameters smaller than 90 nm [1]. The mean hydrodynamic size of Fe_3O_4/QSM/Ag is 143.5 n	22.69	Co-precipitation method, followed by coating with quince seed mucilage (QSM), and immobilization of silver NPs (Ag NPs)	Damage bacterial cell membranes, cause the production of ROS, inhibit enzymes, and interfere with bacterial DNA.	Gram-positive and Gram-negative bacteria by using the method of agar diffusion	[39]
Fe_3O_4@CMC/Ag NPs	The average crystal size is 20.1 nm , mean diameter of the magnetic composite is 200 – 400 nm size distribution of around 300 nm	32.84 emu/g for Fe_3O_4, 28.6 emu/g for Fe_3O_4@CMC , and 30.56 emu/g for Fe_3O_4 @CMC/Ag NPs -7	Green synthesis and solvothermal method	The AgNPs disrupt the bacterial cell membrane, leading to loss of membrane integrity and leakage of the cellular contents, interference with some crucial enzymes, DNA replication, and finally death.	*Escherichia coli, Klebsiella pneumoniae, Citrobacter spp., Pseudomonas aeruginosa.* Detection: broth dilution method for determining MIC and MBC)	[40]
Magnetic Fe_3O_4 NPs coated with quince seed mucilage (QSM) and loaded with ciprofloxacin (CIP	Mean diameter of magnetite NPs is 13 nm; hydrodynamic diameter ranges from 81.9 to 119.2 nm	Fe_3O_4, Fe_3O_4@QSM , and Fe_3O_4@QSM -CIP are 59.16, 35.69, and 35.15 respectively.	Co precipitation method	Not specified	*Bacillus cereus, Staphylococcus aureus*- Gram-positive, and Gram-negative *Salmonella typhimurium*. Agar disk diffusion method of detection.	[41]
magnetic Fe_3O_4/berry bio-nanocomposites	15-18 nm	58.30	Co precipitation method	The mechanism of the antibacterial activity involves interaction with the bacterial cell membranes, making them impermeable and leading to non-respiration of the cells to cause lysis of cells.	*Escherichia coli* and *Staphylococcus aureus*, using the disk-diffusion method	[42]
Magnetic Fe3O4-loaded silver nanocomposites	151.07 ± 10 nm	Not Specified	Solvothermal method, Polydopamine coating method, Co-Precipitation method	The disruption of the respiratory chain of the bacteria, which causes an exhaustion of energy and, thus, the death of the bacterial cell	*E. coli, B. subtilis,* and *S. typh*	[43]
Fe_3O_4/Fenugreek seed/AgNPs	Fe_3O_4 NPs: 405.1 nm<br>Fe_3O_4/FSG nanocomposite: 268.9 nm<br>Fe_3O_4/FSG/Ag	Fe_3O_4: 68.18 emu/g<br>Fe_3O_4/FSG: 52.56 emu/g<br>Fe_3O_4/FSG/Ag: 29.98	Green synthesis method	Among them, the Fe3O4/FSG/Ag nanocomposite exhibited the best antibacterial efficiency to all four bacterial species. Because of the magnetic core and NPs of silver acting in synergy, it accounts for the inhibitory efficiency on bacterial	*Staphylococcus aureus, Bacillus cereus, Escherichia coli, Salmonella typhimurium*. The detection method used is a disk diffusion method.	[44]

	nanocomposite: 340.7 nm<br>DLS analysis indicated a size of 0.565 nm for the Fe_3O_4/FSG/Ag nanocomposite	emu/g		growth.		
CH-Fe_3O_4 NCs(Quercetin-loaded biosynthesized chitosan-grafted iron oxide NPs)	CH- Fe_3O_4NCs was calculated to be 16.16 nm	Not specified	Green synthesis method	The antibacterial action involves the electrostatic interactions whereby the negatively charged cell membrane of bacteria interacts with a positively charged metal oxide nanoparticle surface, hence influencing bacterial activities.	*Escherichia coli* and *Staphylococcus aureus,* respectively, by using the standard microbiological assays detection method.	[45]
Hollow magnetic spheres(Ag@ Fe_3O_4@HMS)	40 nm based on HRTEM and 350 nm by FESEM	14.73	Hydrothermal carbonization, Acid assisted assembly method, Impregnation and reduction method	The antibacterial activity of NPs can be explained by the use of silver NPs and zinc oxide NPs. AgNP formation may occur both intra- and extracellularly, while in extracellular synthesis, proteins and enzymes on the cell wall of bacteria reduce Ag+ to Ag0. Moreover, the high surface-to-volume ratio of the NPs increases their contact level with the surrounding environment, hence increasing their effectiveness against bacteria.	*Acinetobacter baumannii, Staphylococcus epidermidis*, *Proteus mirabilis, Staphylococcus aureus*, and *Pseudomonas aeruginosa* were detected by the agar diffusion method.	[46]
GO- Fe_3O_4 @NPVP-Ag	low pH: 700 (DLS)<br>Fe_3O_4: 15 (TEM)<br>GO-Fe_3O_4@NPVP-Ag: 12.7	Fe_3O_4: 71.9	co-precipitation	Immobilized AgNPs on the surface of GO nanosheets can release Ag + into the interior of the bacterial cell.	*E. coli*<br>*S. aureus*	[47]
Fe_3O_4@SiO_2 @mTiO_2-NH_2 @Ag bimetallic core-shell nanocomposite	Fe_3O_4@SiO_2@TiO_2 is 184 nm.<br>Fe_3O_4@SiO_2@mTiO_2 is 169 nm, AgNPs is 3 nm	Fe_3O_4 microspheres (64.78 emu/g), Fe_3O_4@SiO_2 (57.60 emu/g), Fe_3O_4@SiO_2 @TiO_2 (49.25 emu/g), and FSTN-Ag (46.17 emu/g)	Green synthesis And Extra cellular bacterial mediated	Inhibition of cell division and growth is due to interference with the use of oxygen as a result of interference by Ag+ released in the oxidation of metallic silver with molecular oxygen dissolved in the solution or culture via its interaction with functional groups of important enzymes and proteins within the cell respiratory chain.	*E. coli* and *S. aureus.* One detection method to evaluate anti-bacterial activity involves plate counting, where the quantity of bacterial colonies is a function of incubation time.	[48]
NC-Fe_3O_4-Ag nanocomposite	16.94, 17.45, and 18.08 nm	Not specified	In Situ Redox method	ROS generation that can damage the bacterial cell membrane	*Escherichia coli (E. coli)* and *Staphylococcus aureus*, method of detection used is the in vitro antibacterial activity test using LB agar media.	[49]
Fe_3O_4@MoS_2@PAA−EPL	Fe_3O_4-600 nm, Fe_3O_4@MoS2@PAA−EPL -910 nm	Fe_3O_4@MoS_2 @PAA−EPL are 71.7, 16.2, 4.2, and 4.3 emu g−1	Facile two-step hydrothermal method	It disrupts and causes damage to the bacterial membrane, leading to leakage of intracellular protein and inhibition of dehydrogenase enzyme activity.	*A. acidoterrestris*: Detection method involves fluorescence observation in treated bacteria.	[50]
Fe_3O_4@PEI NPs	74.16 nm	The saturation magnetization of Fe_3O_4 and Fe_3O_4@PEI	Trisodium citrate-assisted solvothermal method	by local heat and physical rotation on the accumulation of intracellular ROS upon its exposure in an AMF.	*E. coli* and *S. aureus*, detection methods have involved Live/dead staining followed by	[51]

		NPs was 74.8 emu/g and 59.5 emu/g			visualization using CLSM.	
Fe_3O_4/CuO/CS	75.97 nm	Fe_3O_4NPs was 64.70 emu/g, which was two times higher than that of the nanocomposite, which had a saturation magnetization of 32.35 emu/g	Ultrasound-assisted green method	Reactive oxygen species between the NPs and the bacterial cell membrane	*Escherichia coli* and *Staphylococcus aureus*	[52]
Fe_3O_4@Streptomycin	2.8-4.7	Not specified	Co-precipitation	Antibiotic delivery, ROS generation	*P. aeruginosa, S. aureus*	[53]
Fe_3O_4@Neomycin	2.8-4.7	Not specified	Co-precipitation	Antibiotic delivery, ROS generation	*P. aeruginosa, S. aureus*	[53]
Ag-MNP-hyd	Bare MNP: 40.5 nm<br>Bare hyd: 1199 nm<br>MNP-hyd: 1066 nm<br>Ag-MNP-hyd: 2265 nm	Not Specified	Coprecipitation method, Seed polymerization method	The mechanical friction and a certain degree of localized heating from MNPs, together with the antibacterial properties of silver, which may increase the disruption of the biofilm and release of silver within the immediate surroundings of bacterial biofilms.	*E. coli*, detection Method: The percent inhibition relative to the positive control after 24-hour incubation.	[54]
Fe_3O_4 NPs and Fe_3O_4@PDA nanocomposite	Fe_3O_4@PDA -25-30 nm larger than the Fe_3O_4 NPs	The cluster Fe_3O_4, Fe_3O_4@PDA and nanoplatform 1 were measured at 61.2 emu g^{-1}, 49.2 emu g^{-1} and 36.4 emu g^{-1}, respectively.	EDC/NHS-mediated conjugation method	NO (Nitric oxide) release upon irradiation with 808 nm light follows as an efficient antibacterial effect on target bacteria:	*S. aureus* and *E. coli.* Inhibition zone tests are used for its detection methods.	[55]
Cu/Cu2O-ZnO-Fe_3O_4	Cu_2O-ZnO-Fe_3O_4 - 50, 120, 180, and 230 nm	Not specified	Green synthesis. Chemical reduction method and PVP-Assisted sol gel	These materials function through nanostructure, ion release, and ROS effects that cause cell wall and membrane damage and induce the apoptosis of the cells:	*P. aeruginosa, S. aureus, B. subtilis,* and *E. coli* were used for the detection methodology; a traditional surface plate method was carried out for counting the viable cells.	[56]
Magnetic nanocomposite prepared by pyrolysis of biochar from sugar cane bagasse and magnetite NPs	The powdered sample was approximately 24 nm [1]. Additionally, the average diameter of the NPs observed through TEM was 5.49 ± 1.35 nm [4]. The nanocomposites exhibited a size distribution in the range of 100 − 200 nm	12.49	Green synthesis and Co-precipitation methods	Reactive species, including hydroxyl radicals (HO·), are produced under UV irradiation, which helps degrade the antibiotics ciprofloxacin and amoxicillin.	Gram-positive bacteria include *Streptococcus, Gonococcus*	[57]

Iron Oxide NPs (IONPs)	~10-100	Not specified	Coprecipitation	High reactive oxygen species generation against Gram-positive and Gram-negative bacteria	*Staphylococcus aureus, Escherichia coli*	[58]
MNPs-Fe	Not specified	Superparamagnetic above 60K	Green synthesis	Photothermal, ROS generation	*S. aureus, E. coli*	[59]
Iron oxide NPs	Not specified	Not specified	Coprecipitation	Antibacterial activity through ROS generation	*Staphylococcus aureus, Escherichia coli*	[60]
ZrO_2-ZnO NPs	Not specified	Not specified	Not specified	ROS generation, membrane damage	Not specified	[61]
$MgFe_2O_4$ NPs	~10-100	Not specified	Not specified	Disk diffusion and microbroth dilution tests demonstrating antibacterial activity	*Escherichia coli, Staphylococcus aureus*	[62]
Ag@Fe_3O_4 core-shell NPs	Not specified	Not specified	Wet chemical reduction, modified co-precipitation	ROS generation, silver ion release	*S. typhimurium, E. coli*	[63]
ZnO@Fe_3O_4	ZnO is 100 and 200 nm, ZnO@ Fe3O4 and Fe3O4 is less than 500 nm. The CMT-g-P3AP and CMT-g-P3AP/ZnO/Fe3O4 is 50.0 – 100 nm	ZnO@ Fe_3O_4 and Fe_3O_4NPs is 89.7 and 74.4	Co-Precipitation method	ROS generation and the attraction or disruption of negatively charged bacteria based on positive charge amine groups in P3AP are illustrated. When these materials were incorporated with the ZnO@Fe3O4 copolymer, there was increased antibiotic performance through synergism with all the components involved.	*E. coli* and *S. aureus*, the detection method used was the Kirby-Bauer disk diffusion assay	[64]
CuO@Fe_3O_4@Xanthan	40 to 60 nm	Not specified	Green synthesis method	Mode of action involves membrane disruption of cells, production of ROS, and infiltration through structures to lower the requirement of ATP for DNA repair mechanisms.	*Pseudomonas aeruginosa, Staphylococcus aureus, Salmonella spp.,* and *Escherichia coli*, checked through well diffusion method	[65]

References

[1] V. Narayanaswamy, S. Sambasivam, A. Saj, S. Alaabed, B. Issa, I. A. Al-Omari, I. M. Obaidat, "Role of Magnetite Nanoparticles Size and Concentration on Hyperthermia under Various Field Frequencies and Strengths,"*Molecules* **2021**, *26*, 796.

[2] O. M. Lemine, S. Algessair, N. Madkhali, B. Al-Najar, K. El-Boubbou, "Assessing the Heat Generation and Self-Heating Mechanism of Superparamagnetic Fe_3O_4 Nanoparticles for Magnetic Hyperthermia Application: The Effects of Concentration, Frequency, and Magnetic Field," *Nanomaterials (Basel)* **2023**, *13*, 453.

[3] S. Tabar Maleki, S. J. Sadati, Synthesis and investigation of hyperthermia properties of Fe_3O_4/HNTs magnetic nanocomposite," *Inorganic Chemistry Communications* **2022**, *145*, 110000.

[4] L. P. Ferreira, C. P. Reis, T. T. Robalo, M. E. Melo Jorge, P. Ferreira, J. Gonçalves, A. Hajalilou, M. M. Cruz, "Assisted Synthesis of Coated Iron Oxide Nanoparticles for Magnetic Hyperthermia," *Nanomaterials* **2022**, *12*, 1870.

[5] S. A. Moghadam Ziabari, M. Babamoradi, Z. Hajizadeh, A. Maleki, "The effect of magnetic field on the magnetic and hyperthermia properties of bentonite/Fe3O4 nanocomposite," *Physica B: Condensed Matter* **2020**, *588*, 412167.

[6] D. K. Mondal, G. Phukan, N. Paul, J. P. Borah, "Improved self heating and optical properties of bifunctional Fe_3O_4/ZnS nanocomposites for magnetic hyperthermia application," *Journal of Magnetism and Magnetic Materials* **2021**, *528*, 167809.

[7] P. Beigi, M. Babamoradi,"Preparation of Fe3O4@pectin nanocomposite hydrogel with high heating efficiency for hyperthermia applications," *Physica B: Condensed Matter* **2023**, *670*, 415360.

[8] A. Rezanezhad, A. Hajalilou, F. Eslami, E. Parvini, E. Abouzari-Lotf, B. Aslibeiki, "Superparamagnetic magnetite nanoparticles for cancer cells treatment via magnetic hyperthermia: effect of natural capping agent, particle size and concentration," *J Mater Sci: Mater Electron* **2021**, *32*, 24026–24040.

[9] A. L. Ramirez-Nuñez, L. F. Jimenez-Garcia, G. F. Goya, B. Sanz, J. Santoyo-Salazar, "In vitro magnetic hyperthermia using polyphenol-coated Fe_3O_4@γFe_2O_3 nanoparticles from Cinnamomun verum and Vanilla planifolia: the concert of green synthesis and therapeutic possibilities," *Nanotechnology* **2018**, *29*, 074001.

[10] R. A. Rytov, V. A. Bautin, N. A. Usov, "Towards optimal thermal distribution in magnetic hyperthermia,"*Sci Rep* **2022**, *12*, 3023.

[11] C. S. B. Dias, T. D. M. Hanchuk, H. Wender, W. T. Shigeyosi, J. Kobarg, A. L. Rossi, M. N. Tanaka, M. B. Cardoso, F. Garcia, "Shape Tailored Magnetic Nanorings for Intracellular Hyperthermia Cancer Therapy," *Sci Rep* **2017**, *7*, 14843.

[12] P. Kharey, M. Goel, Z. Husain, R. Gupta, D. Sharma, M. M, I. A. Palani, S. Gupta,"Green synthesis of biocompatible superparamagnetic iron oxide-gold composite nanoparticles for magnetic resonance imaging, hyperthermia and photothermal therapeutic applications," *Materials Chemistry and Physics* **2023**, *293*, 126859.

[13] S. R. Ansari, Y. del C. Suárez-López, T. Thersleff, L. Häggström, T. Ericsson, I. Katsaros, M. Åhlén, M. Karlgren, P. Svedlindh, C. M. Rinaldi-Ramos, A. Teleki,"Pharmaceutical Quality by Design Approach to Develop High-Performance Nanoparticles for Magnetic Hyperthermia," *ACS Nano* **2024**, *18*, 15284–15302.

[14] R. Eivazzadeh-Keihan, F. Farrokhi-Hajiabad, H. A. M. Aliabadi, E. Z. Ziabari, S. Geshani, A. Kashtiaray, M. S. Bani, B. Pishva, R. A. Cohan, A. Maleki, M. Mahdavi, "A novel magnetic nanocomposite based on alginate-tannic acid hydrogel embedded with silk fibroin with biological activity and hyperthermia application," *International Journal of Biological Macromolecules* **2023**, *224*, 1478–1486.

[15] T. Tatarchuk, A. Shyichuk, Z. Sojka, J. Gryboś, Mu. Naushad, V. Kotsyubynsky, M. Kowalska, S. Kwiatkowska-Marks, N. Danyliuk, "Green synthesis, structure, cations distribution and bonding characteristics of superparamagnetic cobalt-zinc ferrites nanoparticles for Pb(II) adsorption and magnetic hyperthermia applications," *Journal of Molecular Liquids* **2021**, *328*, 115375.

[16] N. Van Khien, C. Thi Anh Xuan, L. H. Nguyen, P. H. Nam, T. Thi Thao, "Role of citric acid coating in enhancing applicability of CoFe2O4 nanoparticles in antibacterial and hyperthermia,"*Materials Today Communications* **2024**, *38*, 107982.

[17] M. Yoga Darmawan, N. Imani Istiqomah, N. Adrianto, R. Marsel Tumbelaka, A. Dwi Nugraheni, E. Suharyadi,"Green synthesis of Fe3O4/Ag composite nanoparticles using Moringa oleifera: Exploring microstructure, optical, and magnetic properties for magnetic hyperthermia applications," *Results in Chemistry* **2023**, *6*, 100999.

[18] N. Danyliuk, S. Lischynska, T. Tatarchuk, V. Kotsyubynsky, V. Mandzyuk, "Magnetite nanoparticles synthesized using grape fruit extract: synthesis, morphology, hyperthermia application and catalytic activity in hydrogen peroxide decomposition," *Physics and Chemistry of Solid State* **2022**, *23*, 77–88.

[19] A. Hajalilou, L. P. Ferreira, M. E. Melo Jorge, C. P. Reis, M. M. Cruz, "Superparamagnetic Ag-Fe3O4 composites nanoparticles for magnetic fluid hyperthermia,"*Journal of Magnetism and Magnetic Materials* **2021**, *537*, 168242.

[20] S. M. Fotukian, A. Barati, M. Soleymani, A. M. Alizadeh, "Solvothermal synthesis of $CuFe_2O_4$ and Fe_3O_4 nanoparticles with high heating efficiency for magnetic hyperthermia application," *Journal of Alloys and Compounds* **2020**, *816*, 152548.

[21] F. Radinekiyan, M. R. Naimi-Jamal, R. Eivazzadeh-Keihan, H. A. M. Aliabadi, M. S. Bani, S. Shojaei, A. Maleki,"A magnetic cross-linked alginate-biobased nanocomposite with anticancer and hyperthermia activities," *Carbohydrate Polymer Technologies and Applications* **2024**, *7*, 100481.

[22] R. Eivazzadeh-Keihan, Z. Sadat, H. Aghamirza Moghim Aliabadi, F. Ganjali, A. Kashtiaray, M. Salimi Bani, S. Komijani, M. M. Ahadian, N. Salehpour, R. Ahangari Cohan, A. Maleki, "Fabrication of a magnetic alginate-silk fibroin hydrogel, containing halloysite nanotubes as a novel nanocomposite for biological and hyperthermia applications,"*Sci Rep* **2022**, *12*, 15431.

[23] S. Scialla, N. Genicio, B. Brito, M. Florek-Wojciechowska, G. J. Stasiuk, D. Kruk, M. Bañobre-López, J. Gallo, "Insights into the Effect of Magnetic Confinement on the Performance of Magnetic Nanocomposites in Magnetic Hyperthermia and Magnetic Resonance Imaging," *ACS Appl. Nano Mater.* **2022**, *5*, 16462–16474.

[24] C. Colli, N. Bali, C. Scrocciolani, B. Maria Colosimo, M. Sponchioni, E. Mauri, D. Moscatelli, S. Bandyopadhyay, "Zwitterionic thermoresponsive nanocomposites as functional systems for magnetic hyperthermia-activated drug delivery," *European Polymer Journal* **2024**, 113650.

[25] Z. Sadat, A. Kashtiaray, F. Ganjali, H. A. M. Aliabadi, N. Naderi, M. S. Bani, S. Shojaei, R. Eivazzadeh-Keihan, A. Maleki, M. Mahdavi, "Production of a magnetic nanocomposite for biological and hyperthermia applications based on chitosan-silk fibroin hydrogel incorporated with carbon nitride," *International Journal of Biological Macromolecules* **2024**, *279*, 135052.

[26] N. Y. Nguyen, H. V. T. Luong, D. T. Pham, L. N. H. Cao, T. T. Nguyen, T. P. Le, Drug-loaded Fe_3O_4/lignin nanoparticles to treat bacterial infections,"*International Journal of Biological Macromolecules* **2025**, *289*, 138868.

[27] C. Lakshmi, P. Balraju, N. Muthukumarasamy, V. Dhayalan, "Investigation of co-doped magnetite (Fe3O4) nanomaterials with reduced graphene oxide and bismuth for photocatalytic, supercapacitor, and antimicrobial applications,"*Electrochimica Acta* **2025**, *511*, 145376.

[28] Q. Shen, L. Zhang, Y. Zhao, X. Han, J. Gao, Y. Li, X. Zhu, T. Liang, T. Chen,"Magnetoplasmonic core–shell structured Ag@Fe_3O_4 particles synthesized via polyol reduction process rendering dual-functionality for bacteria ablation and dyes degradation," *Arabian Journal of Chemistry* **2025**, *18*, 106058.

[29] J. Zhang, M. Zhou, L. Yang, B. Huang, K. Lu, H. Wen, J. Ren, "Ultrasensitive electrochemical biosensor for bacteria detection based on Fe_3O_4@COF-AuNPs and trigging isothermal circular amplification,"*Sensors and Actuators B: Chemical* **2025**, *422*, 136609.

[30] N. Zokaei Golestan, S. Javanbakht, M. T. Nazeri, A. Shaabani,"Ultrasound-assisted synthesis of MWCNT /Fe_3O_4/Cu(BDC) nanocomposite: An efficient fast-release antibacterial carrier for potential suppository administration," *Materials Chemistry and Physics* **2025**, *332*, 130283.

[31] Q. Fang, J. Wang, S. Wu, K. C.-F. Leung, Y. Xu, S. Xuan, "NIR-induced improvement of catalytic activity and antibacterial performance over AuAg nanorods in Rambutan-like Fe_3O_4@AgAu@PDA magnetic nanospheres," *Journal of Hazardous Materials* **2023**, *445*, 130616.

[32] F. Sadeghi, M. Ghasemi, V. Soleimanian, H. Abbastabar Ahangar, "High-efficient anticancer, antibacterial, and adsorption activities of 1T/2H MoS2 nanoflowers decorated by Fe_3O_4 and Ag nanoparticles,"*Materials Chemistry and Physics* **2024**, *318*, 129252.

[33] Y. Romdoni, G. T. M. Kadja, Y. Kitamoto, M. Khalil, "Synthesis of multifunctional Fe_3O_4@SiO_2-Ag nanocomposite for antibacterial and anticancer drug delivery," *Applied Surface Science* **2023**, *610*, 155610.

[34] X. Lv, Z. Fang, Y. Sun, Y. Yang, X. Wang, Y. Chen, Y. Qin, N. Li, C. Li, J. Xu, H. Bao, "Interfacial preparation of multi-branched magneto-plasmonic Fe_3O_4@Au core@shell nanocomposites as efficient photothermal agents for antibacterial application,"*Journal of Alloys and Compounds* **2023**, *932*, 167712.

[35] G. G. Hasan, H. A. Mohammed, M. Althamthami, A. Khelef, S. E. Laouini, S. Meneceur, "Synergistic effect of novel biosynthesis SnO_2@Fe_3O_4 nanocomposite: A comprehensive study of its photocatalytic of Dyes & antibiotics, antibacterial, and antimutagenic activities," *Journal of Photochemistry and Photobiology A: Chemistry* **2023**, *443*, 114874.

[36] M. Abbasian, M. Khayyatalimohammadi, "Ultrasound-assisted synthesis of MIL-88(Fe) conjugated starch-Fe_3O_4 nanocomposite: A safe antibacterial carrier for controlled release of tetracycline,"*International Journal of Biological Macromolecules* **2023**, *234*, 123665.

[37] M. Bhushan, Y. Kumar, L. Periyasamy, A. K. Viswanath, "Antibacterial applications of α-Fe_2O_3/Co_3O_4 nanocomposites and study of their structural, optical, magnetic and cytotoxic characteristics,"*Appl Nanosci* **2018**, *8*, 137–153.

[38] K. Vijayalakshmi, L. Noor Ul Haq, "Microwave-sonochemical synergistically assisted synthesis of hybrid Ni-Fe_3O_4/ZnO nanocomposite for enhanced antibacterial performance,"*Materials Today Communications* **2021**, *26*, 101835.

[39] A. R. Gharaati, A. Allafchian, F. Karimzadeh, "Exploring the antibacterial potential of magnetite/Quince seed mucilage/Ag nanocomposite: Synthesis, characterization, and activity assessment," *International Journal of Biological Macromolecules* **2023**, *249*, 126120.

[40] M. H. Pourrafsanjani, R. Taghavi, A. Hasanzadeh, S. Rostamnia, "Green stabilization of silver nanoparticles over the surface of biocompatible Fe_3O_4@CMC for bactericidal applications," *International Journal of Biological Macromolecules* **2024**, *277*, 134227.

[41] M. Shirazi, A. Allafchian, H. Salamati, "Design and fabrication of magnetic Fe_3O_4-QSM nanoparticles loaded with ciprofloxacin as a potential antibacterial agent,"*International Journal of Biological Macromolecules* **2023**, *241*, 124517.

[42] P. Shanmugam, S. Boonyuen, Y. Tangjaideborisu, P. Na Nakorn, S. Tantayanon, R. Pothu, R. Boddula,"Anthocyanin Rich-Berry extracts coated magnetic Fe3O4 bionanocomposites and their antibacterial activity," *Inorganic Chemistry Communications* **2023**, *156*, 111291.

[43] R. Yang, B. Liang, D. Han, Z. Guo, C. Yang, J. Yang, Y. Qiu, Q. Li, S. Guo, J. Shi, X. Zhou, T. Qiang, T. Guo, "Synthesis and antibacterial activity of magnetic Fe_3O_4-loaded silver nanocomposites,"*Journal of Alloys and Compounds* **2024**, *973*, 172849.

[44] A. Allafchian, F. Karimzadeh, A. Valikhani, A. Seraj, "Enhanced antibacterial properties and magnetic removal of Fe_3O_4/fenugreek seed gum/silver nanocomposites for water treatment," *International Journal of Biological Macromolecules* **2023**, *251*, 126418.

[45] M. A. Alshehri, C. Panneerselvam, "Development of quercetin loaded biosynthesized chitosan grafted iron oxide nanoformulation and their antioxidant, antibacterial, and anti-cancer properties,"*Journal of Drug Delivery Science and Technology* **2024**, *101*, 106247.

[46] A. Farazin, M. Mohammadimehr, H. Naeimi, F. Bargozini, "Design, fabrication, and evaluation of green mesoporous hollow magnetic spheres with antibacterial activity,"*Materials Science and Engineering: B* **2024**, *299*, 116973.

[47] Q. Li, C. Yong, W. Cao, X. Wang, L. Wang, J. Zhou, X. Xing, "Fabrication of charge reversible graphene oxide-based nanocomposite with multiple antibacterial modes and magnetic recyclability,"*Journal of Colloid and Interface Science* **2018**, *511*, 285–295.

[48] J. Shi, J. Zheng, B. Liang, F. Song, J. Wang, S. Guo, H. Ge, Y. Gao, T. Zhang, "Silver-decorated amino-modified Fe3O4@SiO2@mTiO2 core-shell nanocomposites with catalytic and antimicrobial bifunctional activity,"*Colloids and Surfaces A: Physicochemical and Engineering Aspects* **2023**, *668*, 131402.

[49] S. A. Geleto, A. M. Ariti, B. T. Gutema, E. M. Abda, A. A. Abiye, S. M. Abay, M. L. Mekonnen, Y. A. Workie, "Nanocellulose/Fe_3 O_4 /Ag Nanozyme with Robust Peroxidase Activity for Enhanced Antibacterial and Wound Healing Applications," *ACS Omega* **2023**, *8*, 48764–48774.

[50] H. Jia, X. Zeng, R. Cai, Z. Wang, Y. Yuan, T. Yue, *J. Agric.* "Fabrication of Epsilon-Polylysine-Based Magnetic Nanoflowers with Effective Antibacterial Activity against *Alicyclobacillus acidoterrestris*," *Food Chem.* **2022**, *70*, 857–868.

[51] W. Liu, W. Pei, M. Moradi, D. Zhao, Z. Li, M. Zhang, D. Xu, F. Wang, "Polyethyleneimine Functionalized Mesoporous Magnetic Nanoparticles with Enhanced Antibacterial and Antibiofilm Activity in an Alternating Magnetic Field," *ACS Appl. Mater. Interfaces* **2022**, *14*, 18794–18805.

[52] N. Thi Huong, P. Thi Mai Huong, N. Thi Kim Giang, P. Thi Lan, V. Thanh Dong, C. Tien Dung, "Fe_3O_4 /CuO/Chitosan Nanocomposites: An Ultrasound-Assisted Green Approach for Antibacterial and Photocatalytic Properties," *ACS Omega* **2023**, *8*, 42429–42439.

[53] M. Caciandone, A.-G. Niculescu, V. Grumezescu, A. C. Bîrcă, I. C. Ghica, B. Ștefan Vasile, O. Oprea, I. C. Nica, M. S. Stan, A. M. Holban, A. M. Grumezescu, I. Anghel, A. G. Anghel, "Magnetite Nanoparticles Functionalized with Therapeutic Agents for Enhanced ENT Antimicrobial Properties,"*Antibiotics (Basel)* **2022**, *11*, 623.

[54] M. Wehbe, R. Kadah El Habbal, J. Kaj, P. Karam, "Synergistic Dual Antibacterial Activity of Magnetite Hydrogels Doped with Silver," *Langmuir* **2024**, *40*, 22865–22874.

[55] Y.-T. Yu, S.-W. Shi, Y. Wang, Q.-L. Zhang, S.-H. Gao, S.-P. Yang, J.-G. Liu, "A Ruthenium Nitrosyl-Functionalized Magnetic Nanoplatform with Near-Infrared Light-Controlled Nitric Oxide Delivery and Photothermal Effect for Enhanced Antitumor and Antibacterial Therapy,"*ACS Appl. Mater. Interfaces* **2020**, *12*, 312–321.

[56] W. Zhou, L. Fu, L. Zhao, X. Xu, W. Li, M. Wen, Q. Wu, "Novel Core–Sheath Cu/Cu_2 O-ZnO-Fe_3O_4 Nanocomposites with High-Efficiency Chlorine-Resistant Bacteria Sterilization and Trichloroacetic Acid Degradation Performance," *ACS Appl. Mater. Interfaces* **2021**, *13*, 10878–10890.

[57] N. Zulfiqar, R. Nadeem, O. A. Musaimi, "Photocatalytic Degradation of Antibiotics via Exploitation of a Magnetic Nanocomposite: A Green Nanotechnology Approach toward Drug-Contaminated Wastewater Reclamation,"*ACS Omega* **2024**, *9*, 7986–8004.

[58] T.-G. Zhang, C.-Y. Miao,"Iron Oxide Nanoparticles as Promising Antibacterial Agents of New Generation," *Nanomaterials* **2024**, *14*, 1311.

[59] D. G. García, C. Garzón-Romero, M. A. Salazar, K. J. Lagos, K. O. Campaña, A. Debut, K. Vizuete, M. R. Rivera, D. Niebieskikwiat, M. J. Benitez, M. P. Romero,"Bioinspired Synthesis of Magnetic Nanoparticles Based on Iron Oxides Using Orange Waste and Their Application as Photo-Activated Antibacterial Agents," *Int J Mol Sci* **2023**, *24*, 4770.

[60] M. A. Mubaraki, J. Ali, B. Khattak, F. Fozia, T. A. Khan, M. Hussain, M. Aslam, A. Iftikhar, I. Ahmad, "Characterization and Antibacterial Potential of Iron Oxide Nanoparticles in Eradicating Uropathogenic E. coli,"*ACS Omega* **2024**, *9*, 166–177.

[61] A. Precious Ayanwale, S. Y. Reyes-López, "ZrO2–ZnO Nanoparticles as Antibacterial Agents," *ACS Omega* **2019**, *4*, 19216–19224.

[62] A. M. El-Khawaga, M. Ayman, O. Hafez, R. E. Shalaby, "Photocatalytic, antimicrobial and antibiofilm activities of MgFe2O4 magnetic nanoparticles," *Sci Rep* **2024**, *14*, 12877.

[63] E. M. Sharaf, A. Hassan, F. A. AL-Salmi, F. M. Albalwe, H. M. R. Albalawi, D. B. Darwish, E. Fayad,Synergistic antibacterial activity of compact silver/magnetite core-shell nanoparticles core shell against Gram-negative foodborne pathogens," *Front. Microbiol.* **2022**, *13*, DOI 10.3389/fmicb.2022.929491.

[64] S. S. Mosavi, E. N. Zare, H. Behniafar, S. M. Nezhad, R. E. Neisiany, "Remediation of acetamiprid pesticide from contaminated water by antibacterial biosorbent based on carboxymethyl tragacanth-grafted-poly(3-aminophenol) decorated with ZnO@Fe3O4," *Environmental Research* **2024**, *252*, 118893.

[65] A. Ali Khafoor, A. Sabir Karim, S. Mohammad Sajadi, "The effect of Alanine and Morine functional agents on antimicrobial potential of green synthesized CuO@Fe_3O_4@Xanthan NCs using Pterocephalus nestorianus extract," *Results in Chemistry* **2024**, *9*, 101625.
